# Mathematical modelling reveals unexpected inheritance and variability patterns of cell cycle parameters in mammalian cells

**DOI:** 10.1371/journal.pcbi.1007054

**Published:** 2019-06-03

**Authors:** Marzena Mura, Céline Feillet, Roberto Bertolusso, Franck Delaunay, Marek Kimmel

**Affiliations:** 1 System Engineering Group, Silesian University of Technology, Gliwice, Poland; 2 Ardigen, Krakow, Poland; 3 Université Côte d’Azur, CNRS, INSERM, iBV, Nice, France; 4 Department of Statistics, Rice University, Houston, TX, United States of America; 5 Department of Bioengineering, Rice University, Houston, TX, United States of America; University of California Irvine, UNITED STATES

## Abstract

The cell cycle is the fundamental process of cell populations, it is regulated by environmental cues and by intracellular checkpoints. Cell cycle variability in clonal cell population is caused by stochastic processes such as random partitioning of cellular components to progeny cells at division and random interactions among biomolecules in cells. One of the important biological questions is how the dynamics at the cell cycle scale, which is related to family dependencies between the cell and its descendants, affects cell population behavior in the long-run. We address this question using a “mechanistic” model, built based on observations of single cells over several cell generations, and then extrapolated in time. We used cell pedigree observations of NIH 3T3 cells including FUCCI markers, to determine patterns of inheritance of cell-cycle phase durations and single-cell protein dynamics. Based on that information we developed a hybrid mathematical model, involving bifurcating autoregression to describe stochasticity of partitioning and inheritance of cell-cycle-phase times, and an ordinary differential equation system to capture single-cell protein dynamics. Long-term simulations, concordant with *in vitro* experiments, demonstrated the model reproduced the main features of our data and had homeostatic properties. Moreover, heterogeneity of cell cycle may have important consequences during population development. We discovered an effect similar to genetic drift, amplified by family relationships among cells. In consequence, the progeny of a single cell with a short cell cycle time had a high probability of eventually dominating the population, due to the heritability of cell-cycle phases. Patterns of epigenetic heritability in proliferating cells are important for understanding long-term trends of cell populations which are either required to provide the influx of maturing cells (such as hematopoietic stem cells) or which started proliferating uncontrollably (such as cancer cells).

## Author summary

All cells in multicellular organisms obey orchestrated sequences of signals to ensure developmental and homeostatic fitness under a variety of external stimuli. However, there also exist self-perpetuating stem-cell populations, the function of which is to provide a steady supply of differentiated progenitors that in turn ensure persistence of organism functions. This “cell production engine” is an important element of biological homeostasis. A similar process, albeit distorted in many respects, plays a major role in cancer development; here the robustness of homeostasis contributes to difficulty in eradication of malignancy. An important role in homeostasis seems to be played by generation of heterogeneity among cell phenotypes, which then can be shaped by selection and other genetic forces. In the present paper, we present a model of a cultured cell population, which factors in relationships among related cells and the dynamics of cell growth and important proteins regulating cell division. We find that the model not only maintains homeostasis, but that it also responds to perturbations in a manner that is similar to that exhibited by the Wright-Fisher model of population genetics. The model-cell population can become dominated by the progeny of the fittest individuals, without invoking advantageous mutations. If confirmed, this may provide an alternative mode of evolution of cell populations.

## Introduction

The cell cycle is a process leading to cell division. It plays a critical role in tissue growth, development and regeneration of multicellular organisms. It consists of two critical phases: the S phase, in which the cell replicates its DNA, and the M phase where it divides in two progeny cells (mitosis). These phases follow the G1 and G2 phases, respectively. After division, progeny cells usually re-enter the cell cycle and return to the G1 phase [1, 2]. Depending on a variety of factors, they may become quiescent (pass to the dormant G0 phase).

One of the important biological questions is how the dynamics at the cell cycle scale, which is related to family dependencies between the cell and its descendants, affects cell population behavior in the long-run. We address this question using a “mechanistic” model, built based on observations of single cells over several cell generation, and then extrapolated in time. We follow a paradigm recently expressed among others by Sandler et al. [3] and Dolbniak et al. [4] stating that stochastic processes in cells are associated with fluctuations in mRNA [5], protein production and degradation [6, 7], noisy partition of cellular components at division [8], and other cell processes. Variability within a clonal population of cells originates from such stochastic processes, which may be amplified or reduced by deterministic factors [9].

Independently of recent approaches, our work has been inspired by earlier work of Darzynkiewicz et al. [10], who analyzed cycling Chinese hamster ovary (CHO) cells using flow cytometry. They reported variability in G1 phase caused mainly by unequal division of cytoplasmic constituents into progeny cells, and the main conclusion was that the cell-cycle heterogeneity was generated mostly during cytokinesis and to a lesser degree during the G2 phase. These data influenced the mathematical models of Kimmel et al. [11], and Arino and Kimmel [12]. In these models, the heterogeneity has been generated only by unequal division or RNA or cytoplasm, with cell growth and the cell cycle duration being deterministic functions of the birth-size of cell. Models involving cell cycle duration stochasticity followed, with the most recent one being ref. [4]. The latter model is a precursor of the present one, yet with a more limited scope and based only on literature data.

Understanding of the complexity of cell-cycle dynamics and of the specific patterns of cell-cycle progression remains incomplete. Quantitative dynamic imaging combined with mathematical modelling has become an essential approach to understanding such complex dynamics [13]. The recently developed experimental FUCCI (fluorescent ubiquitination-based cell-cycle indicator) reporter system [14, 15] allows continuous imaging of cell-cycle progression in single live cells. In this system, two distinct proteins CDT1 and GEMININ, fused to fluorescent markers, indicate the G0/G1 and S/G2/M phases of the cell cycle, respectively.

FUCCI system has been used to investigate inheritance mechanisms in non-stimulated dividing mammalian cells [3], as well as in reoxygenated [16] and X-ray-irradiated cells [17]. In ref. [3] the authors analyzed the correlation of cell-cycle phase durations between family members. Variability in cell-cycle duration is ubiquitous, and sources of heterogeneity such as extrinsic and intrinsic noise [7] or unequal division [18] have been reported. Division times may also be epigenetically regulated [19].

In the present paper, we analyzed experimental and modelled cell pedigrees to determine patterns of inheritance of cell-cycle phase durations for aggregated G1 and S/G2/M phases, based on dynamic imaging of live NIH 3T3 cells. Based on this, we developed an integrated model involving bifurcation autoregression to describe cell proliferation and cell-cycle phase durations, and an ordinary differential equation (ODE) system to describe single-cell protein dynamics. The idea of bifurcating autoregression is that each line of descent from an ancestral cell follows the autoregression model (descendants inherit certain properties from the ancestor), while the inherited and environmental effects in progeny are correlated. We developed estimates of the parameters of bifurcating autoregression under lognormally distributed noise, given observed cell-cycle phase durations, and fitted single-cell protein trajectories to the ODE model. In this way we found correlations among parameters for single cells. We validated the model, using the cell pedigrees from dynamic imaging data.

Using the validated model, we employed long-term simulations to address the long-term behavior of the population, including homeostasis, memory of initial conditions and heritability. Specifically, we were interested in how regulation mechanisms of the cell cycle may contribute to propagation of new genetic or epigenetic variants in the cell population. This seems important because it has been established that many disease processes in living organisms were caused by the replacement of original cell diversity by clones which either proliferate without control (as in cancer), or dominate tissue-specific stem cells, limiting their resilience and ability to regenerate, as in aging bone marrow (see [20] and references therein). This observation has been explored in a number of deterministic and stochastic models (see review [21]). We summarized our findings using a version of the classical population-genetics Wright-Fisher model, with variable population size; examples and references can be found in [22, 23]. This approach is also related to the branching process paradigm, although our cell proliferation model is not a classical branching process [24].

*Remark* We employ the following vocabulary convention for family relationships in cell pedigrees. Suppose cell A divides into cells X and Y; then X divides into L and M, while Y divides into N and P. We call the progeny cells of the same parent cell sibling pairs. X and Y, L and M, and N and P, are sibling pairs. Cells whose parents are siblings we call cousin pairs. Thus, L and N, and M and P, are cousin pairs.

## Results

### Description of the mathematical model and the way it reproduces experimental data

The data at our disposal include single-cell observation of NIH3T3 cells using the FUCCI-2A system (see Methods section (*Experimental procedure*) for details), under two different Fetal Bovine Serum (FBS) concentrations. Cells were grown in constant conditions for 72 hours. During the experiment, films were recorded in randomly selected areas. We collected data from 123 cell lineages, including 890 individual cells from eight recorded films for 15% FBS, and 69 cell lineages including 224 individual cells from five recorded movies for 10% FBS. Based on experimental results we discovered that cell-cycle duration is shorter when higher FBS concentration is used. Faster progression of cell cycle is caused mainly by speeding up of S/G2/M phases progression. Differences between these two experiments are mainly visible (S7E Fig) in the fraction of cells entering dormancy (G0). Also see Supporting Information (S1 Text, *Sensitivity of durations of cell-cycle phases to serum stimulation*) for a more detailed description of these results. Further analysis was performed for 15% FBS data, since the sample size was significantly higher than in the 10% FBS experiment, and our model is not focused on dormant cells.

#### Cell cycle kinetics in the pedigree as bifurcating autoregressive process

**Fig 1:**
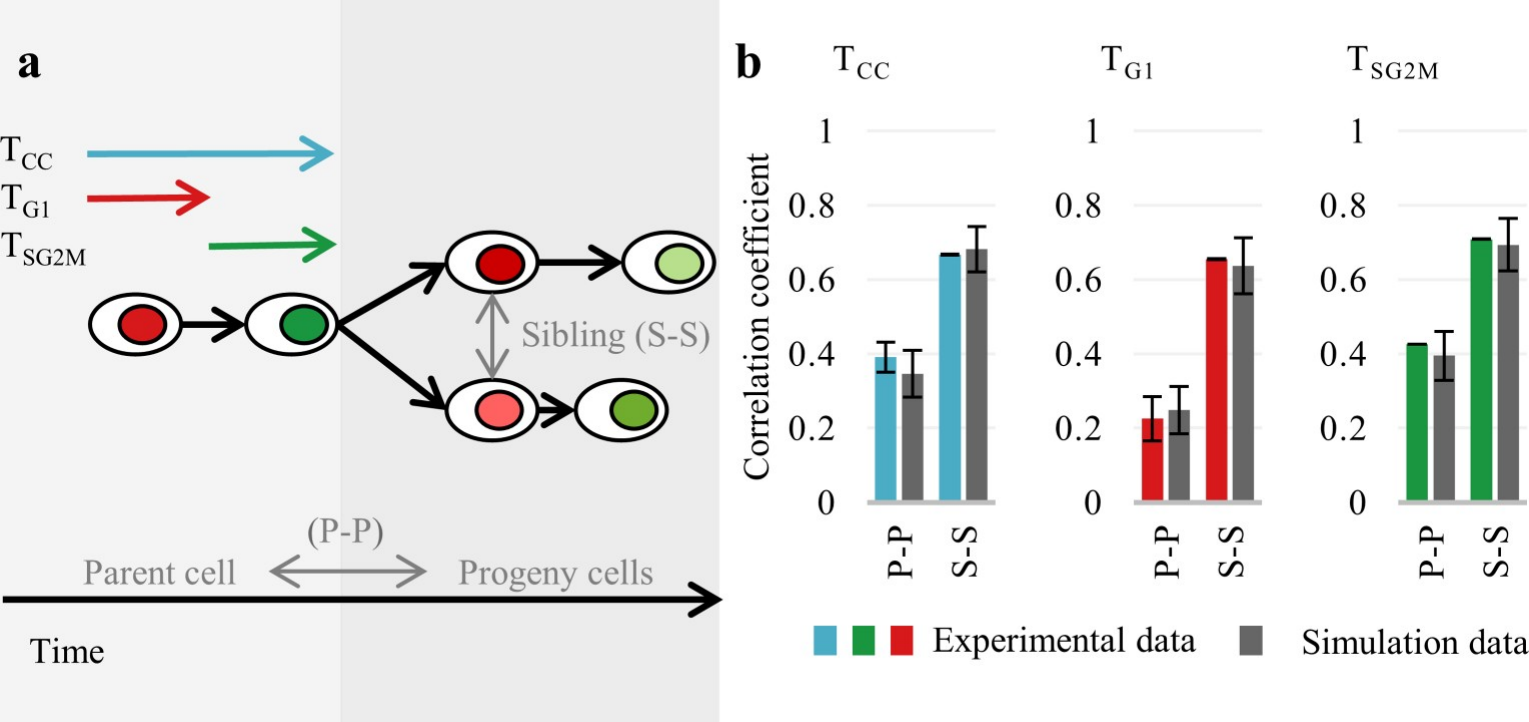
Family relations among cells observed *in vitro*. (A)Time-lapse microscopy permits the tracking of individual cells, identifying division moments, family relations, cell cycle and cell-cycle phase duration, and protein concentration at the time of each observation. Introduced notations and colors indicate cell cycle (CC, blue), G1 phase (G1, red) and S/G2/M phases (green). Corresponding simulation data are represented by the gray color. (B) Correlations between family members based on experimental and simulation data. Estimation of standard deviations from experimental data is described in detail in Supporting Information (S1 Text, *Methods*). Simulation results for the autoregression bifurcation model are described in detail in Methods.

Time-lapse microscopy allows tracking of individual cells and identifies family relations among cells (Fig 1A). Using this information we investigated the inherited and environmental causes of cell cycle (CC), cell-cycle phases length (G1 and S/G2/M phases) and protein dynamics variability. We observed strong positive correlation of cell cycle between siblings, whereas the same correlation for parent and progeny (***θ***) was significantly smaller (Fig 1B). Correlation between cell-cycle phases follow the same pattern, but the correlation between durations of G1 phases is always lower than correlation between durations of S/G2/M.

Our results suggest that progeny cells inherit properties from their parents (Fig 1B). Consistent with this, we observed even stronger correlations between siblings. In the literature, such correlations are frequently explained by external factors affecting the cell-cycle length, such as environmental conditions, among-cell communication, neighborhood effect or the age of cells (see ref. [25] for the NIH 3T3 cells). In our data, after two generations the correlation disappears, as shown by low-correlation coefficients between grandparent and grand-progeny cells (S3A Fig).

As is later demonstrated, experimental results are consistent with the bifurcating autoregression model, in which the progeny cells inherit random but correlated fractions of a given feature of the parent cell [26]. Bifurcating autoregression has been successfully used previously to fit cell-cycle kinetics data recorded as magnetic tape videos [27]. The approach presented in the current paper is more complex; it includes modeling of phases of cell cycle and protein concentration and is based on a significantly larger dataset.

Analysis of cell-cycle kinetics in the pedigree, with results for the third generation of cells (cousins, grandparent—grand-progeny), including the physical proximity and cell birth time which do not affect cell-cycle duration, are presented in Supporting Information (S1 Text, *Hereditary and environmental causes of cell cycle length variability based on the 15% FBS experiment*).

#### Modelling the pattern of dependence between G1 and S/G2/M phases of cell cycle

**Fig 2:**
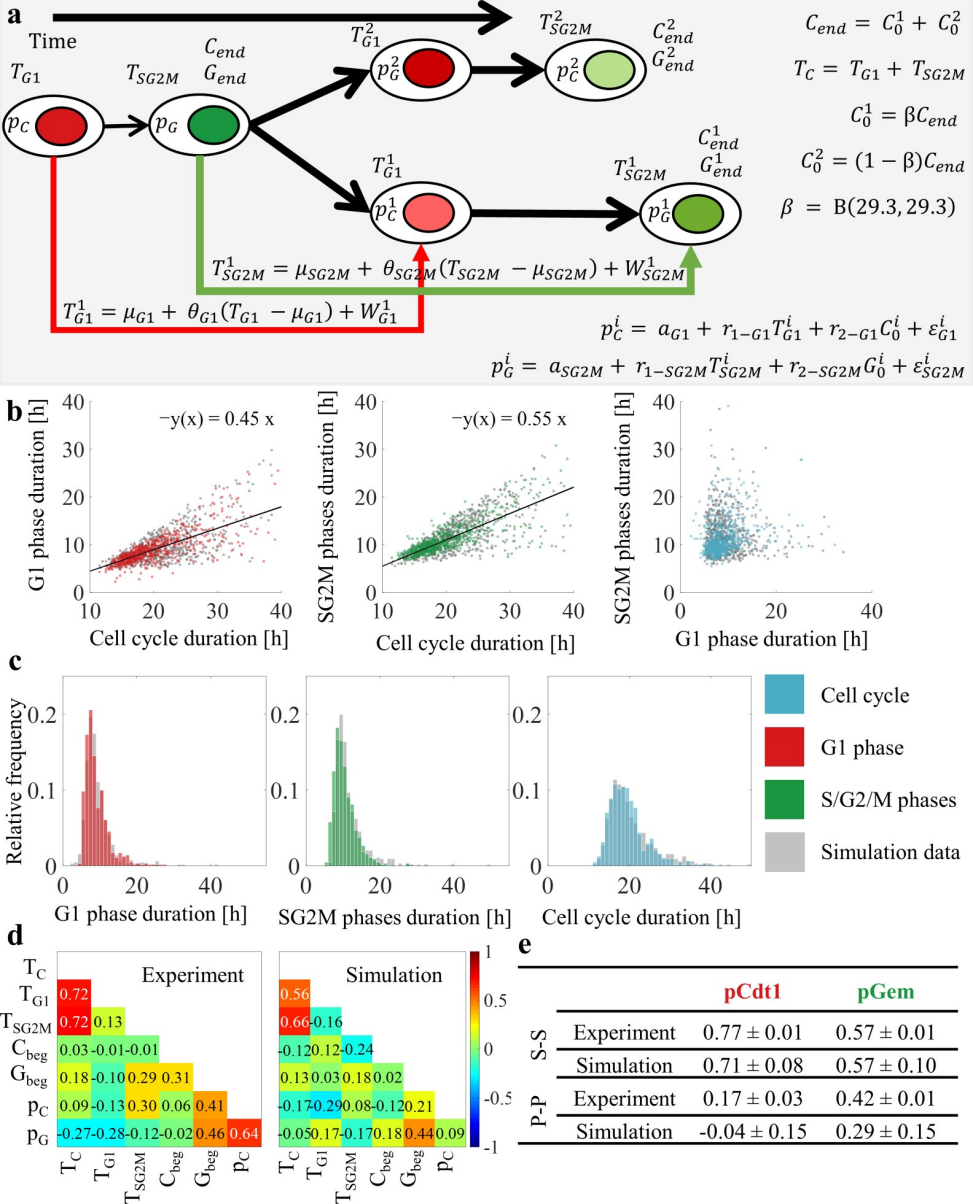
Analysis of protein dynamics based on experimental and simulation data. (A) Schematics of the model. In the population, each cell is characterized by seven parameters. At division, the parent-cell mass (*C*_*end*_, *G*_*end*_) is randomly split between the two progeny cells, according to the expression $C_{0}^{1}=\beta C_{end},C_{0}^{2}=(1-\beta {)C}_{end}$ in which *β* is the random variable. Cell-cycle phase durations ($T_{G1}^{i},T_{G1}^{i}$) for progeny cells (*i* is progeny number) are calculated using autoregression bifurcating models. $p_{C}^{i},p_{G}^{i}$—production rate of Cdt1 and Geminin proteins, are calculated using linear regression models, $T_{C}^{i}$—cell cycle duration, *T*_*G*1_,*T*_*SG*2*M*_—G1 or S/G2/M phases length of the parent cell, *μ*_*G*1_,*μ*_*SG*2*M*_–mean G1 or S/G2/M cell cycle phases length, *θ*_*G*1_,*θ*_*SG*2*M*_–relation between parent and progeny G1 or S/G2/M cell cycle phases length, $W_{G1}^{i},W_{SG2M}^{i}$ – random variables from bivariate lognormal distributions (common mean zero, common variances and correlation coefficients). We abandoned the originally assumed bivariate normal distribution to lognormal because of the positive skewness of the distributions of cell-cycle duration. (B) Comparison of linear relationships between the total division time and the duration of phases for experimental and simulation data. Solid black lines show the fitted linear relations of the form *y* = (*slope*) × *x*. (C) Comparison of distributions of the cell cycle and the cell-cycle phase duration for experimental data and modelling results. (D) Looking for probable regulatory mechanisms. Data-derived and simulation-based correlations between pairs of variables characterizing the protein trajectories. (E) Data-derived and simulation-based correlations between production parameters of family members.

Previously [3], the bifurcating autoregression model was applied to cell-cycle duration data, but not to dependence between durations of cell-cycle phases. We investigated the statistical dependence of cell-cycle phase durations and overall cell-cycle times, and found a strong positive correlation between durations of cell-cycle phases and cell-cycle times (Fig 2B). However, there exists only a low correlation between durations of G1 and S/G2/M phase length (Fig 2B). Based on detailed analysis of presented results, including clustering of cells with different progression rates in G1 and S/G1/M (Supporting Information, S1 Text, *Statistical dependence of cell cycle phase durations and cell cycle times*), we decided that inheritance of the durations of the phases of the cell cycle can be modelled independently. Accordingly, we used the bifurcation autoregression model to estimate the durations of the G1 and S/G2/M phases in related cells (cf. schematics of the model in Fig 2A., and *Estimation of Parameters* using the method of moments in Methods). Patterns in the experimental data are well reproduced by the results of modelling. In Fig 2C, we presented the distributions of the experimental and simulated data, and in Fig 2B the noise-perturbed linear relationships between the total division time and the durations of the G1 and combined S/G2/M phases in simulation data. Results confirm that we can treat durations of cell-cycle phases independently, which implies that lengthening one phase does not imply lengthening of other phases.

#### Modelling protein dynamics in individual cells

**Fig 3:**
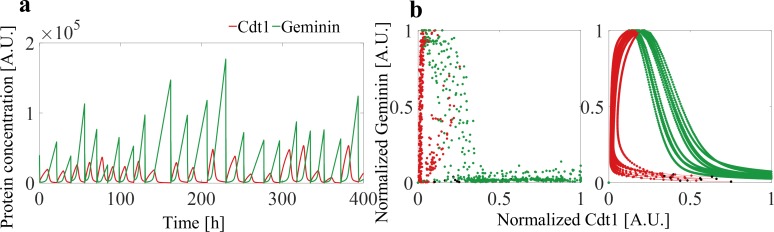
Results of long term simulations. (A) Dynamics of the time series of the Cdt1 and Geminin protein contents in a randomly chosen lineage of descendants of the ancestor cell. (B) Phase portrait of experimental and simulation data. Ten trajectories of cell cycle were randomly selected from the experiment (*left*) and simulation (*right*) data. In both cases, fluorescence levels were normalized to maximum values.

As we described in ref. [4], we fitted experimental trajectories of individual cells to a model in the form of a system of ordinary differential equations (ODE) and calculated correlations among production parameters, duration of phases of cell cycle and initial levels of proteins. Each cell in the population is described by seven parameters with cell-dependent stochastic values. Based on the matrix of correlations presented in Fig 2D (left part) we proposed a procedure described in the Methods section and in Fig 2A, with the added noise being correlated between siblings. Correlated noise reproduces weak correlation of protein dynamics between parent and progeny, and strong correlation of siblings evident from correlation matrices of protein levels in related and unrelated cells (diagonal area, S5 Fig). Comparison of the correlation between protein production parameters assumed in the simulations and those recovered directly from the experimental data (see Methods, Estimation of parameters) is presented in Fig 2E. Detailed scatterplots of experimental and simulated data (S11 Fig) demonstrate the accuracy of the model. Simulation results reproduced most of characteristics observed in experimental data (Figs 1–3).

### Wright-Fisher model and the cell-cycle model

#### Long-term simulations: Memory and transients

We have run the model for a large number of cell generations (50 generations), recording the protein contents in cells of the resulting pedigree at constant time intervals. Simulation was started from a single ancestor cell with defined or randomly sampled parameters, and the fates of cells from the resulting populations have been recorded. During cell division, the proteins are unequally distributed among progeny cells [4]. Direct measurement of the extent to which division of proteins is unequal is difficult due to degradation of the FUCCI markers in late M phase. We performed simulations to calibrate the impact of this distribution’s width on the dynamics of protein levels in the progeny cells. Strong asymmetry in protein division does not affect the regulation mechanisms modelled, and therefore we used the parameters proposed in ref. [4], calculated for mammalian cells. A sample long-term simulation and resulting phase portraits are presented in Fig 3. Stochasticity of cell-cycle duration and protein production parameters result in large variability in protein expression, attenuated by regulatory mechanisms.

Phase portraits are the usual way of depicting systems dynamics in general [28]. Fig 3B depicts phase portraits based on experimental data (*left*) and on simulation results (*right*), which coincide quantitatively for most observed cell trajectories, with the qualitative pattern being similar in almost all cells. Discrepancies are likely to be due to high level of measurement-related noise in the cells followed experimentally. An example of such “noisy” measurement is presented in S12 Fig

We designed long-term experiments to verify if the initial ancestor cell has an impact on cell cycle durations in later generations. Our first experiment was based on simulations for 400 generations of a randomly chosen line of descent originating from a single cell. Histories of cell-cycle times for single cells showed stabilization properties (S8 Fig). The influence of the ancestral cell-cycle time became dissolved in subsequent generations.

Our second experiment was based on simulations of a growing population, started, as before, from single ancestor cells with different cell-cycle lengths. In two extreme cases (initial cell-cycle time 13.6 h and 61.3 h), large differences in the population growth rate were observed. Within the interval from 0 to 200 h, during which the cell-cycle duration in both populations returned to the stationary distributions, the descendants of the cell with the short cell-cycle length have formed a subpopulation 40 times larger than the descendants of the cell with the long cell-cycle duration. If these two subpopulations were pooled, the one originating from the ancestor with the shorter cell-cycle length would dominate the other.

**Fig 4:**
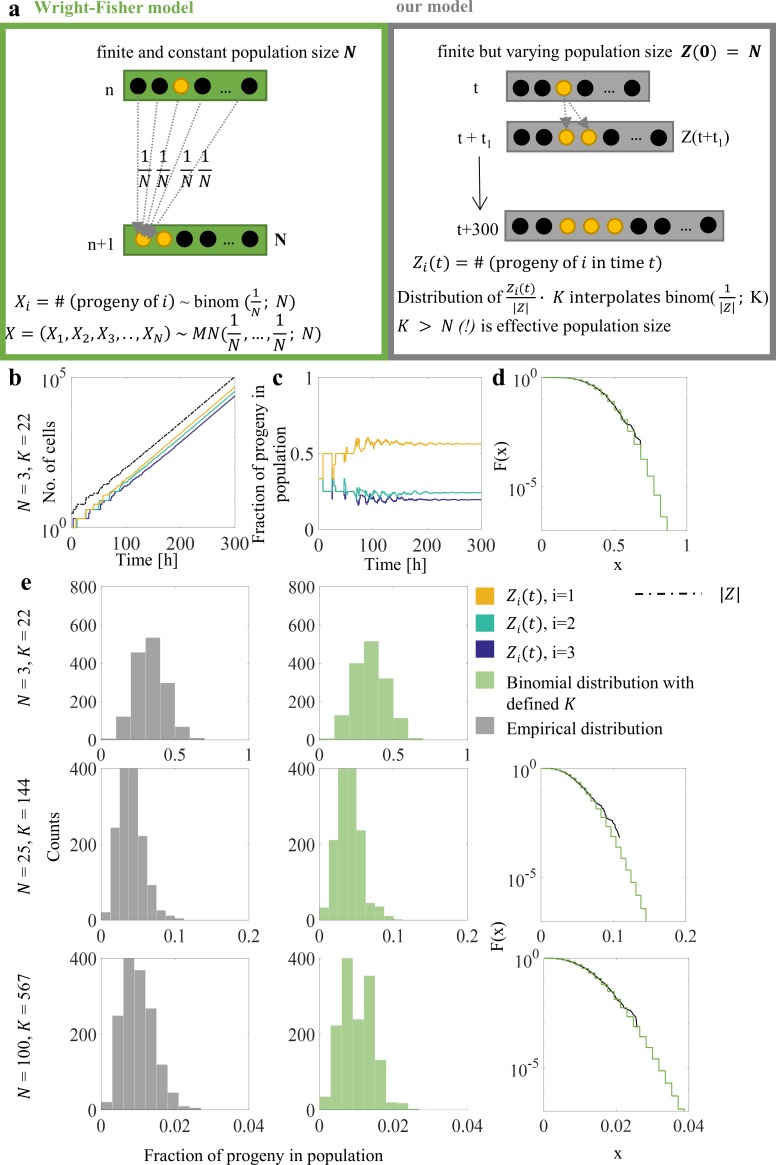
Comparison of simulation results obtained by proposed model and Wright-Fisher model. (A) Main assumptions of Wright-Fisher model and our model are presented in separate boxes. The most important difference concerns population size, constant in Wright-Fisher and variable in our model. Series of *in silico* experiments were performed with different initial population sizes of 3, 25, and 100 cells drawn from previously generated populations. Each cell was characterized by different cell-cycle time and at the time 0 cells were not synchronized (i.e. cells were spread over different cell-cycle phases). (B–D) Example of performed simulations for initial population with *N* = 3. B) Descendants of ancestor cells are identified and counted. Total population size is marked by black dashed line. C) The fractions of the progeny in population were calculated. We analyzed the fraction of progeny in the population at time 300, but the level is determined after *t* = 200. Simulations were repeated until required sample size was obtained. D) Cumulative distribution functions for simulation data and Wright-Fisher model. (E) Comparison of simulation data and Wright-Fisher model for three different values of initial cell count *N*. Histograms of simulation datasets (*left*) and random numbers drawn from estimated binomial distribution with *K* (*right*) represent the fractions of progeny in population after 300 h of simulations. Cumulative distribution functions for both cases are also presented to compare the tails of distributions.

*Epigenetic drift in proliferating cell population*. The effect dominance has on the descendants of “fastest cycling” cells, described in the last paragraph of the previous section, is not deterministic (see further on). Our results show that it is similar to genetic drift, and we use a working term “epigenetic drift”. The classical form of genetic drift used in population genetics [29], is embodied by the Wright-Fisher model (Fig 4A), which assumes a time constant population size equal to *N* and discrete generations. Each (*n*+1)-st generation individual is a progeny of a randomly chosen individual of the *n*-th generation. As a result, the number of progeny of any individual is binomially distributed, and the random vector *X* of progeny counts of all *N* individuals has multinomial distribution, i.e. *X*~*MN*(*N*^−1^,…,*N*^−1^;*N*). Eventually, for time *n* large and inversely related to *N*, the population is dominated by the progeny of a single individual (effect known as “fixation”). It is also known [29] that if the population size *N* = *N*(*n*) varies with *n*, then the Wright-Fisher model including fixation holds approximately, with “effective” population size being equal to the harmonic mean of *N*(1),*N*(2),…,*N*(*n*). Our simulation results show similar effects, however, with effective population sizes deviating from simple harmonic means (also, see Methods).

Fig 4 contains a summary of hypotheses and results. Assumptions of the Wright-Fisher model and our model are presented in separate boxes (Fig 4A). The most important difference concerns population size, constant in Wright-Fisher model and variable in ours. In Fig 4B–4E, the outcomes of a series of *in silico* experiments, performed with different initial population sizes, are shown. In these simulation runs, 100, 25, 10 or 3 cells were drawn from previously generated populations. Each cell had different cell-cycle length and at *t* = 0 cells were not synchronized (cells were in different cell-cycle phases). The number of descendants for each initial cell was determined (marked by distinct colors in Fig 4B, for the case *N* = 3). Total population size was marked by a black dashed line (Fig 4B). The fraction of each progeny in the population was calculated (colors in Fig 4C correspond to colors in Fig 4B). We analyzed the fraction of descendants in the population at time 300, while in general this fraction stays constant already after *t* = 200. Simulations were repeated until appropriate sample size was obtained. Outcomes are presented in histograms in Fig 4E; the mean value is specified for each histogram. Random numbers drawn from estimated binomial distribution with coefficient *K* values as specified in the legend are presented in Fig 4E. Distribution tail functions compared to the binomial are presented in Fig 4E.

Based on the Wilcoxon’s test, there is no significant difference between the normalized frequencies and the binomial distribution. The estimates of covariances (Table 1) are not equal to theoretical expected values, although they are of similar order, except for Dataset 1, which includes only 3 ancestral cells. Correlation coefficients have very skewed distributions [30], which can be one of the reasons for the discrepancy. Another possible reason is deviation from multinomial sampling. Wright-Fisher model is a special case of the so-called Cannings model, which allows more general (not necessarily multinomial) sampling distributions, and may result in different correlation coefficients. Cannings model is not frequently used, however it was applied to model stem cells in human colon [31]. Based on current data, it seems difficult to determine which of these two models might be more appropriate.

**Table 1:** Comparison of data-estimated and Wright-Fisher model-predicted parameter values, variances, and correlation coefficients, based on simulated genealogies of proliferating cells.

	Parameters			V(*X*_*i*_)		*ρ*(*X*_*i*_, *X*_*j*_)	
	N	K (Estim.)	Harmonic mean	Estimated	Expected	Estimated	Expected
**Dataset 1**	3	22	32	0.01005	0.01005	-0.04	-0.50
**Dataset 2**	10	59	104	0.00153	0.00153	-0.01	-0.11
**Dataset 3**	25	145	256	0.00027	0.00027	-0.02	-0.04
**Dataset 4**	100	567	1063	0.00002	0.00002	-0.08	-0.01

### How correlation between related cells affects population growth

Interpretation of results obtained by cell-cycle model may be difficult because it is not known which effects are caused by population growth and which by correlations among family members. To separate these two effects we used a simplified model of cell cycle which does not include correlations among family members. In the model, cell cycle phase duration of each individual was drawn from lognormal distribution and the parameters were estimated based on experimental data. Long-term experiments were designed for different initial numbers of ancestors. Results for four cases introduced in Table 1 are presented in Table 2. Estimated effective population size (*K*) is almost twice as high as that obtained using the complete model, which includes correlations among family members, and close to harmonic mean value calculated from simulated genealogies.

**Table 2:** Comparison of data-estimated and Wright-Fisher model-predicted parameter values, variances, and correlation coefficients, based on simulated genealogies of proliferating cells obtained by model which does not include family relations; the cell-cycle length for each cell is a random variable, drawn from lognormal distribution.

	Parameters			V(*X*_*i*_)		*ρ*(*X*_*i*_. *X*_*j*_)	
	*N*	*K* (Estim.)	Harmonic mean	Estimated	Expected	Estimated	Expected
**Dataset 1**	3	39	31	0.005624	0.005624	-0.03	-0.50
**Dataset 2**	10	115	101	0.000783	0.000783	-0.00	-0.11
**Dataset 3**	25	285	262	0.000135	0.000135	-0.02	-0.04
**Dataset 4**	100	1118	1051	0.000009	0.000009	-0.08	-0.01

**Fig 5:**
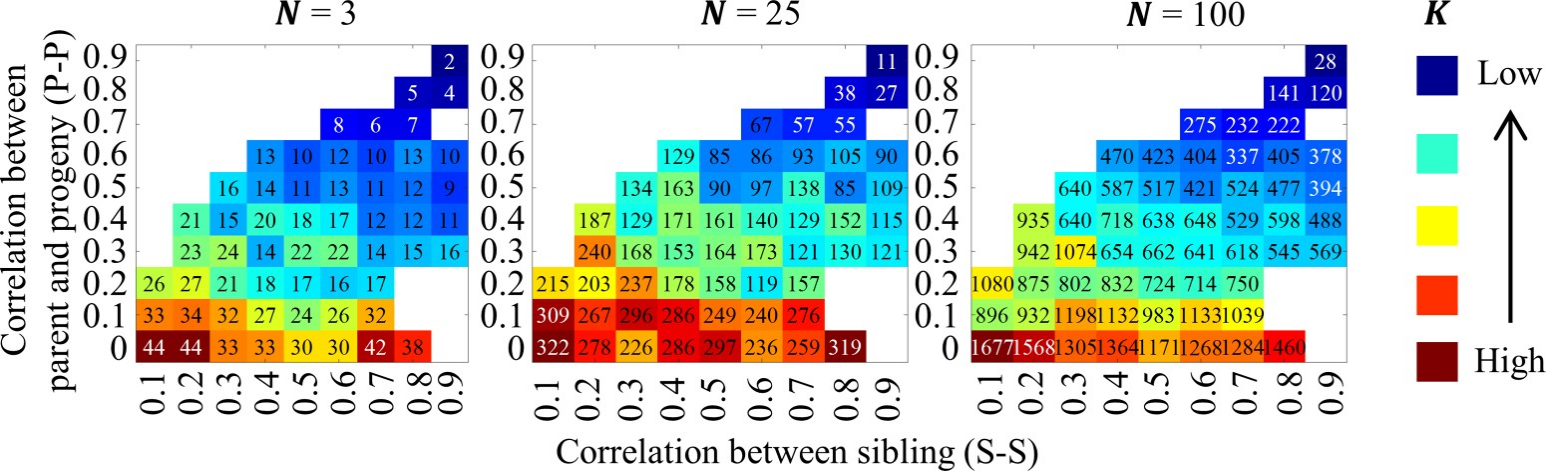
Effect of epigenetic drift on population development. The effective population sizes *K* were estimated for different values of parent-progeny and sibling correlation coefficients.

To verify how the value of effective population size depends on correlations among family members we used the model presented in this paper, which can reproduce behavior of populations with varying correlations among family members. Parameters of the model were estimated, simulations were performed and *K* values were estimated for all possible cases for three different numbers of ancestor cells (Fig 5).

## Discussion

In the current paper, we related the experimental data from dynamic imaging of proliferating NIH 3T3 cells to a mechanistic view of the cell cycle and dynamics of accumulation and decay of proteins expressed specifically in different cell-cycle phases. The data included recorded observations of cell pedigrees started from single ancestors and then followed for several divisions (72 h). Assuming constant conditions, it seems possible to reconstruct the long-term dynamics of such cells by building a relevant mathematical model, estimating it based on short-term data, and extrapolating the results *in silico* to longer times.

Importantly, we investigated existence in our simulations of drift-like effects similar to those exhibited by the Wright-Fisher model of population genetics. In brief, our findings support the following paradigm of interplay of regulation mechanisms in the cell cycle and long-term proliferation:

1. There exist intrinsic sources of variability in eukaryotic cells, such as unequal division and variable (random) durations of cell-cycle phases. The term “intrinsic” is understood here as referring to dynamics dependent on processes within cells as opposed to those dependent on cell environment (see [32] for a different perspective).
2. Descendants of cells with extreme properties, such as very short cell cycle, gradually converge to follow stationary distribution, after a possibly protracted transient.
3. Duration of the transient is sufficient for the descendant of the “extreme” cells to be over- or under-represented in the after-transient population.
4. Representation of subpopulations traceable to specific ancestral cells follows patterns similar to a variant of Wright-Fisher model with varying population size. In other words, proliferating cell populations experience a specific form of epigenetic drift in addition to mutation and selection.
5. The level of correlation among family members has a significant role in population growth, and modulates the effect of drift.

Two important points regarding our approach are (1) selection of the cell system, and (2) generalizability to other cells. The NIH3T3 cells grow in flat-dish cultures, which makes it possible to track progression through the growth and division cycles. Cells grown in suspension pose unsurmountable difficulties in this respect. All publications known to us, which employed growth and division tracking, used flat-dish cultures. The list includes Chinese Hamster Ovary cells [11], NIH 3T3 cells, H1299 non-small cell lung cancer cells [4, 33], and L1210 mouse lymphocytic leukemia cell [3]. Using flat-dish cultures can be considered a limitation. However, it can also be argued that suspension cultures are even less similar to cells in physiological conditions, which always require substrate for proliferation. In addition, mathematical and statistical analyses of the data in papers listed above indicate, despite differences in details, qualitatively similar patterns of cell-cycle regulation.

### Modelling the multivariate statistics of cell cycle and FUCCI protein dynamics

Many models of cell-cycle progression have been proposed in the literature: age-structured cell population models [34], branching processes [4, 34], transition probability models [35–37] and other novel models [38], many of them based on experimental data [4, 13, 34, 38, 39]. The importance of developing a fully integrated model with different sources of noise and heterogeneity was discussed in ref. [40]. This motivated us to develop heterogeneous population-growth models with protein dynamics included, such as the model in the present paper, and an earlier model in ref. [4].

The important feature of our model is that it reproduces most of the characteristics observed in experimental data, as is evident from Figs 1, 2 and 3. Our model is based in part on bifurcating autoregression [26] applied to cell-cycle phases and on ideas concerning cell-cycle regulation and unequal division that were developed by many authors; specifically, we refer here to the model by Kimmel et al. [11]. In the model, chance and deterministic elements contribute to its ability to accurately fit multiple facets of cell-cycle kinetics in a heterogeneous cell population.

This type of model may be important not only for understanding the kinetics of cell proliferation but also for testing of the individual responses of the cell to stimuli, especially when such a response is cell cycle-dependent. The models should also be useful for predicting the growth rates of populations consisting of subgroups with different properties and/or in which epigenetic effects are strong. We know that tumor growth is the consequence of competition among a few cell populations. It seems that even a small difference in cell characteristics, such as the cell-cycle proliferation time [41], may increase the ability of a cancer to survive chemotherapy and re-enter the cell cycle.

### Long-term behavior of model trajectories including homeostasis and “memory” of initial conditions

One of the features of our approach is integration of *in vitro* experiments with statistics and *in silico* simulations. The function of the latter is to understand the long-term behavior of cell population given the set of rules (i.e. the mathematical model) inferred using statistical tools, based on a limited-time *in vitro* experiment. Questions that can be answered in this way include the homeostatic properties of the population growth. Specifically, do the rules of cell growth and division lead under constant environmental conditions to stabilization of the distributions of important cell characteristics, such as cell-cycle time and durations of cell-cycle phases, as well as concentrations of cell proteins? A related question concerns the nature of the transients that emerge after a cell with extreme parameters becomes an ancestor of its own population. If this cell has a short cell cycle, will its progeny tend to dominate the population?

Based on simulations, in two extreme cases of initial cell-cycle time 13.6 h and 61.3 h, large differences in the population growth rate have been observed. Within the interval from 0 to 200 h, during which the cell-cycle duration in both populations returned to the equilibrium distributions, the descendants of the cell with the short cell-cycle length have formed a subpopulation *n* = 40 times larger than the descendants of the cell with the long cell-cycle duration. If these two sub-populations were mixed, the one originating from the ancestor with the shorter cell-cycle length would dominate the other. In bacterial cells, the importance of long-term dynamics of cellular populations was considered in recent studies [42, 43], in which mathematical models were supported by biological experiments using *E*. *coli* strains. In the first paper [42], authors discovered that (1) condition-dependent change of mean cell-cycle time is strongly correlated with variability in cell-cycle durations; and (2) increase of the heterogeneity of generation times in a population may be the method to evolve to a higher population growth rate in a constant environment, which is partly parallel to our conclusions. In our case a higher variability of cell cycle times was observed in the population with shorter mean cell-cycle times (10% FBS, mean cell cycle time 21.6 h, MAD = 0.17, CV = 0.25; 15% FBS, mean cell cycle time 20.4 h, MAD = 0.21, CV = 0.30).

In the second paper [43], a method to predict histories of single cells in an exponentially growing population was proposed. Analysis revealed that physiological differences in sister cells have a significant impact on individual cell histories and their contribution to the overall population-growth.

### Cell proliferation and Wright-Fisher model

As stated previously, the models of cell proliferation and the models of genetic change in populations have been historically based on two apparently contradictory hypotheses, i.e. the unlimited branching process and a completely constant population size, respectively. By necessity, when it became clear that somatic mutations in proliferating eukaryotic cells are important for growth rates, the population constancy assumption in genetic models has been relaxed. A seminal paper concerns the Wright-Fisher coalescent under exponentially growing population [44], followed by a number of models developed for other growth patterns, such as the model in ref. [45], where linear growth has been considered. Polanski and Kimmel in ref. [22], developed computable expressions for a Wright-Fisher coalescent with arbitrary growth pattern (originally due to [46]). We analyzed the experimental and modelled cell pedigrees to determine patterns of inheritance of cell-cycle phase durations for aggregated G1 and S/G2/M phases, based on dynamic imaging of live NIH 3T3 cells. We developed estimates of the parameters of bifurcating autoregression model with lognormal distributions given observed cell-cycle phase durations, and fitted single-cell protein trajectories to the ODE model and found correlations among parameters between single cells (sib-sib, parent-progeny, and other). Parent-progeny and sib-sib correlations from the experimental data were well-reproduced by our modelling, as demonstrated by comprehensive comparisons. Results showed stronger inheritance of the S/G2/M duration compared to G0/G1. Using the model developed, we simulated its transient and long-term behavior and interpreted it in the terms of population genetics. Long-term simulations demonstrated the model had homeostatic properties. However, progeny of a single cell with a short interdivision time had a high probability of eventually dominating the population, due to heritability of cell-cycle phases. Analysis of model simulations showed that an effect similar to genetic drift was present in the model; however, it was amplified by family relationships among cells. This was manifested by reduction of the effective population size compared to the standard Wright-Fisher model of drift. Such patterns of epigenetic heritability in proliferating cells are important for understanding long-term trends of cell populations which are either required to provide influx of maturing cells (such as hematopoietic stem cells), or which relaxed controls and started proliferating uncontrollably (such as cancer cells).

Specifically, we investigated adherence of our simulations to the Wright-Fisher model. We found that after 300 h, population started from *N* ancestral cells consists of their descendants in random proportions similar as in the Wright-Fisher model with effective population size *K* much smaller than the census size (straight count of individuals). This is different from the previous studies fitting cell population drift using a Moran model (which may be considered as a version of Wright-Fisher), in which the effective population size was equal to the census size; cf. [47] and references therein.

In addition, we investigated the dependence of *K* on the correlations existing in our model. As depicted in Fig 5, *K* is largest for the case in which both parent-progeny and sib-sib correlations are close to 0. Also in this case, *K* is almost identical to that obtained from the harmonic mean of population sizes at different times, which is the textbook method for computing the effective population size for expanding populations ([29], Equ. (2.13); also see Methods, *Wright-Fisher model and the cell cycle model*). For numerical comparisons, see Table 2. These findings illustrate the importance of including the correlations in the model.

The most important conclusion is that in the presence of family relations, the estimated effective population size *K* is smaller than that obtained by using the harmonic-mean law. Using parent-progeny and sibling correlations estimated from data, we obtain *K* that is about 45% lower. As a consequence, drift acts in cell populations stronger than under the strict Wright-Fisher model with population growth, which increases the impact of random fluctuations in such populations (see the last section of Results). This seems to be of importance in two contexts, in which continued cell proliferation takes place. One of these is cancer growth, in which an initially small population expands and diversifies by somatic mutations but also epigenetic changes [48–50]. Genetic or epigenetic drift acts at the stage when the tumor is very small, but also in isolated secondary foci in some cancers. A very well-documented study of neutral evolution of this kind has been carried out for hepatocellular carcinoma [51]. The other context is healthy human hematopoiesis, in which a relatively small population (ca. 10,000 cells) of hematopoietic stem cells (HSC) proliferates throughout the lifetime, diversifying into a number of descendant lineages and producing about 10^9^ mature blood cells per day [52]. In the course of infections, the HSC become activated and if the incidents recur, their number and heterogeneity may permanently decrease [20], which also makes the healthy HSC less competitive if a malignant clone arises. Since HSC population is distributed among smaller bone-marrow neighborhoods called niches, drift is likely to act strongly in this population. Reduced *K* amplifies these effects. This may also be the case in development of other stem-cell types, such as in hippocampal neurogenesis. The role of heterogeneity in this system is becoming an intensive research focus [53]. One of the interesting phenomena is heterogeneity reduction with age, which hypothetically might be due to stem-cell population bottlenecks, which have been demonstrated using the branching process model by Li and co-workers [54].

## Methods

### Experimental procedures (Cell line)

Replication-defective, self-inactivating retroviral constructs were used for establishing NIH3T3 FUCCI-2A cell line as described in ref. [55].These cells stably express the Cdt1 and Geminin sequences coding for G1 and S/G2/M probes, fused to mKO2 and E2-Crimson fluorescent reporters, respectively. They are separated by a 2A sequence to allow post-translational cleavage and followed by a puromycin-resistance cassette for subsequent selection.

Before recording, cells were seeded at 7–10% confluence (10^5^ cells per well) in a 6-wells plate (Falcon), with white DMEM medium (high glucose) containing 1% Penicillin/Streptomycin, 10 mM HEPES and either 10% or 15% FBS. Cells were left undisturbed for 48 hours. For recording, cells were placed in a Zeiss Axiovert 200M microscope (Zeiss) with a 20X Ph objective. A culture chamber, temperature and CO2 controller (Pecon) were used to ensure constant suitable conditions for long-term recording of the cells. Images were recorded every 15 minutes for 72 hours, using a Coolsnap HQ/Andor Neo sCMOS camera. Cells were briefly illuminated with a FluoArc HBO lamp (Zeiss) at reduced intensity. Epifluorescence signals were recorded as follows: mKO2: 300 ms (filter cube: Ex 534/20 –Di 552 –Em 572/38), E2Crimson: 800 ms (filter cube: Ex 600/37—Di 650—LP 664).

### Model of cell cycle

The modelling paradigm we employ is based on the hypothesis that the timing of major events in the cell cycle is heritable in proliferating cells. This timing and its heritability are controlled by an intricate mechanism, which has been partly elucidated [56], but its details require more resolution than we can build into our model. Other processes, such as synthesis of FUCCI proteins, occur within this time. Another driving factor is unequal division of proteins among progeny cells. It has been demonstrated theoretically [12, 57] and confirmed by fitting models to data [4, 11], that models based on similar hypotheses exhibit homeostatic properties. This amounts to regulatory feedbacks acting in the model. However, none of these models has been based on data of such resolution as the present one.

#### Heritable cell cycle phase times

Residence times in progeny G1 and S/G2/M cell cycle phases, given the parents’ residence times, follow the bifurcating autoregression equations

$$\begin{array}{l} T_{G1}^{1}=m_{G1}+\theta_{G1}\left(T_{G1}-m_{G1}\right)+W_{G1}^{1}\\ T_{G1}^{2}=m_{G1}+\theta_{G1}\left(T_{G1}-m_{G1}\right)+W_{G1}^{2} \end{array}$$ (1)

$$\begin{array}{l} T_{SG2M}^{1}=m_{SG2M}+\theta_{SG2M}\left(T_{SG2M}-m_{SG2M}\right)+W_{SG2M}^{1}\\ T_{SG2M}^{2}=m_{SG2M}+\theta_{SG2M}\left(T_{SG2M}-m_{SG2M}\right)+W_{SG2M}^{2} \end{array}$$ (2)

where the superscript *i* = 1,2 denotes the number of progeny cell; $T_{G1}^{i},T_{SG2M}^{i}$ are G1 or S/G2/M phases length of progeny cell *i*, *T*_*G*1_, *T*_*SG*2*M*_ are G1 or S/G2/M phases length of the parent cell. Constants *m*_*G*1_,*m*_*SG*2*M*_ are equal to mean G1 or S/G2/M cell-cycle phase durations plus a corrective term required to ensure long-term equilibrium (see further on); *θ*_*G*1_,*θ*_*SG*2*M*_ are fractions of the durations of the G1 or S/G2/M phases inherited from the parent; and the noise terms $W_{G1}^{i},W_{SG2M}^{i}$ are random vectors from exchangeable bivariate lognormal distributions (component-wise transformation by exponential function of bivariate normal with equal means and variances). Cell-cycle time is the sum of residence times in G1 and SG2M

$$T_{C}=T_{G1}{+T}_{SG2M}.$$

This model deviates from the original bifurcating autoregression of Cowan and Staudte which assumes bivariate normal distribution. The modification accommodates the nonnegativity and positive skewness of the distributions of cell-cycle phase durations.

#### Unequal division of FUCCI proteins between progeny cells

We used the following stochastic multiplicative laws

$$\begin{array}{l} C^{1}=X_{C}C,C^{2}=(1-X_{C})C,\\ G^{1}=X_{G}G,G^{2}=(1-X_{G})G, \end{array}$$

where *C*,*C*^1^,*C*^2^,*G*,*G*^1^,*G*^2^ are the parent, progeny 1 and progeny 2 amounts of Cdt1 and Geminin, respectively, and independent random variables *X*_*C*_ *X*_*G*_ have beta distributions B(*α*,*β*), such that E(*X*_*C*_) = E(*X*_*G*_) = 0.5 and cv(*X*_*C*_) = cv(*X*_*G*_) = *cv*. Value of *cv* has been found based on data as explained later. This results in *α* = *β* = (*cv*^−2^−1)/2, considering that with *α* = *β*, *cv* = (2*α*+1)^−0.5^. Beta distribution is used because it constitutes a flexible family of random variables restricted to the unit interval of the real line.

#### FUCCI Protein synthesis (coefficients based on linear regression, using initial concentrations of proteins and cell cycle phase lengths)

In normal cells, Cdt1 and its inhibitor Geminin play a role in cell-cycle regulation (S13 Fig), and one can indirectly affect the dynamics of the other. However, our measurements are based on fluorescence of markers containing only fragments of Cdt1 and Geminin. As a consequence, the markers are produced and degraded in proportion to the “full-scale” Cdt1 and Geminin, but they do not interact with other proteins [14, 15]. Single-cell dynamics of the concentrations of the two markers are described using the following equations (as earlier on, *C* variable for Cdt1, *G* for Geminin):

$$\begin{array}{l} \dot{C}\left(t\right)=p_{C}-d_{C}C\left(t\right)-d_{C-SG2M}\left(t\right)C\left(t\right)\\ \dot{G}\left(t\right)=p_{G}-d_{G}G\left(t\right)-d_{G-G1}\left(t\right)G\left(t\right) \end{array}$$ (3)

where following variables are used to describe the two phases:

*p*_*C*_—production rate of Cdt1 protein, constant in time,

*p*_*G*_—production rate of Geminin protein, constant in time,

*d*_*C*_–background degradation rate of Cdt1 protein, constant in time,

*d*_*G*_–background degradation rate of Geminin protein, constant in time,

*d*_*C*,*SG*2*M*_(*t*) = *a*(*t*−*T*_*G*1_)1(*t*−*T*_*G*1_)—degradation rate of Cdt1 protein, linearly increasing in time during the G/S2/M phases of the cell cycle and equal to 0 otherwise,

*d*_*G*,*G*1_(*t*) = *b*(*T*_*G*1_−*t*)1(*T*_*G*1_−*t*)—degradation rate of Geminin protein, linearly decreasing in time during the G1 phase of the cell cycle and equal to 0 otherwise.

The phase-dependent degradation rates were assumed to provide a simplified description of a large number of regulatory processes inside cells, such as formation of complexes, their activation and deactivation. As it is known, the cell cycle is controlled by ubiquitin (Ub)-mediated proteolysis. Activity of two complexes: APC^Cdh1^ and SCF^Skp2^ oscillate reciprocally during the cell cycle. Cdt1 and Geminin are direct substrates of these complexes [14].

For purposes of long-term simulations, the protein production rates of simulated cells are drawn from linear regression equations, following the assumptions that:

1. *p*_*C*_ depends linearly on *T*_*G*1_ and *C*_0_, and
2. *p*_*G*_ depends linearly on *T*_*SG*2*M*_ and *G*_0_.

Mathematically,

$$\begin{array}{l} p_{C}=a_{G1}+r_{1,G1}T_{G1}+r_{2,G1}C_{0}+\varepsilon_{G1}\\ p_{G}=a_{SG2M}+r_{1,SG2M}T_{SG2M}+r_{2,SG2M}G_{0}+\varepsilon_{SG2M} \end{array}$$ (4)

where *C*_0_ and *G*_0_ are the amounts of the Cdt1 and Geminin, respectively, at the beginning of the cell cycle, and *ε*_*G*1_ and *ε*_*SG*2*M*_ are random error terms. Random numbers are drawn from two-dimensional normal distribution to reproduce correlation between siblings: $\rho_{p_{C}}=0.66,\rho_{p_{G}}=0.54$. Parameters were estimated using linear regression from single-cell data. There is generally high agreement among parameters simulated in this manner, and the single-cell data is depicted in Fig 2E.

### Estimation of parameters

#### Estimation of the bifurcating autoregression parameters

We accomplish this task using the method of moments; in this case, the first and second moments. For clarity, we omit the subscripts corresponding to cell cycle phases G1 and SG2M. It is understood that estimation is carried out separately for G1 and SG2M.

The noise terms in Eqs 1 and 2 are distributed according to the version of exchangeable bivariate lognormal distribution, which arises when coordinates of an exchangeable bivariate normal vector *X* are transformed by the exponential function. Suppose *X* = (*X*^1^,*X*^2^)~*MVN*(*μ*,Σ), where *μ* = (*μ*,*μ*) is the vector of expected values and $\Sigma =\sigma^{2}\left(\begin{array}{cc} 1 & \rho \\ \rho & 1 \end{array}\right)$ is the variance-covariance matrix of rv *X*. The resulting bivariate lognormal rv *W* = (*W*^1^,*W*^2^) = (exp(*X*^1^),exp(*X*^2^)) has a joint density function that can be derived in a standard way, and the first- and second-order moments have the form

$$E(W^{i})=e^{\mu +\sigma^{2}/2},Var(W^{i})=e^{2\mu }e^{\sigma^{2}}(e^{\sigma^{2}}-1),Cov(W^{1},W^{2})=e^{2\mu }e^{\sigma^{2}}(e^{{\rho \sigma }^{2}}-1)$$

hence the correlation coefficient is equal to

$$\rho_{W^{1},W^{2}}=(e^{{\rho \sigma }^{2}}-1)/(e^{\sigma^{2}}-1)$$

We assume now that

1. Equilibrium distribution arises in the long-time limit in the model, so that times *T* and *T*_*i*_ are all identically distributed. This is the consequence of the general theorem on time series models, see eg. the monograph ([58], Theorem 4.3.1., p. 304).
2. Cell measurements represent such equilibrium,
3. In the bifurcating autoregression right-hand sides, *W*^*i*^ and *T*^*i*^ are independent rv’s.

This allows deriving the following moment equations

$$E(T^{i})-m=\theta (E(T)-m)+E(W^{i})$$

$$Var(T^{i})=\theta^{2}Var(T)+Var(W^{i})$$

$$Cov(T^{1},T^{2})=\theta^{2}Var(T)+Cov(W^{1},W^{2})$$

$$Cov(T^{i},T)=\theta Var(T)$$

Remembering that under equilibrium, E(*T*^*i*^) = E(*T*) and Var(*T*^*i*^) = Var(*T*), we obtain

$$E(T)=m+\frac{e^{\mu +\sigma^{2}/2}}{1-\theta }$$ (5)

$$Var(T)=\frac{e^{2\mu }e^{\sigma^{2}}(e^{\sigma^{2}}-1)}{1-\theta^{2}}$$ (6)

$$\rho_{T_{1},T_{2}}=\theta^{2}+(1-\theta^{2})\frac{e^{{\rho \sigma }^{2}}-1}{e^{\sigma^{2}}-1}$$ (7)

$$\rho_{T_{i},T}=\theta$$ (8)

and combining expressions for parent-progeny and progeny-progeny covariances, we obtain cousin-cousin correlation coefficient

$$\rho_{T_{1i},T_{2i}}=\rho_{T_{1},T_{2}}\theta$$ (9)

We can now solve these equations for the four unknown parameters *θ*,*σ*^2^,*μ*, and *m*, assuming a value for parameter *ρ*. Following this, we can substitute for the moments E(*T*) and Var(*T*) and correlation coefficients $\rho_{T_{1},T_{2}}$ and $\rho_{T_{i},T}$ their sample-based estimates $\hat{E(T)},\hat{Var(T)},\hat{\rho_{T_{1},T_{2}}}$ and $\hat{\rho_{T_{i},T}}$, and obtain the method-of-moments estimates of the unknown parameters, given assumed *ρ*. Subsequently, we can adjust *ρ* so that the distributions of residence times *T* fit data best.

We first note that by Eq (8)

$$\hat{\theta }=\hat{\rho_{T_{i},T}}$$

Now, we use Eq (7) in the form

$$\frac{e^{{\rho \sigma }^{2}}-1}{e^{\sigma^{2}}-1}=\frac{\hat{\rho_{T_{1},T_{2}}}-{\hat{\theta }}^{2}}{1-{\hat{\theta }}^{2}}=\hat{A}\in (0,1)$$

to write a transcendental equation for $x=e^{\sigma^{2}}(>1)$

$$x^{\rho }-1=\hat{A}(x-1).$$

Since ${\frac{d{(x}^{\rho })}{dx}|}_{x=1}=\rho$, therefore, for *ρ*>*A*, there exists *x* = *x*_0_>1, which satisfies this equation. This provides

$$\hat{\sigma^{2}}=ln(x_{0}).$$

The remaining two estimates are obtained by simple inversion of Eqs (5) and (6)

$$\hat{\mu }=\frac{1}{2}\ln\frac{\hat{Var(T)}(1-{\hat{\theta }}^{2})}{e^{\hat{\sigma^{2}}}(e^{\hat{\sigma^{2}}}-1)}$$

$$\hat{m}=\hat{E(T)}-\frac{e^{\hat{\mu }+\hat{\sigma^{2}}/2}}{1-\hat{\theta }}$$

#### Estimation of the parameters of the distribution of unequal division of FUCCI proteins between progeny cells

Value of the coefficient of variation *cv* of the random variable *X* in the multiplicative model of unequal division has been found, based on comparison with simulations. Direct measurement of the extent to which the proteins are unequally distributed between progeny cells is difficult due to degradation of the FUCCI markers in late M phase. We performed simulations to calibrate the impact of spread of this distribution on dynamics of protein levels in the progeny cells. We assumed the *cv* = 0.13 the same as that in ref. [4], calculated for mammalian cells. Varying *cv* did not alter the outcome in a substantial manner, therefore *cv* = 0.13 has been kept, which corresponds to parameter values *α* = *β* = 29.3 of the beta distribution model.

#### Estimation of FUCCI protein synthesis ODE model

It is assumed that dynamics of protein synthesis can be described using Eq (3). Experimental trajectories of protein levels in individual cells have been used to find individual production rates, with degradation rates assumed the same in all cells. These and other parameters such as ***C***_***G*1**_, the amount of Cdt1 protein at the end of G1 phase, and ***G***_***end***_, the amount of Geminin protein at the end of the cell cycle, were fitted iteratively using Eq (4) and the following heuristic relationships

$$p_{C}=\frac{C_{G1}}{T_{G1}}\vartheta_{C},$$

$$p_{G}=\frac{G_{end}}{T_{SG2M}}\vartheta_{G}.$$

Empirical parameters *ϑ*_*C*_ and *ϑ*_*G*_ link protein production rates with the ratios of the protein peak values and the respective durations of cell-cycle phases. This amounts to linear interpolation of the arcs of exponential functions.

Cell-cycle phase durations for all cells have been estimated using an original technique (S14 Fig) based on information about division times of the cells and their parents and fluorescence levels of FUCCI proteins. It allows detecting cell cycle and phases endpoints, but also verifying if the trajectory is of appropriate quality and can be subject of further analysis. Briefly, it includes the following steps (S14 Fig): (i) determination of the level of noise and of the parameter values for smoothing, (ii) computing of numerical derivatives of the trajectories of Cdt1, Geminin protein levels, (iii) detection of local minima of differentiated data to identify division times, and (iv) detection of Cdt1 protein maxima, the timing of which provides the estimated moment of transition from G1 to S phase of cell cycle (in this step we analyze only the interval of Cdt1 protein trajectory located between division times). The maxima of the expression of the Cdt1 protein, just before a rapid drop, are detectable in 99.7% of cells. In general, problems at each step of analysis were mostly caused by aberrant protein trajectories in some cells, such as the protein level not changing in time, or the almost constant noise level being extremely high, which made it impossible to retrieve the protein dynamics signal.

Application of the procedure resulted in the following estimates of parameters that are identical for all cells, *d*_*C*_ = 0.08, *d*_*C*−*SG*2*M*_ = 0.088, *ϑ*_*C*_ = 6, *d*_*G*_ = 0.0,*d*_*G*−*G*1_ = 0.124, *ϑ* = 4.

### Wright-Fisher model and the cell cycle model

#### Mathematical framework

In the canonical Wright-Fisher model of population genetics, time is measured in discrete units (generations), and the number of individuals in each generation is constant and equal to ***N***. Individuals of generation ***t*** contribute to the ***t***+**1**-st generation by multinomial sampling. In mathematical terms, let us denote ***X***_***i***_ the number of the ***t***+**1**-st generation descendants of the ***i***-th individual of the ***t***-th generation. Then ***X*** = (***X***_**1**_,***X***_**2**_,…,***X***_***N***_)~**MN**(***N***^**−1**^,…,***N***^**−1**^;***N***), which, among other things, implies that ***X***_***i***_~**Binom**(***N***^**−1**^;***N***). The cell proliferation process we consider runs in continuous time and the cell count changes with time. Let us consider a population started at time ***t*** = **0** by ***N*** cells. Let us consider a time interval of length **Δ*t*** and denote ***Z***_***i***_(**Δ*t***) = #{**progeny of *i* at time Δ*t***}. If the distribution of vector ***X*** = [***Z***(**Δ*t***)/|***Z***(**Δ*t***)|] ∙ ***K*** approximates a multinomial distribution, then we may consider the cell proliferation process as approximately conforming to a Wright-Fisher model with effective population size ***K*** (which depends on **Δ*t***). Technically, ***X*** is also a discrete random vector, although not integer-valued. However, comparisons based on distribution tails are valid for any random variables.

The rationale for our approach follows from practice well established in population genetics, of approximating real-life populations by populations obeying Wright-Fisher model with “effective population size” that is different from the actual “census population size”. For example, if the population size has been varying in the past, the effective population size is frequently approximated as a harmonic mean of the census population sizes in successive past generations ([29], Equ. (2.13)).

In our notation, this harmonic-mean estimate of *K* has the form of

$$K={\left\{{\Delta t}^{-1}\int_{0}^{\Delta t}{\left[\sum_{i=0}^{N}Z_{i}(\tau )\right]}^{-1}d\tau \right\}}^{-1}$$

Assuming that *Z*_*i*_(*t*) are equal to their expected values, i.e. that *Z*_*i*_(*t*) = *Z*_*i*_(0)*e*^*αt*^, where *α* is a growth rate estimated from simulated trajectories (for example, by representing them in semi-logarithmic scale and estimating the slope *α* of the graph using linear regression), we obtain

$$K=\frac{N_{0}N_{T}}{N_{T}-N_{0}}ln(\frac{N_{T}}{N_{0}})$$

We will see that these two estimates provide almost identical values of *K*.

However, it has been established at least since the well-known publication of Rogers and Harpending (1992) [59] that the Wright-Fisher model does not generally apply with a good approximation when population undergoes demographic change. Nevertheless, we demonstrate here that the approximation is acceptable at least in the terms of marginal distributions (see Results). Explanation of possible reasons for this is postponed to Discussion.

#### Statistical considerations

To obtain an informal test, we consider the marginal distributions, which under multinomial joint distributions are binomial, and the second moments. As stated, we expect that

$$X=(X_{1},X_{2},\ldots ,X_{N})∼MN(N^{-1},\ldots ,N^{-1};K)\Rightarrow X_{i}∼Binom(N^{-1};K)$$

Upon rescaling by the effective population size *K* so that *Y* = *X*/*K*,

$$E(Y_{i})=N^{-1},Var(Y_{i})=(N-1)N^{-2}K^{-1},Cov(Y_{i},Y_{j})=-N^{-2}K^{-1},Var(Y_{i})-Cov(Y_{i},Y_{j})=N^{-1}K^{-1},i\neq j$$

From simulations, we obtain *M* sample trajectories generated by our model, which can be rescaled by the estimated effective population size. This latter can be accomplished by fitting the empirical distributions of *X*_*i*_ to the Binom(*N*^−1^;*K*) distribution. As a result, we obtain a sample in the form of a table: ${\left\{Y_{i}^{\left(m\right)}\right\}}_{i=1,\ldots ,N}^{m=1,\ldots ,M}$. We consider two statistics $S_{r}^{2}={\left(NM\right)}^{-1}\sum_{i,m}{\left(Y_{i}^{\left(m\right)}-N^{-1}\right)}^{2}$ and $S_{rc}^{2}={\left(NM\right)}^{-1}\sum_{i,m}{\left(Y_{i}^{\left(m\right)}-{\bar{Y}}_{i}\right)}^{2}$, where ${\bar{Y}}_{i}=N^{-1}\sum_{m}Y_{i}^{\left(m\right)}$. Then, remembering that the entries of *Y*^(*m*)^ are not independent, we obtain

$$E(S_{r}^{2})={\left(KN\right)}^{-1}=Var(Y_{i})-Cov(Y_{i},Y_{j}),E(S_{c}^{2})={\left(1-N^{-1})(KN\right)}^{-1}=Var(Y_{i}).$$

Using these two expressions, we can estimate by the method of moments, the variance and covariance under exchangeability assumption. The results are depicted in Table 1.

## Supporting information

S1 Fig Comparison of repetitions of the same experiment (15% FBS). Boxplots represent cell-cycle length. During observation, randomly selected area is recorded during 72 hours. Movies differ with respect to initial number of cells and their location.
(TIF)

S2 Fig Comparison of repeats of the same experiment (15% FBS). The correlation between: 1) cell cycle and G1 phase, 2) cell cycle and S/G2/M phases and 3) G1 and S/G2/M phases, 4) sample sizes.
(TIF)

S3 Fig Family relations and differences between experiments. (A) Correlations between family members based on experimental data. Estimation of standard deviations is described in detail in Methods. (B) Correlations of cell cycle length between family members for two selected movies. 75% of information about cousins came from these movies. Strong correlation between cousins is specific for case 2. (C) Verification of the hypothesis that cell-cycle duration depends on the birth date of the cell. Cells’ birth dates rounded to the nearest multiplicity of 2 hours are presented as boxplots to address the hypothesis. (D) Cross-plot of cells’ birth date and the cell-cycle length for cells from two selected movies. (E) Individual traces for cousins. Each color denotes one pair of cousins; a large dot indicates position of cells at the beginning of the cell cycle; information about cell-cycle duration is also included.
(TIF)

S4 Fig Relationships between durations of the cell cycle and the G1 and S/G2/M phases. (A) Experimental data. Linear relationship between the total division time and the duration of phases. Solid black lines show the fitted linear relations of the form *y* = (*slope*)×*x*. (B) Linear relations presented in the histograms, where the distribution of proportionality is shown. In 80% of samples, G1 phase occupies 35–55% of the cell cycle. (C) Cross-plot of the times of G1 and S/G2/M cell-cycle phases. *Blue*, “normal” cells; *green*, extended cell- cycle-length cells with longer S/G2/M phases; and *red*, extended cell-cycle-length cells with longer G1 phase. (D) Gaussian mixture model distinguishing the “normal” from extended cell-cycle-length cells, combined with the EM (Expectation Maximization) algorithm, to estimate the threshold (22 h) for separation of cells into two groups. (E) Correlation between phases and cell-cycle lengths for family members.
(TIF)

S5 Fig Correlation matrices represent changes in protein dynamics in related and unrelated cells. X and Y axes represent fractions (0 to 1) of cell cycle progressed, with the gaps between measurements normalized to 0.01. Correlations between protein expressions at each time of cell cycle are found using corresponding coordinates. Correlation matrices can help finding parts of the cell cycle with similar dynamics, as it is shown in the diagonal area of the progeny-progeny matrix.
(TIF)

S6 Fig Time trajectories of Cdt1 and Geminin levels in single cells, and mean and median trajectories. Two cases: (A) Movies 41–48–472 measurements and (B) Movie 49–177 measurements.
(TIF)

S7 Fig Populations with different growth factors concentrations. (A) Comparison of cell cycle, G1 phase and combined S/G2/M phases durations for two serum (FBS) concentrations. Calculations were performed based on 105 and 642 measurements for 10% and 15% of FBS, respectively. Histograms were normalized, the height of each bar is equal to the probability of selecting an observation within the corresponding bin interval, and the height of all of the bars sums up to 1. All distributions have characteristic lognormal-like shape; additional information about medians are included directly on the plots. Lower dose of serum causes extension of G1 and S/G2/M phases and as a consequence of the whole cell-cycle length. Changes in the lengths of the cell cycle and of the S/G2/M phases are statistically significant (Wilcoxon rank sum test). (B) Pearson rank correlations between lengths of the phases and the cell cycle. Standard deviations were calculated using Monte Carlo cross validation and 10,000 iterations. (C) Comparison between protein dynamics. Each line denotes one cell, black solid line is mean trace, black dashed line is median trace. The division moments were selected using procedure described in Methods section. (D) G1 phase takes proportionally less time under 10% FBS (Wilcoxon rank sum test), as it is shown in the boxplot (105 samples for 10% FBS, and the same number of randomly selected samples for 15% FBS). (E) Survival function mapping division events onto time, based on cells that divided at least twice during the experiment, so the time of birth and death could be estimated. Kaplan-Meier curve maps division events onto time, including cells which divided only once, so that either their birth or death are not known. Significant difference between 10% FBS and 15% FBS is observed only when incomplete cell cycles are included.
(TIF)

S8 Fig Results of long-term behavior predicted by the model. (A) Histograms of cell-cycle lengths for a single ancestor cell and its progeny. After each of 4,000 divisions along a single line of descent, one randomly chosen progeny was used for analysis. *Blue* and *red* color represent cases with low (13.6 h) and high (61.3 h) initial cell-cycle length, respectively. The medians in both cases are similar (21.9 h and 21.8 h). (B) The scatter plot of initial cell-cycle length and median cell-cycle length after 400 generations. No correlation is observed is significant statistically (*ρ* = -0.04). (C) Heat maps representing changes in cell-cycle durations in next generations. Three colors represent different cell-cycle lengths: *blue* for measurements below the first quartile; *red* for measurements above third quartile, and *green* for measurements within the interquartile range. (D) Histograms of cell-cycle lengths for a population started from a single ancestor at 200 h of observation. *Blue* and *red* colors represent cases with low (13.6 h) and high (61.3 h) initial cell cycle length, respectively. (E) Scatter plot of initial cell-cycle length and population size after 200 h. Strong negative correlation is observed (*ρ* = -0.65). Growth curves for two extreme cases. *Blue* and *red* colors represent cases with low (13.6 h) and high (61.3 h) initial cell-cycle length (respectively). (F) Descendants of ancestor cells are identified and counted. Growth curves show differences between two cell populations.
(TIF)

S9 Fig Cell-cycle duration for across several generations. (A, B) Ten extreme cases presented in the form of chart, where x axis represents generation number, y axis cell-cycle length. (C, D) Fifty extreme cases presented in the form of a heat map, where x axis represents generation number, y axis represent single-cell lineage and color denotes cell-cycle length.
(TIF)

S10 Fig Scatter plots for cell-cycle length difference for pair of cousins and their physical distance. (TIF)

S11 Fig Detailed scatterplots of experimental and simulated data for model parameters. (PDF)

S12 Fig An example of “noisy” measurement. Phase portraits for case where qualitative pattern is different than in majority of cells, it is caused by high noise level.
(TIF)

S13 Fig Interaction between functional FUCCI proteins. Cdt1 and its inhibitor Geminin are important regulators of replication licensing [60]. In normal cells, a critical balance between these two proteins ensures that firing of each origin along the genome will take place only once per cell cycle. In our case we measure expression of dysfunctional proteins, but regulated in the same way as original ones. Source: [61].
(TIF)

S14 Fig The second method of estimation of the cell-cycle endpoints. It includes several steps: (1–2) identification of the level of noise and determination of the appropriate parameter values for smoothing, (local regression using weighted linear least squares and a 2nd degree polynomial model); (3) numerical differentiation of Geminin protein levels; (4) detection of local minima of differentiated data to identify division moments, and (5) detection of Cdt1 protein maxima, the timing of which provides the estimated moment of transition from G1 to S phase of cell cycle (in this step we analyze only fragment of Cdt1 protein dynamic located between division moments).
(TIF)

S1 Data S_Data_15%_FBS_All_Cells. Measured intensities for Cdt1 and Geminin extracted from tracking (15% FBS).
(XLSX)

S1 Movie Changes of Cdt1 and Geminin protein across the cell cycle. Black and blue dots represent experimental and simulation data, respectively.
(AVI)

S1 Text Supplement-Mura-Feillet. The file contains additional results, discussion, description of methods and references.
(DOCX)
