# Supplementary figures and images for: Mathematical modelling reveals unexpected inheritance and variability patterns of cell cycle parameters in mammalian cells
Source: PLoS Comput Biol. 2019 Jun 3;15(6):e1007054. doi: 10.1371/journal.pcbi.1007054 (PMC6564046; doi:10.1371/journal.pcbi.1007054)

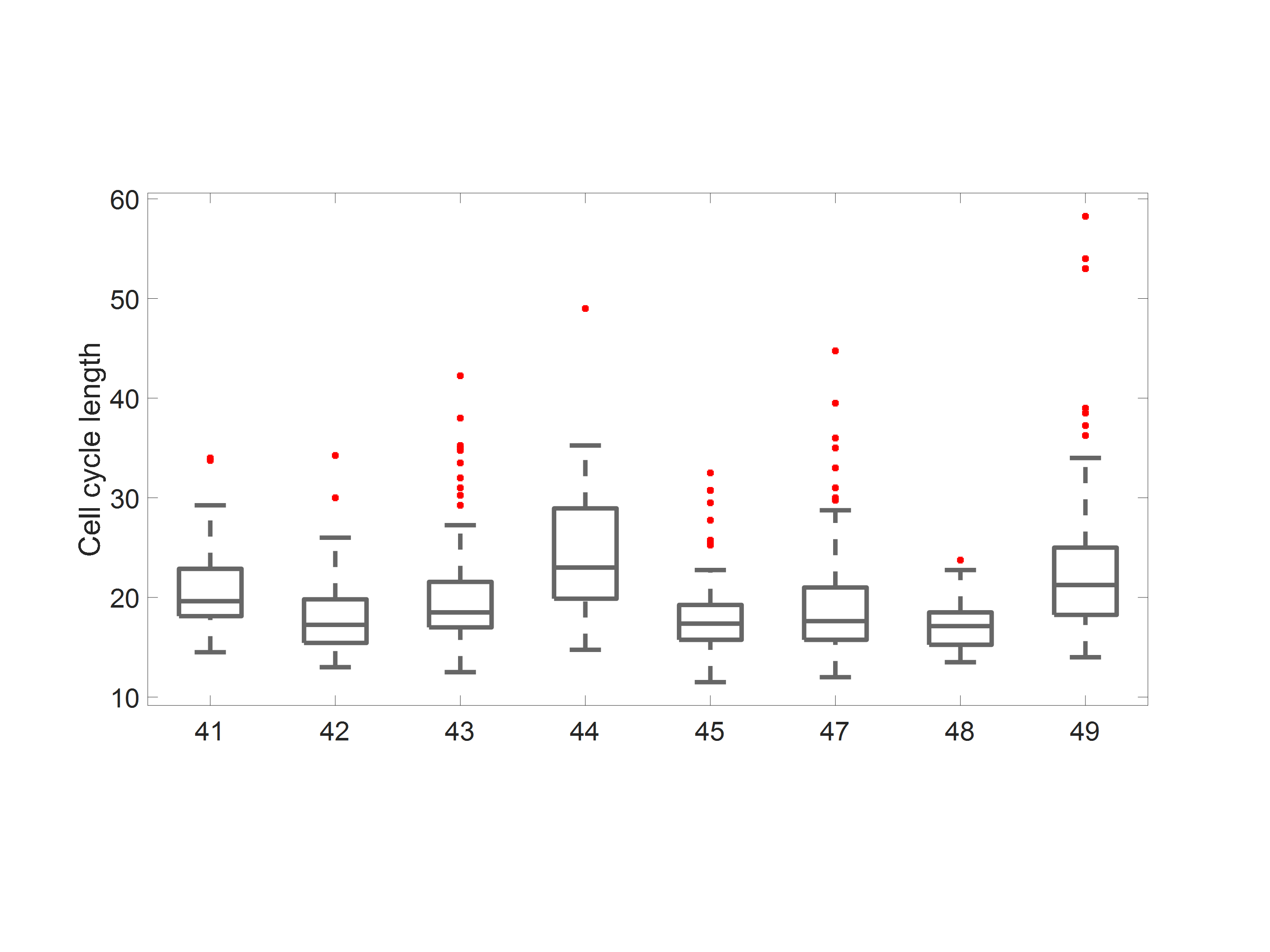

Supplement: S1 Fig — Boxplots represent cell-cycle length. During observation, randomly selected area is recorded during 72 hours. Movies differ with respect to initial number of cells and their location. (TIF) [file pcbi.1007054.s001.TIF]

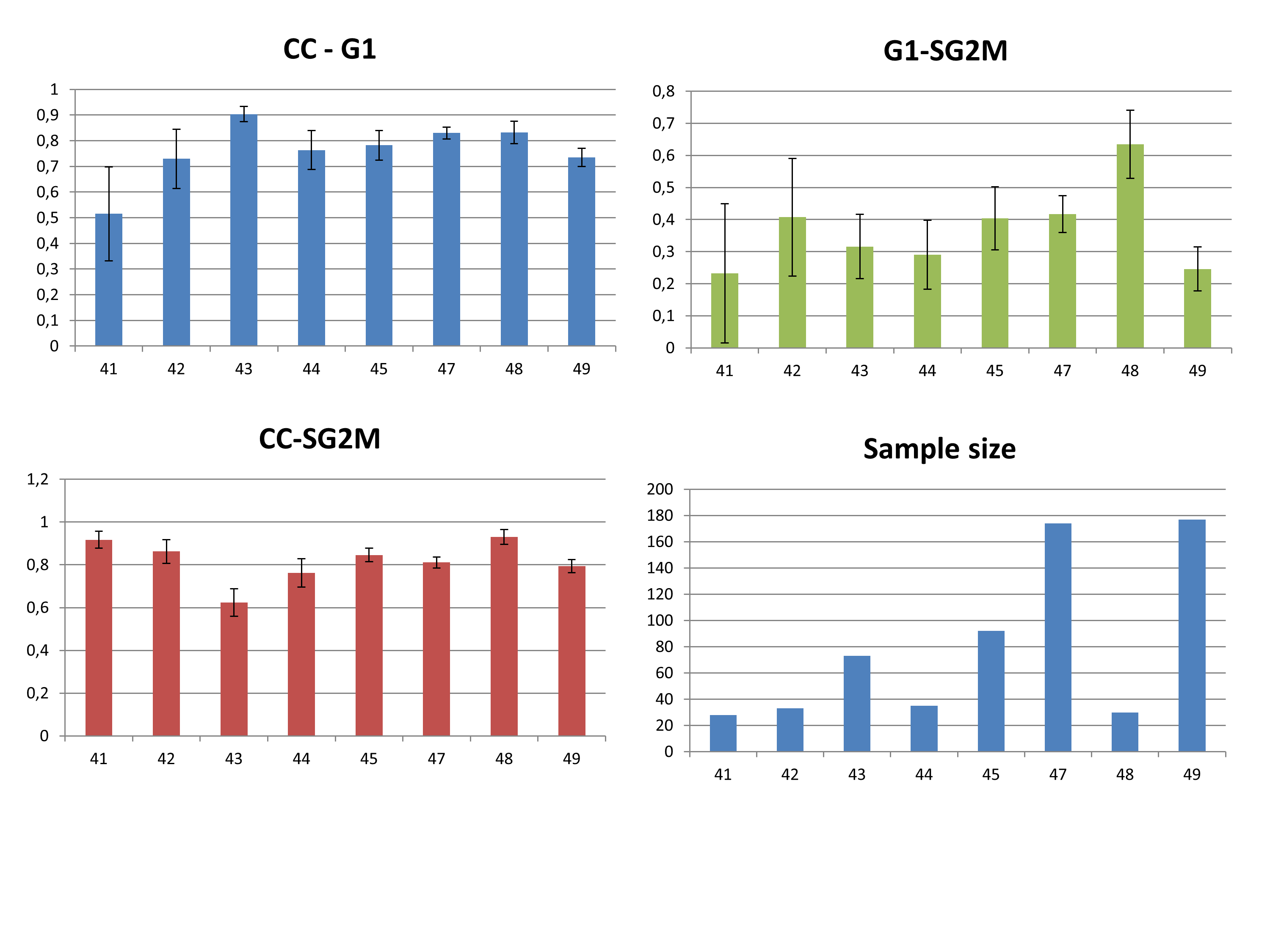

Supplement: S2 Fig — The correlation between: 1) cell cycle and G1 phase, 2) cell cycle and S/G2/M phases and 3) G1 and S/G2/M phases, 4) sample sizes. (TIF) [file pcbi.1007054.s002.TIF]

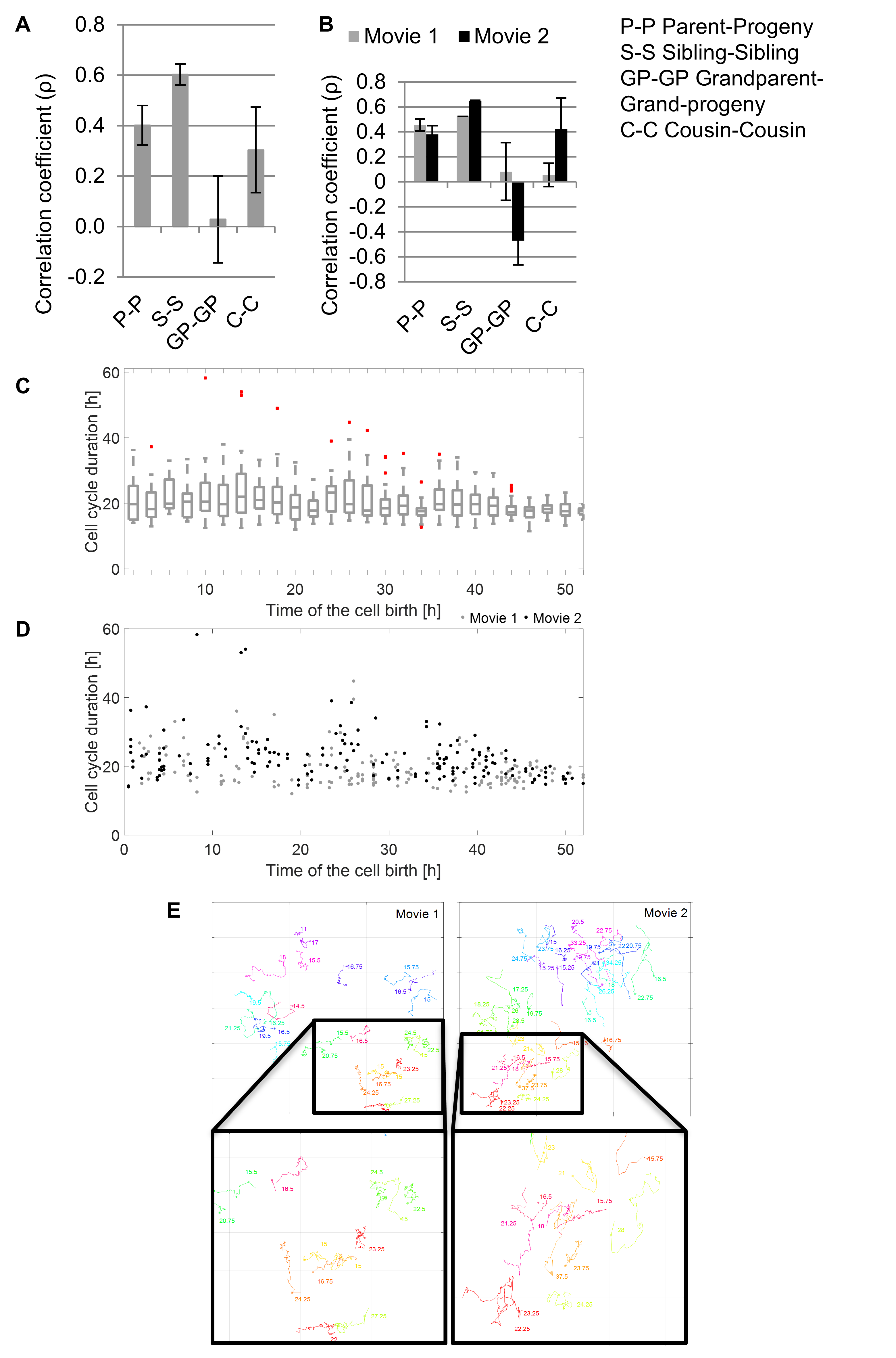

Supplement: S3 Fig — (A) Correlations between family members based on experimental data. Estimation of standard deviations is described in detail in Methods. (B) Correlations of cell cycle length between family members for two selected movies. 75% of information about cousins came from these movies. Strong correlation between cousins is specific for case 2. (C) Verification of the hypothesis that cell-cycle duration depends on the birth date of the cell. Cells’ birth dates rounded to the nearest multiplicity of 2 hours are presented as boxplots to address the hypothesis. (D) Cross-plot of cells’ birth date and the cell-cycle length for cells from two selected movies. (E) Individual traces for cousins. Each color denotes one pair of cousins; a large dot indicates position of cells at the beginning of the cell cycle; information about cell-cycle duration is also included. (TIF) [file pcbi.1007054.s003.tif]

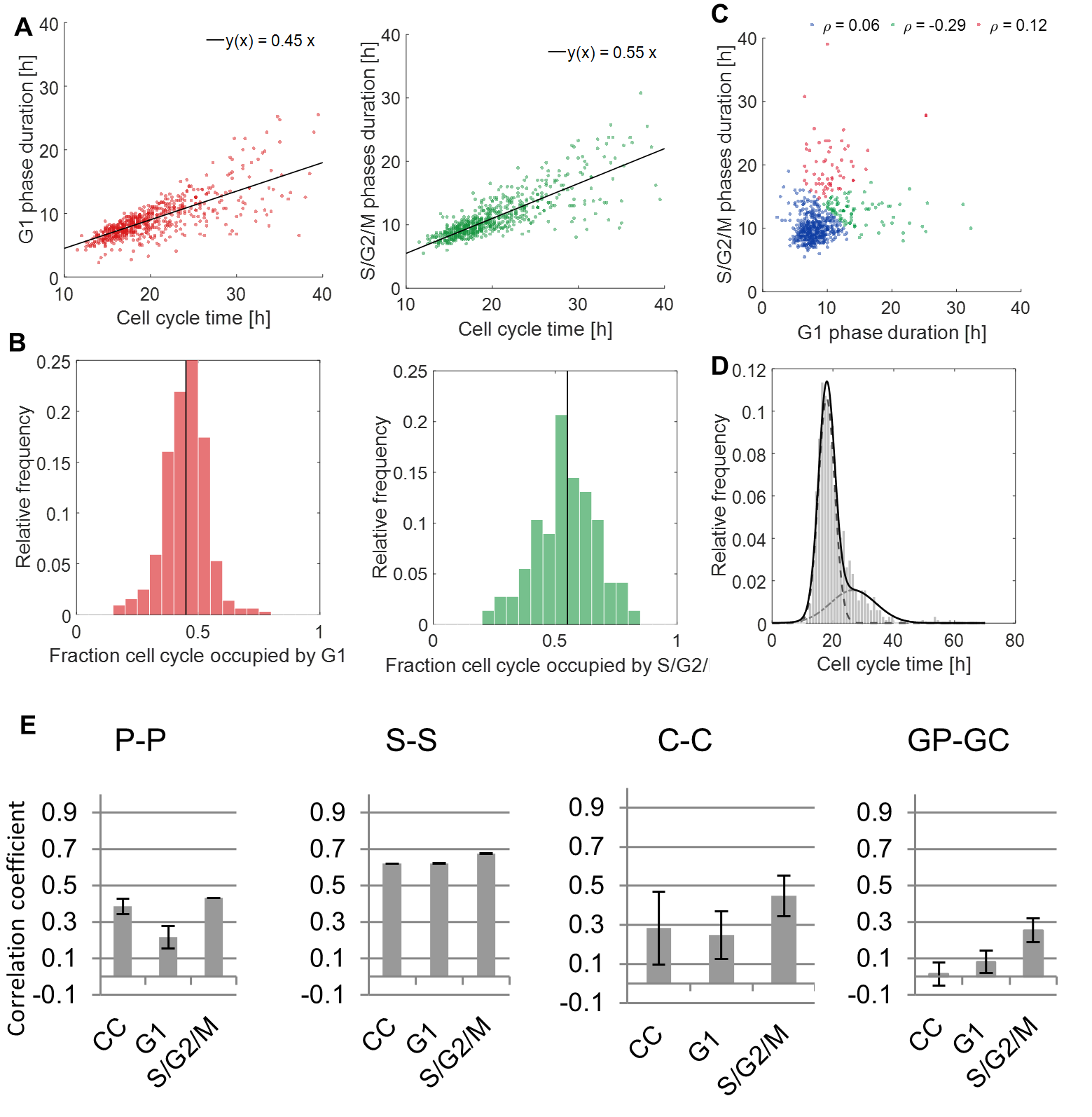

Supplement: S4 Fig — (A) Experimental data. Linear relationship between the total division time and the duration of phases. Solid black lines show the fitted linear relations of the form y = (slope)×x. (B) Linear relations presented in the histograms, where the distribution of proportionality is shown. In 80% of samples, G1 phase occupies 35–55% of the cell cycle. (C) Cross-plot of the times of G1 and S/G2/M cell-cycle phases. Blue, “normal” cells; green, extended cell- cycle-length cells with longer S/G2/M phases; and red, extended cell-cycle-length cells with longer G1 phase. (D) Gaussian mixture model distinguishing the “normal” from extended cell-cycle-length cells, combined with the EM (Expectation Maximization) algorithm, to estimate the threshold (22 h) for separation of cells into two groups. (E) Correlation between phases and cell-cycle lengths for family members. (TIF) [file pcbi.1007054.s004.tif]

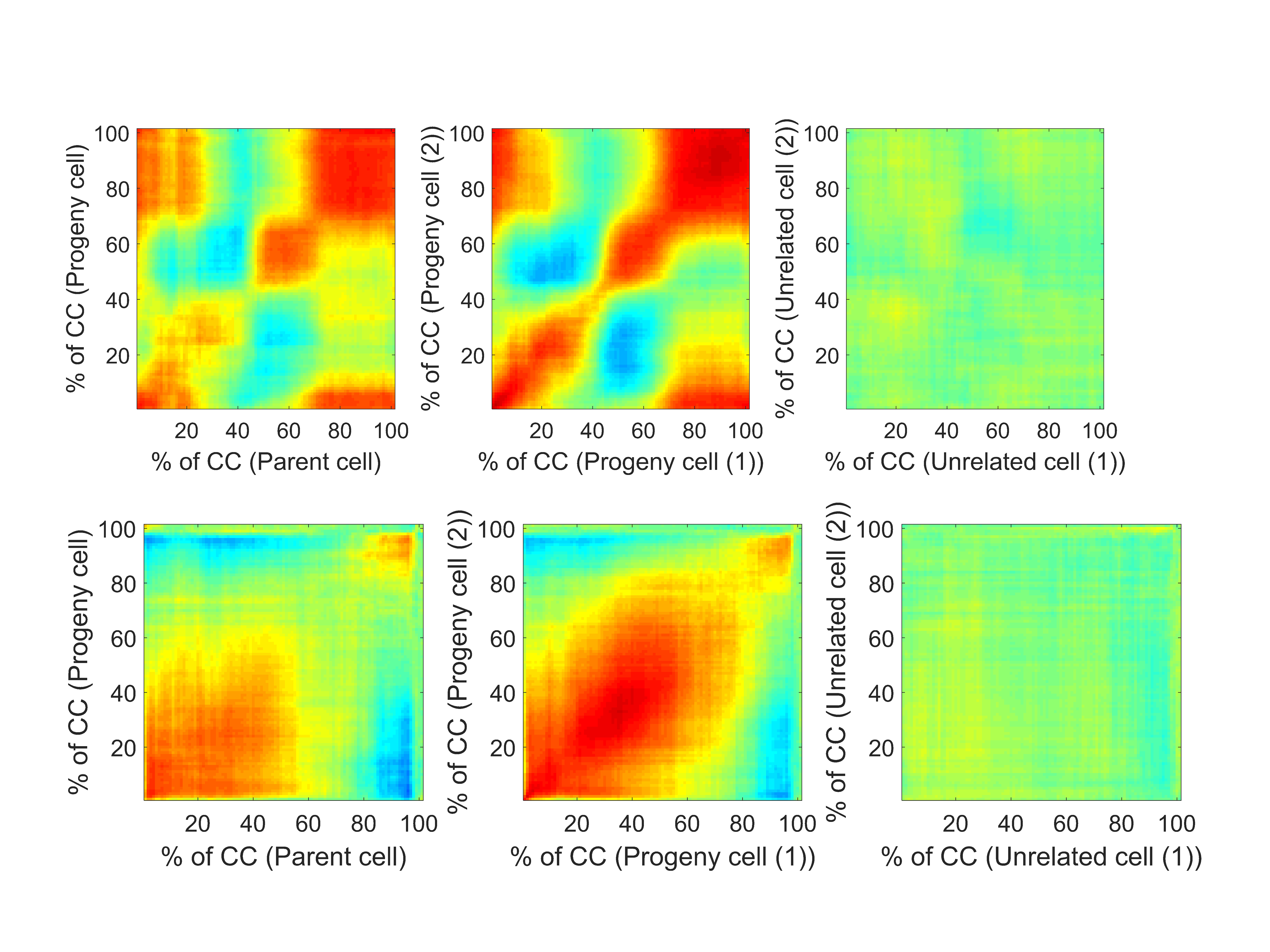

Supplement: S5 Fig — X and Y axes represent fractions (0 to 1) of cell cycle progressed, with the gaps between measurements normalized to 0.01. Correlations between protein expressions at each time of cell cycle are found using corresponding coordinates. Correlation matrices can help finding parts of the cell cycle with similar dynamics, as it is shown in the diagonal area of the progeny-progeny matrix. (TIF) [file pcbi.1007054.s005.tif]

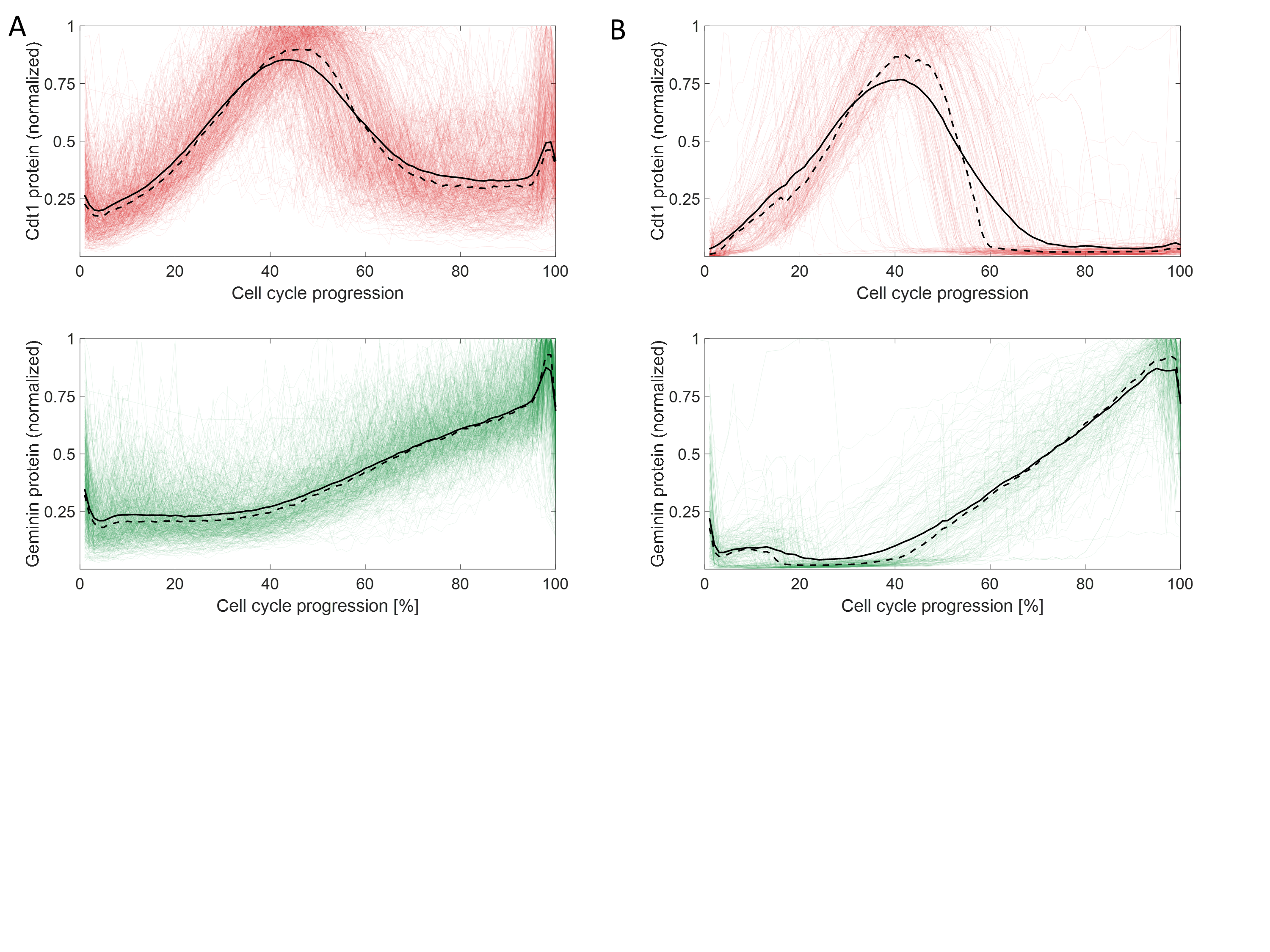

Supplement: S6 Fig — Two cases: (A) Movies 41–48–472 measurements and (B) Movie 49–177 measurements. (TIF) [file pcbi.1007054.s006.TIF]

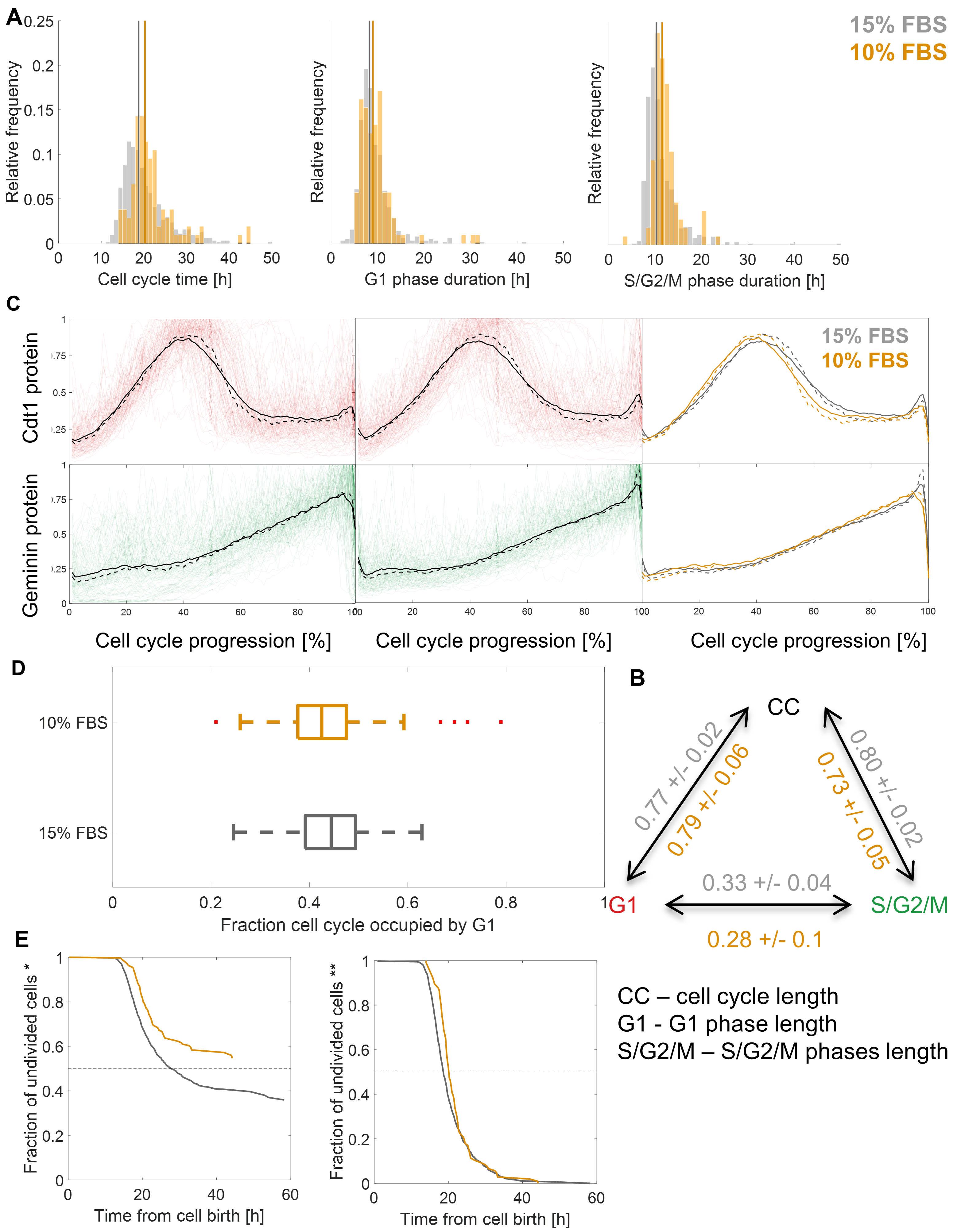

Supplement: S7 Fig — (A) Comparison of cell cycle, G1 phase and combined S/G2/M phases durations for two serum (FBS) concentrations. Calculations were performed based on 105 and 642 measurements for 10% and 15% of FBS, respectively. Histograms were normalized, the height of each bar is equal to the probability of selecting an observation within the corresponding bin interval, and the height of all of the bars sums up to 1. All distributions have characteristic lognormal-like shape; additional information about medians are included directly on the plots. Lower dose of serum causes extension of G1 and S/G2/M phases and as a consequence of the whole cell-cycle length. Changes in the lengths of the cell cycle and of the S/G2/M phases are statistically significant (Wilcoxon rank sum test). (B) Pearson rank correlations between lengths of the phases and the cell cycle. Standard deviations were calculated using Monte Carlo cross validation and 10,000 iterations. (C) Comparison between protein dynamics. Each line denotes one cell, black solid line is mean trace, black dashed line is median trace. The division moments were selected using procedure described in Methods section. (D) G1 phase takes proportionally less time under 10% FBS (Wilcoxon rank sum test), as it is shown in the boxplot (105 samples for 10% FBS, and the same number of randomly selected samples for 15% FBS). (E) Survival function mapping division events onto time, based on cells that divided at least twice during the experiment, so the time of birth and death could be estimated. Kaplan-Meier curve maps division events onto time, including cells which divided only once, so that either their birth or death are not known. Significant difference between 10% FBS and 15% FBS is observed only when incomplete cell cycles are included. (TIF) [file pcbi.1007054.s007.tif]

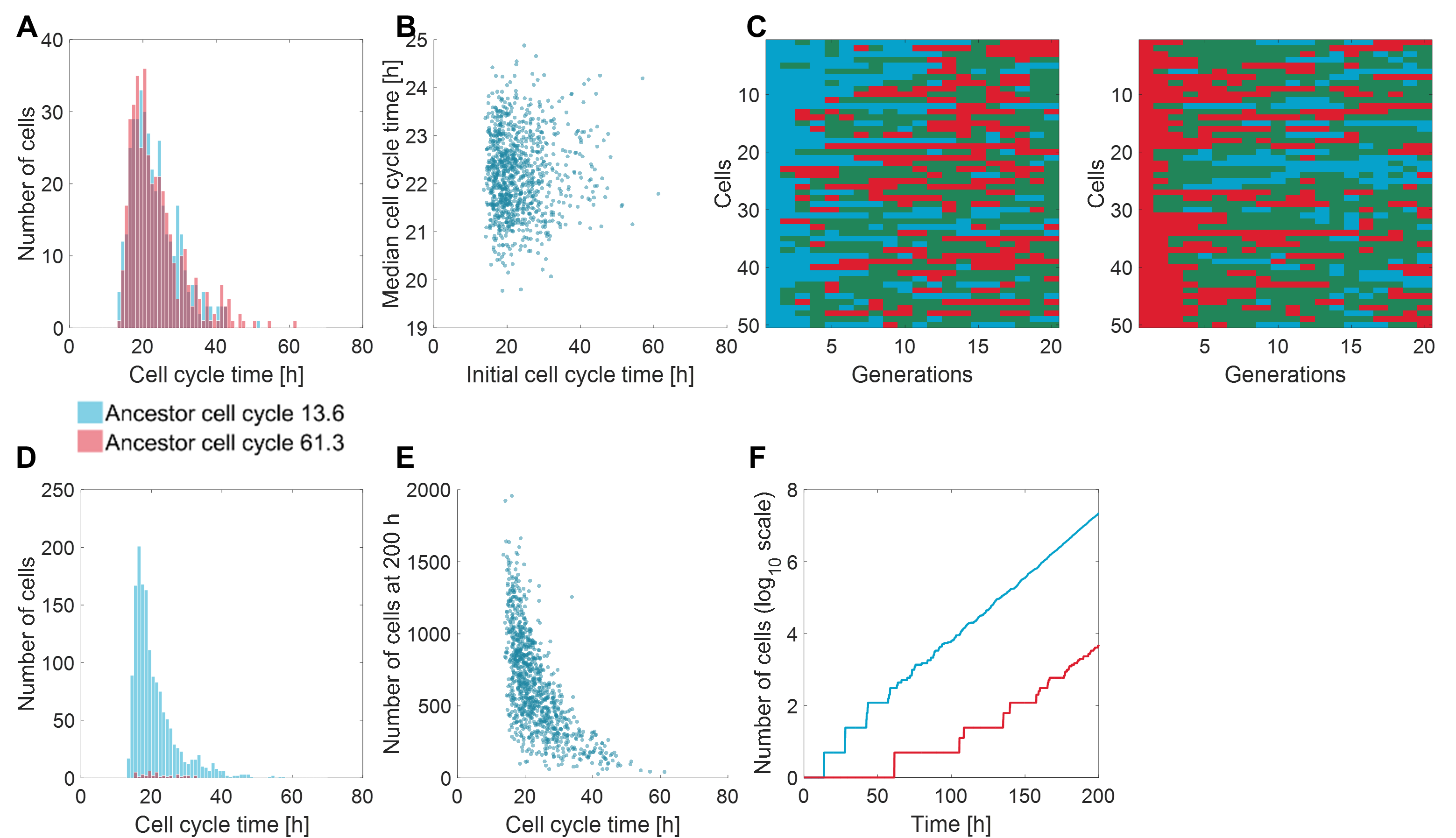

Supplement: S8 Fig — (A) Histograms of cell-cycle lengths for a single ancestor cell and its progeny. After each of 4,000 divisions along a single line of descent, one randomly chosen progeny was used for analysis. Blue and red color represent cases with low (13.6 h) and high (61.3 h) initial cell-cycle length, respectively. The medians in both cases are similar (21.9 h and 21.8 h). (B) The scatter plot of initial cell-cycle length and median cell-cycle length after 400 generations. No correlation is observed is significant statistically (ρ = -0.04). (C) Heat maps representing changes in cell-cycle durations in next generations. Three colors represent different cell-cycle lengths: blue for measurements below the first quartile; red for measurements above third quartile, and green for measurements within the interquartile range. (D) Histograms of cell-cycle lengths for a population started from a single ancestor at 200 h of observation. Blue and red colors represent cases with low (13.6 h) and high (61.3 h) initial cell cycle length, respectively. (E) Scatter plot of initial cell-cycle length and population size after 200 h. Strong negative correlation is observed (ρ = -0.65). Growth curves for two extreme cases. Blue and red colors represent cases with low (13.6 h) and high (61.3 h) initial cell-cycle length (respectively). (F) Descendants of ancestor cells are identified and counted. Growth curves show differences between two cell populations. (TIF) [file pcbi.1007054.s008.tif]

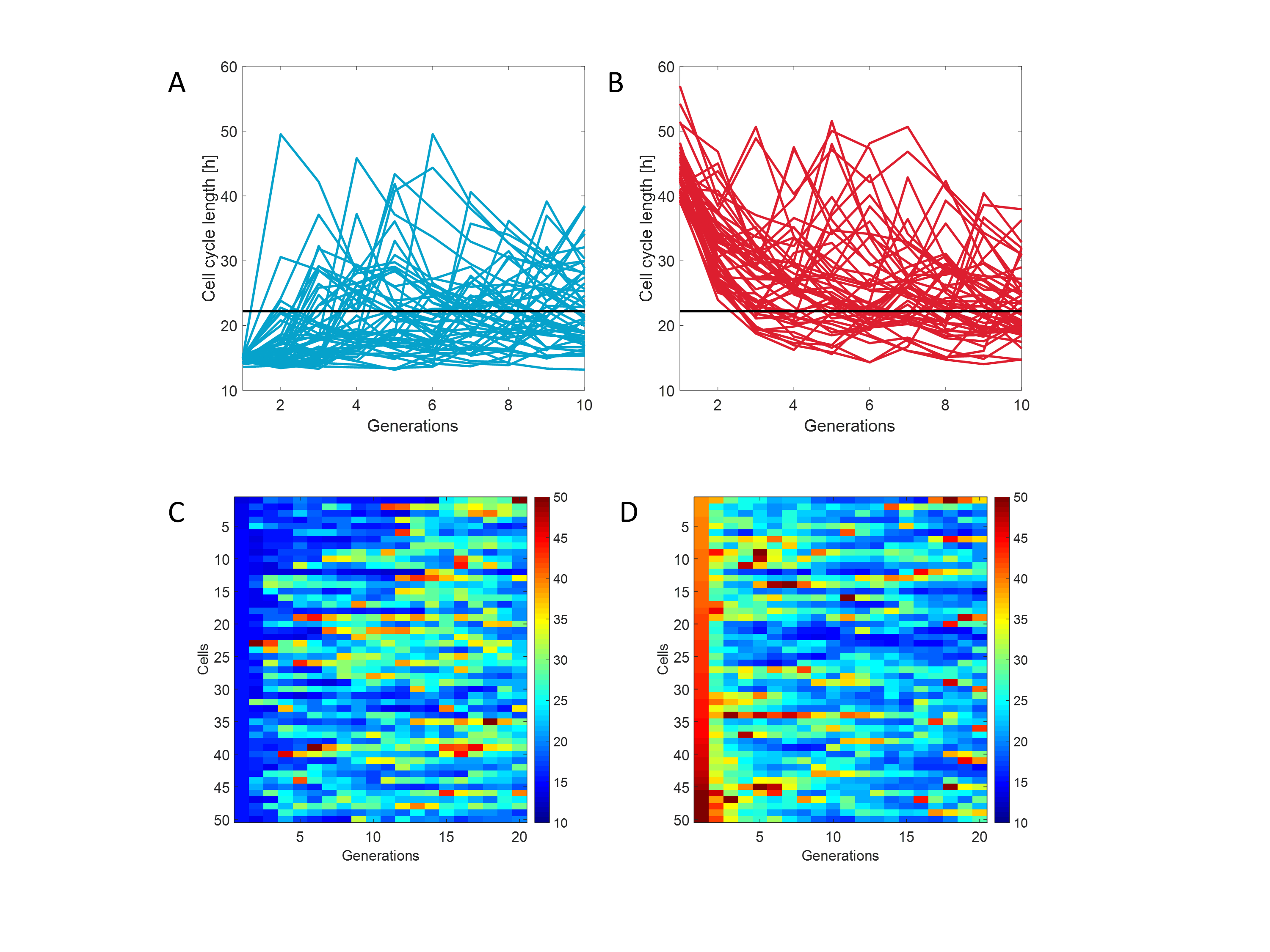

Supplement: S9 Fig — (A, B) Ten extreme cases presented in the form of chart, where x axis represents generation number, y axis cell-cycle length. (C, D) Fifty extreme cases presented in the form of a heat map, where x axis represents generation number, y axis represent single-cell lineage and color denotes cell-cycle length. (TIF) [file pcbi.1007054.s009.TIF]

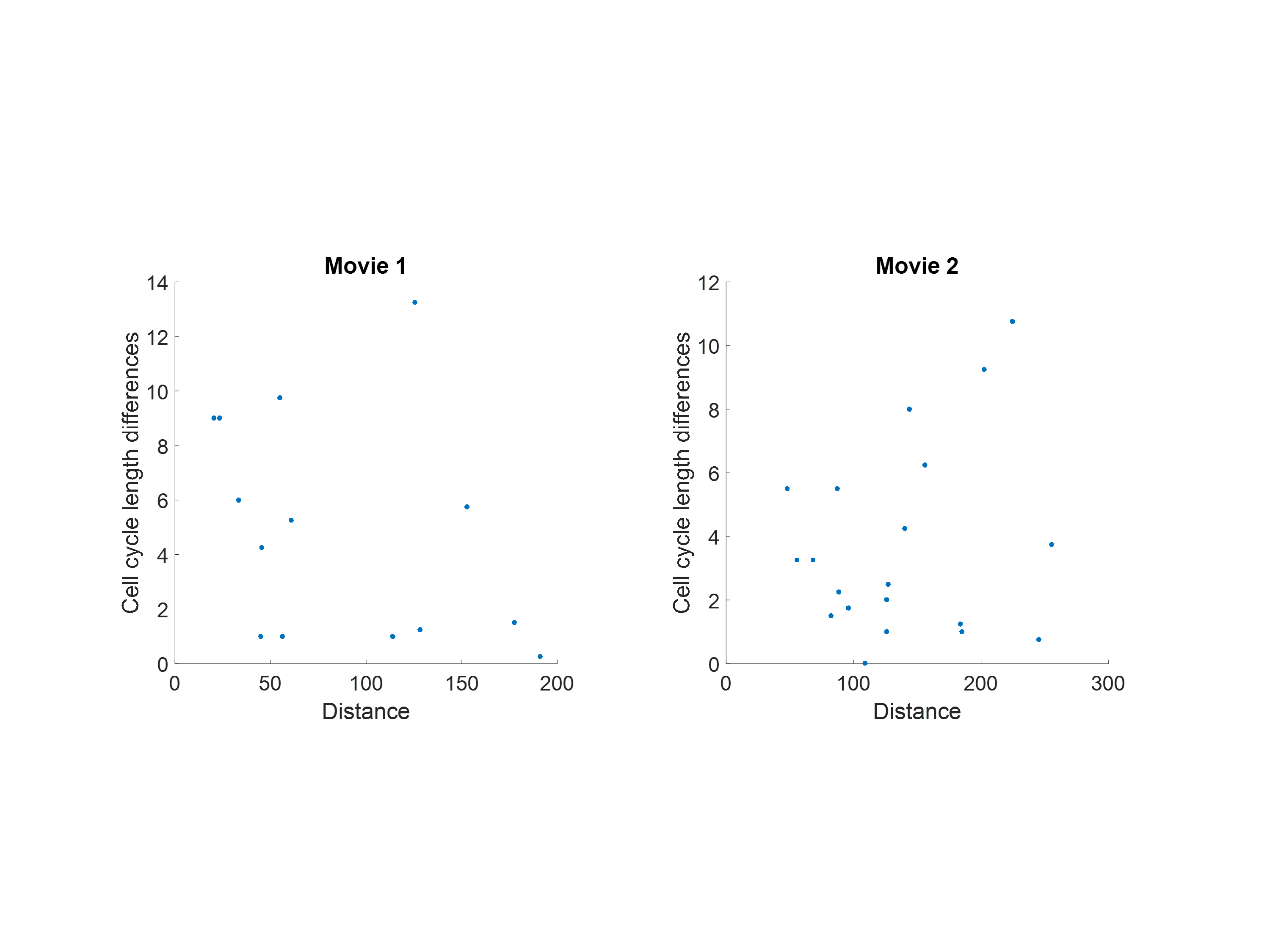

Supplement: S10 Fig — (TIF) [file pcbi.1007054.s010.TIF]

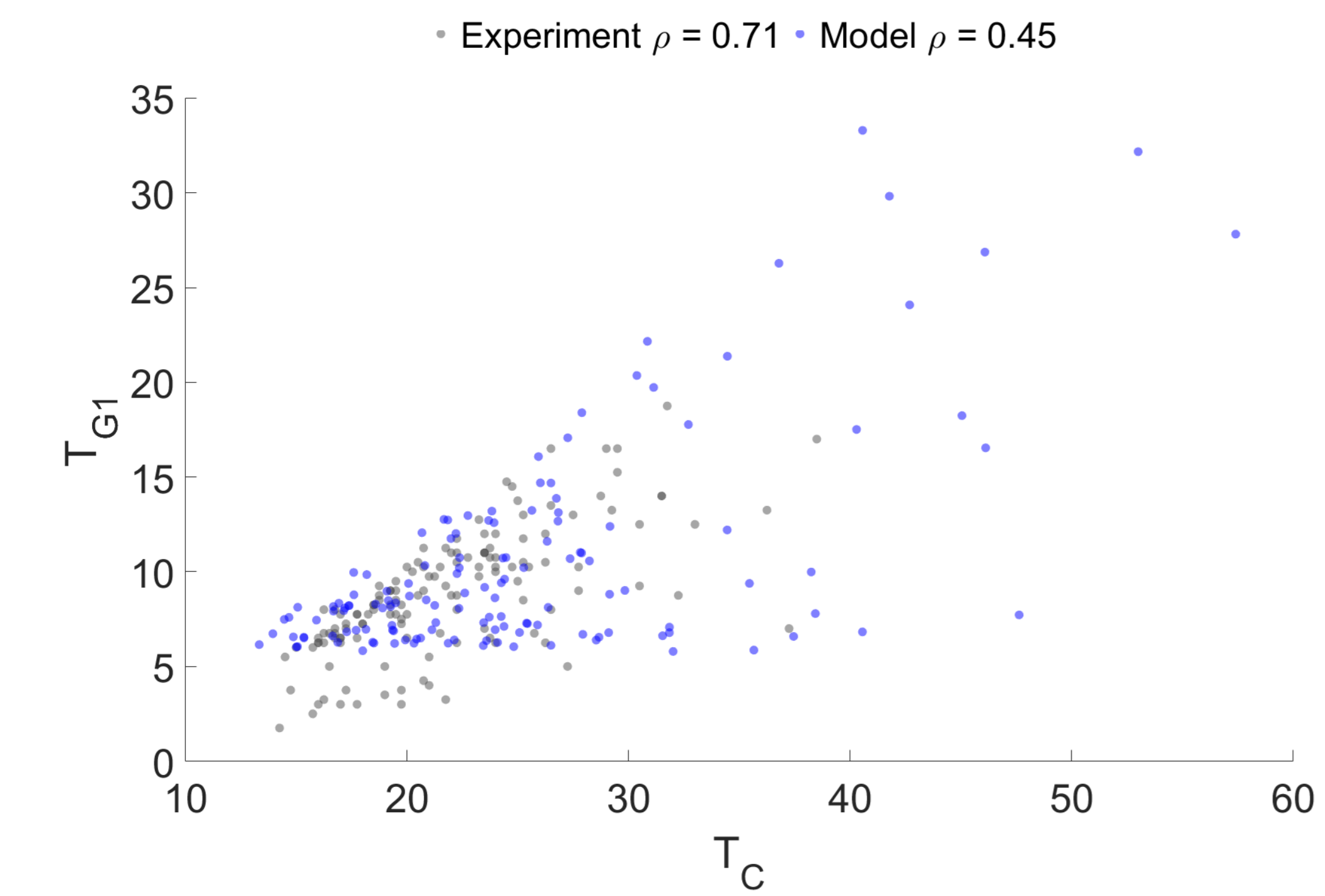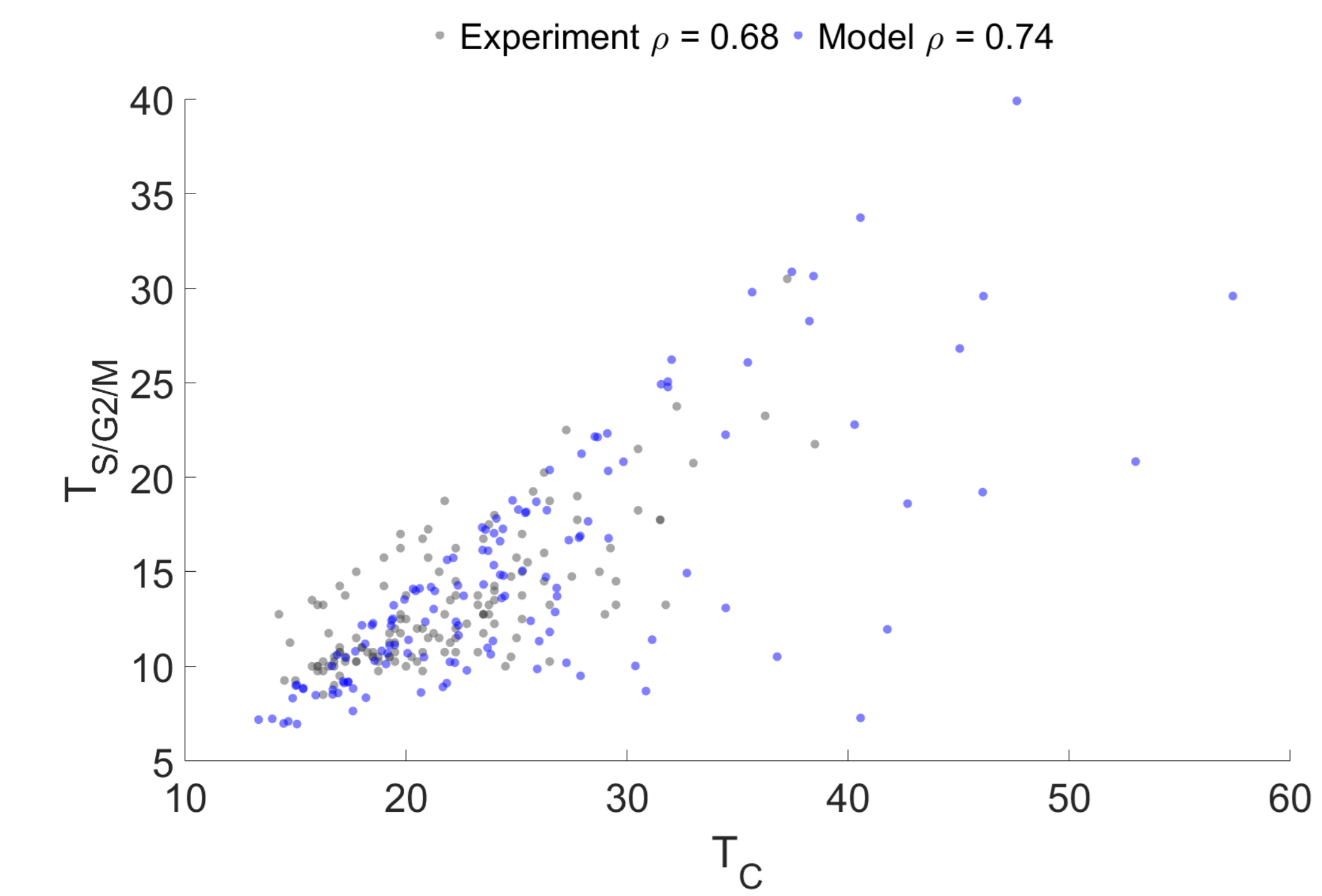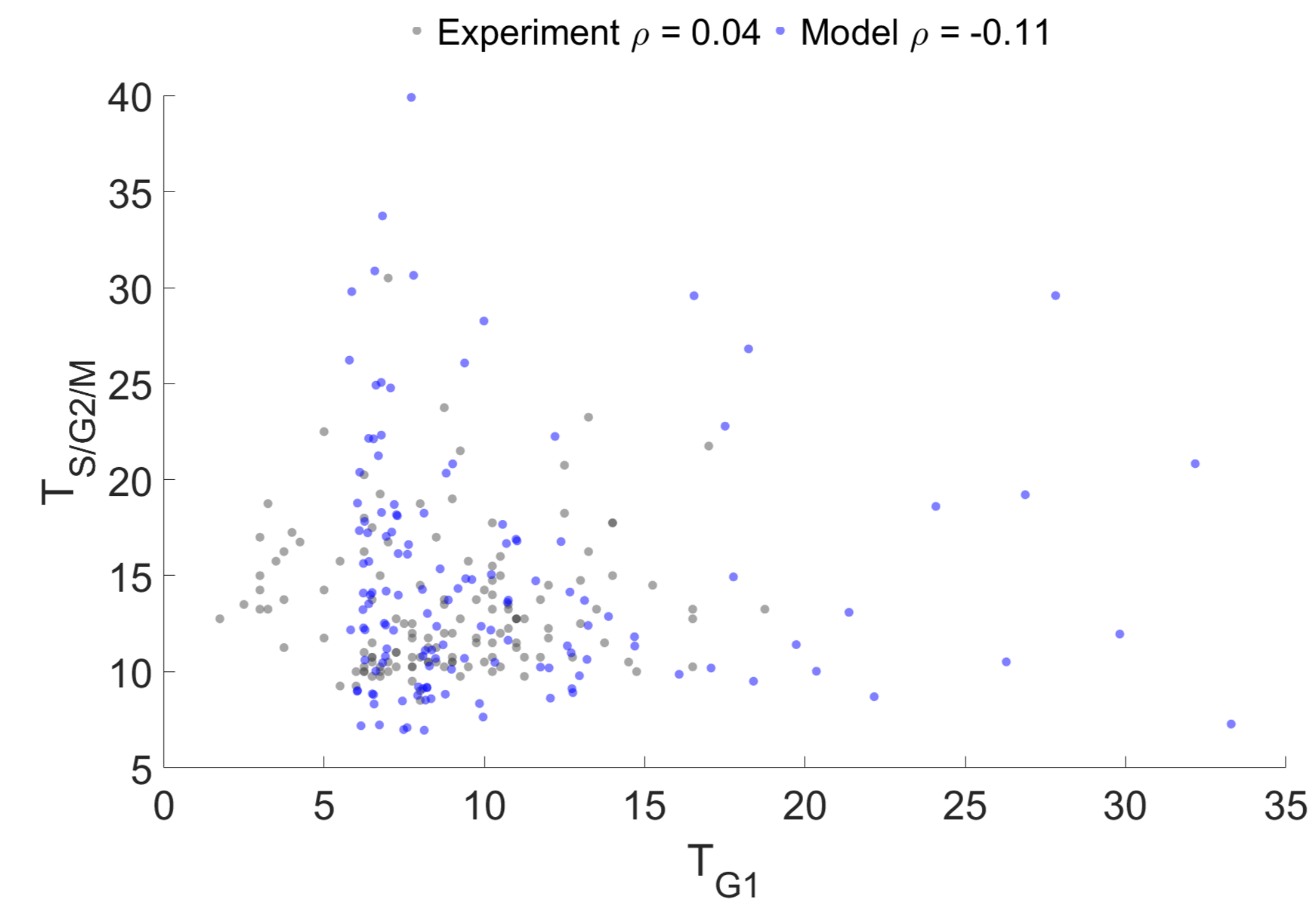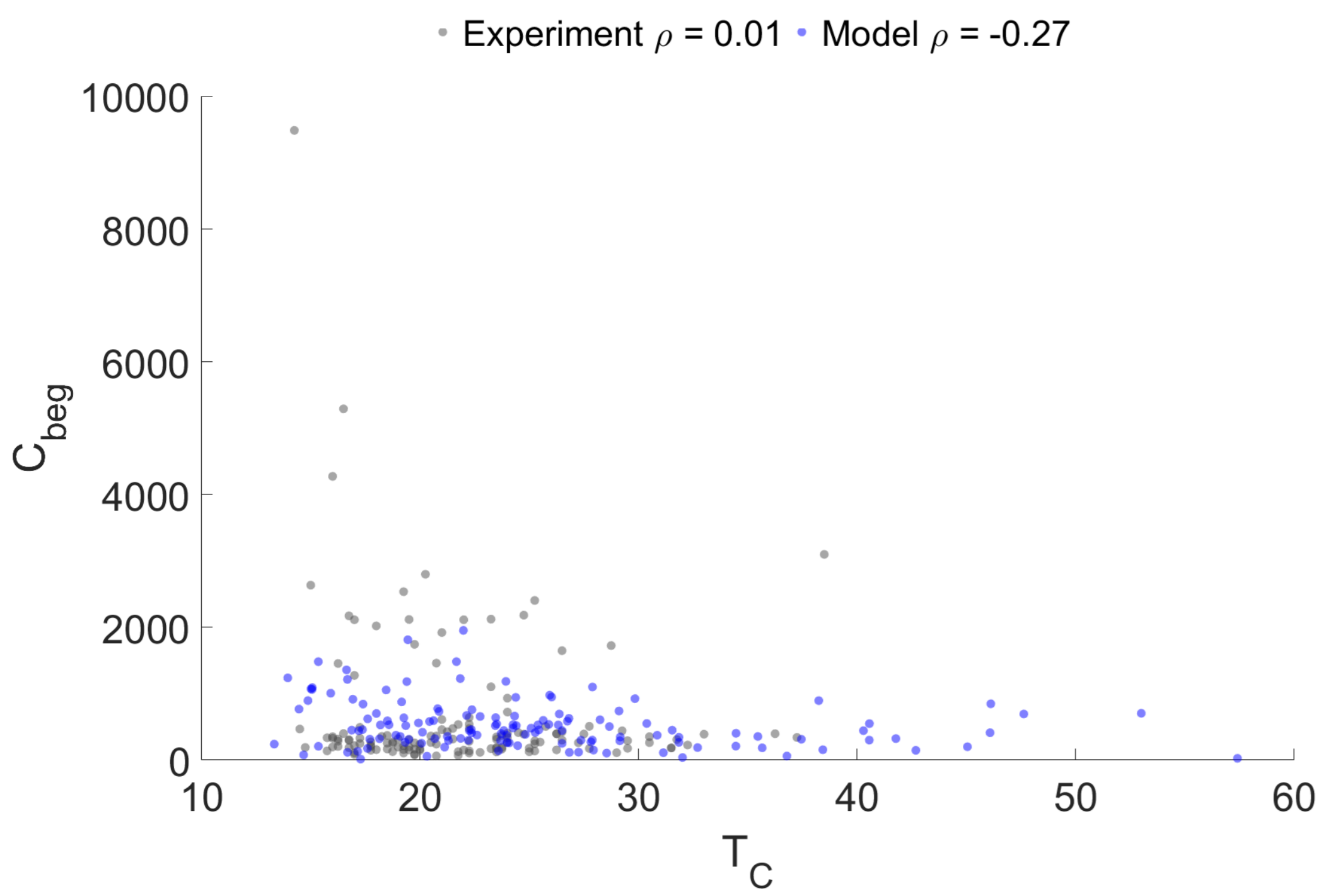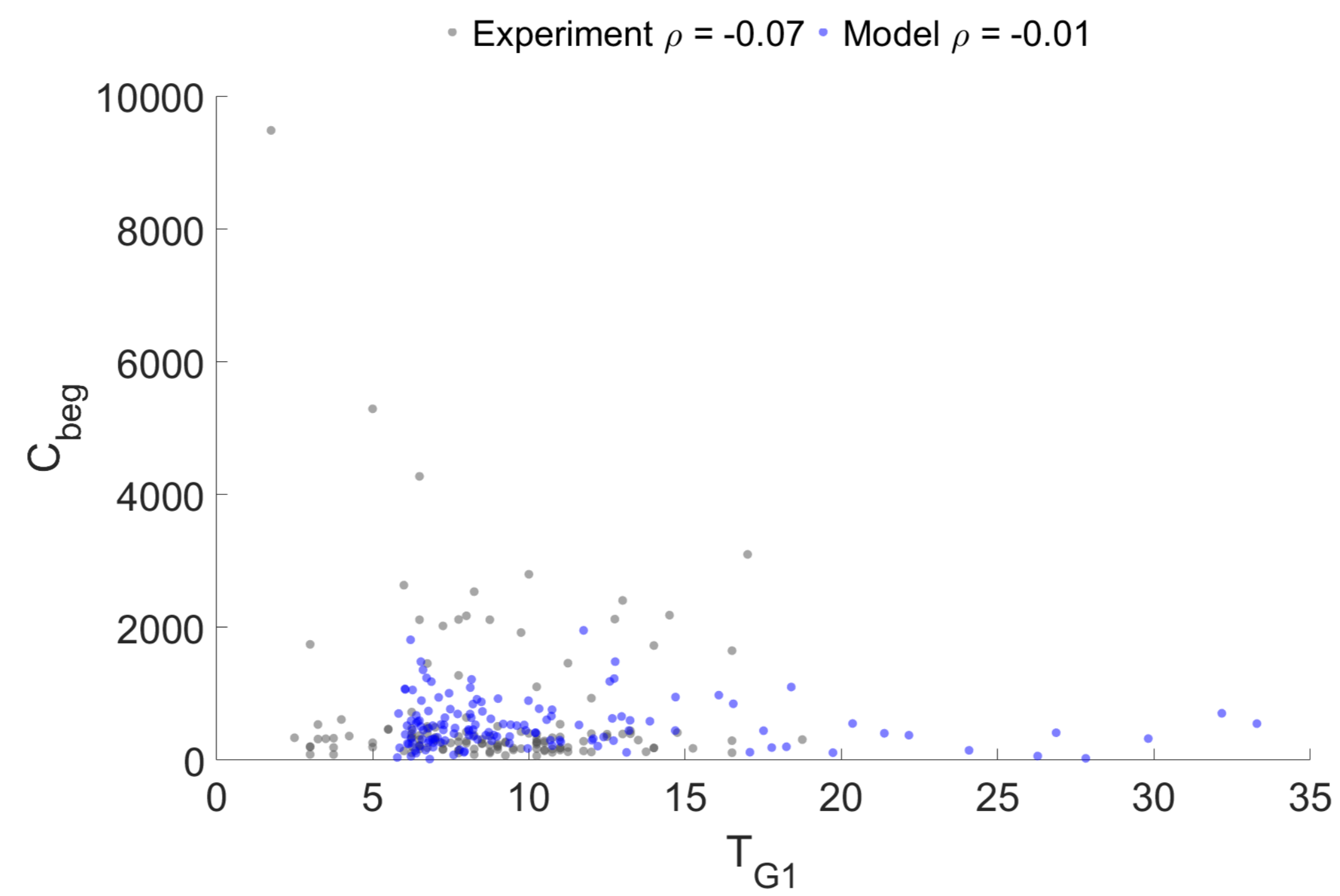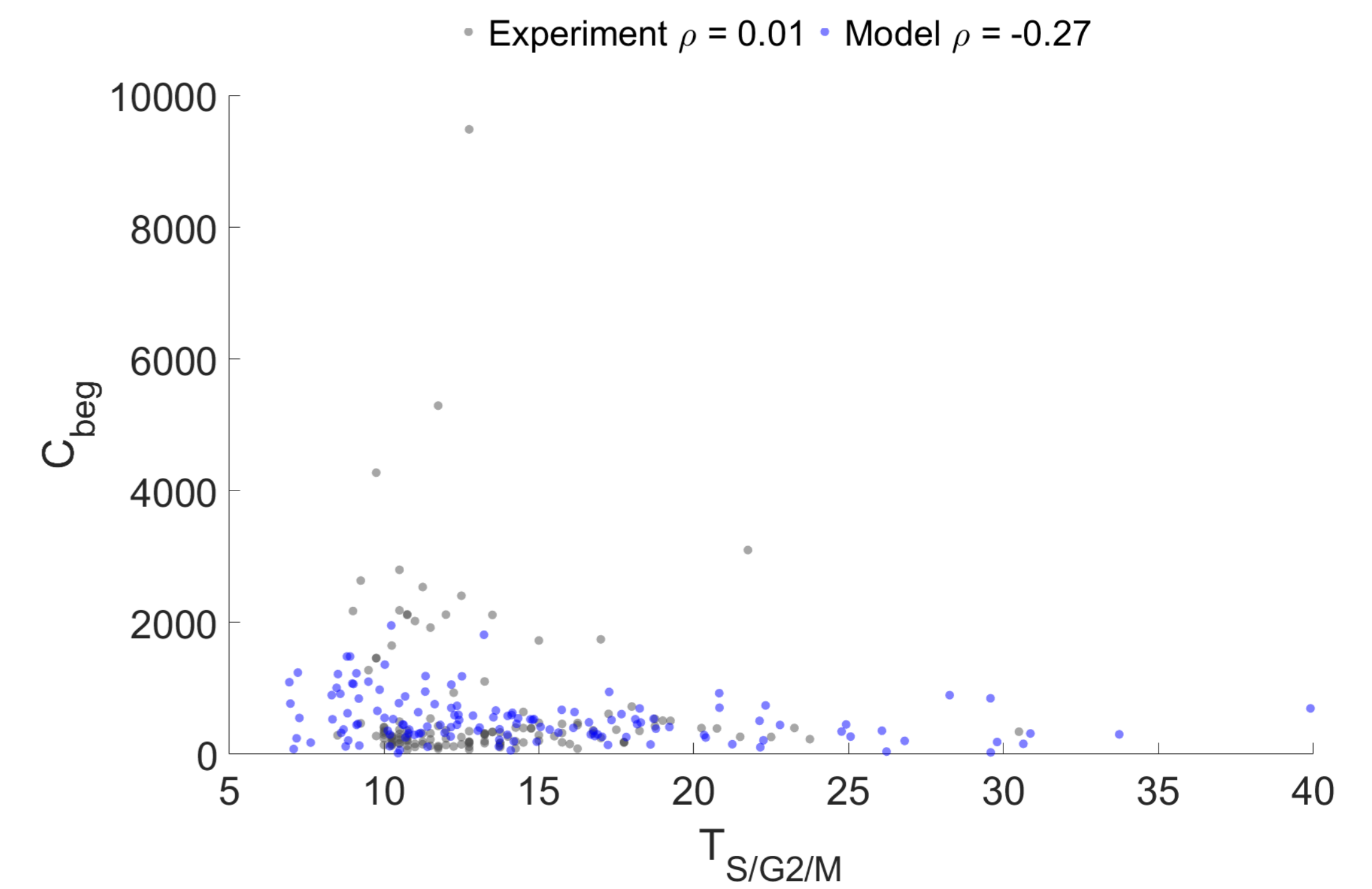

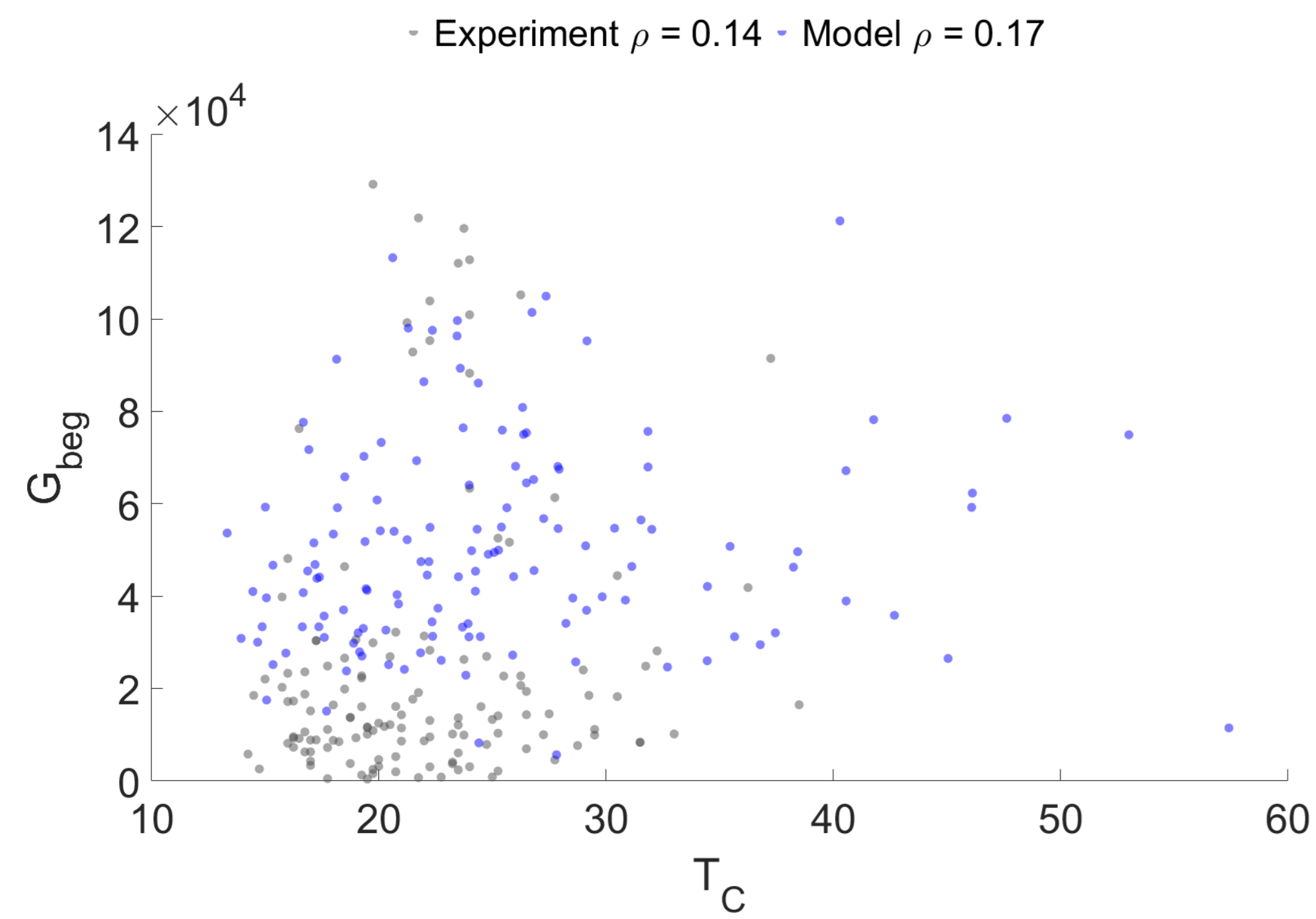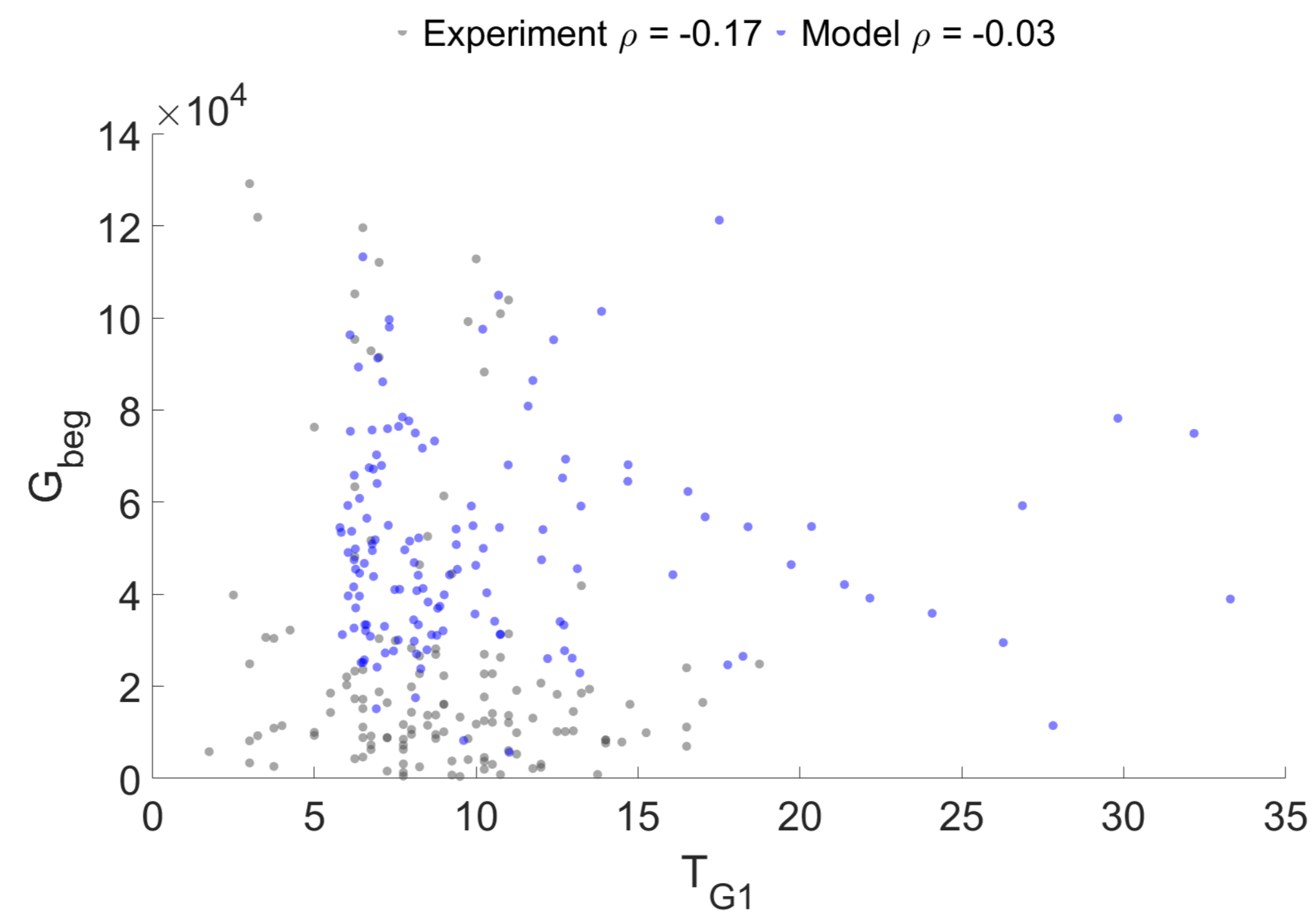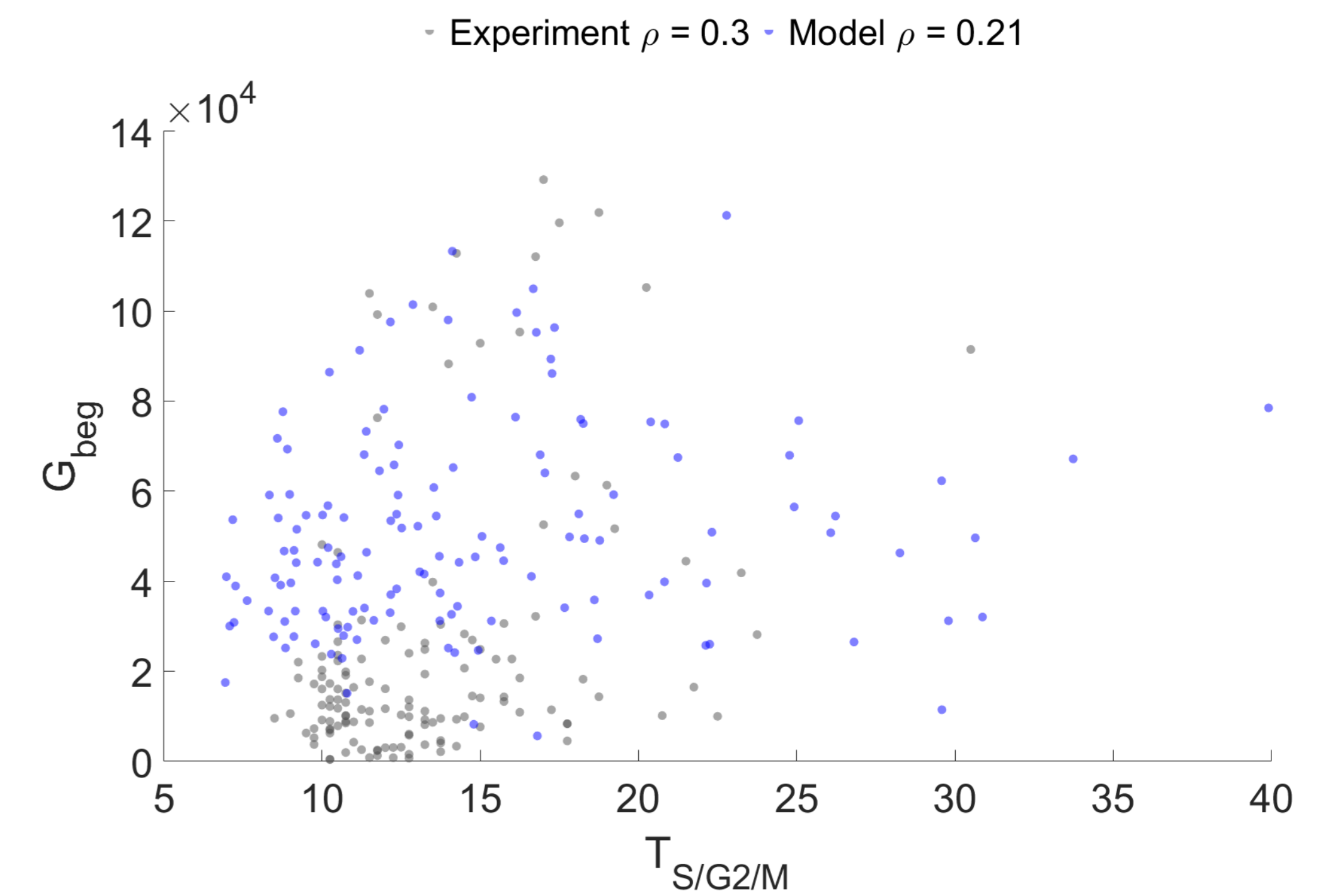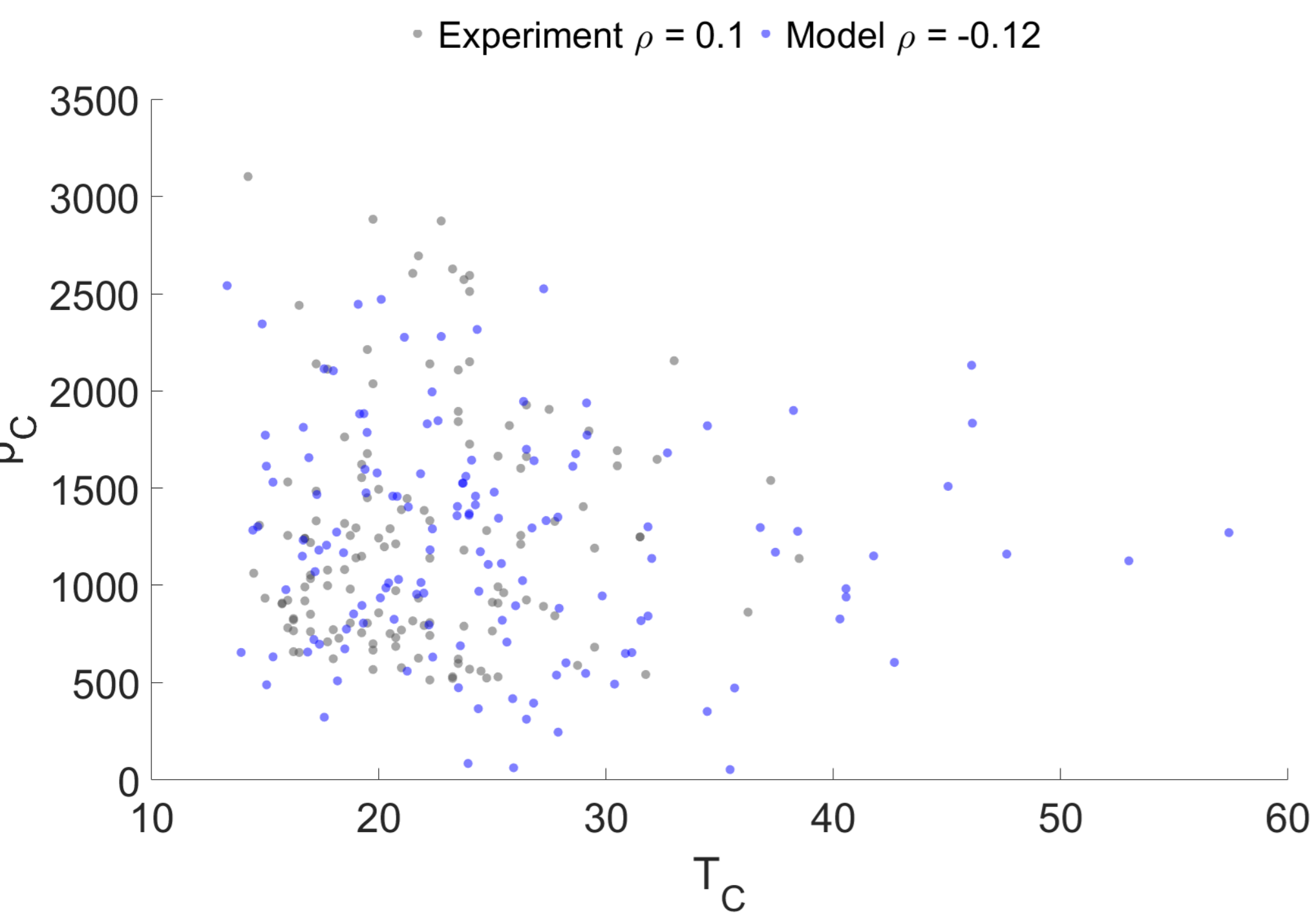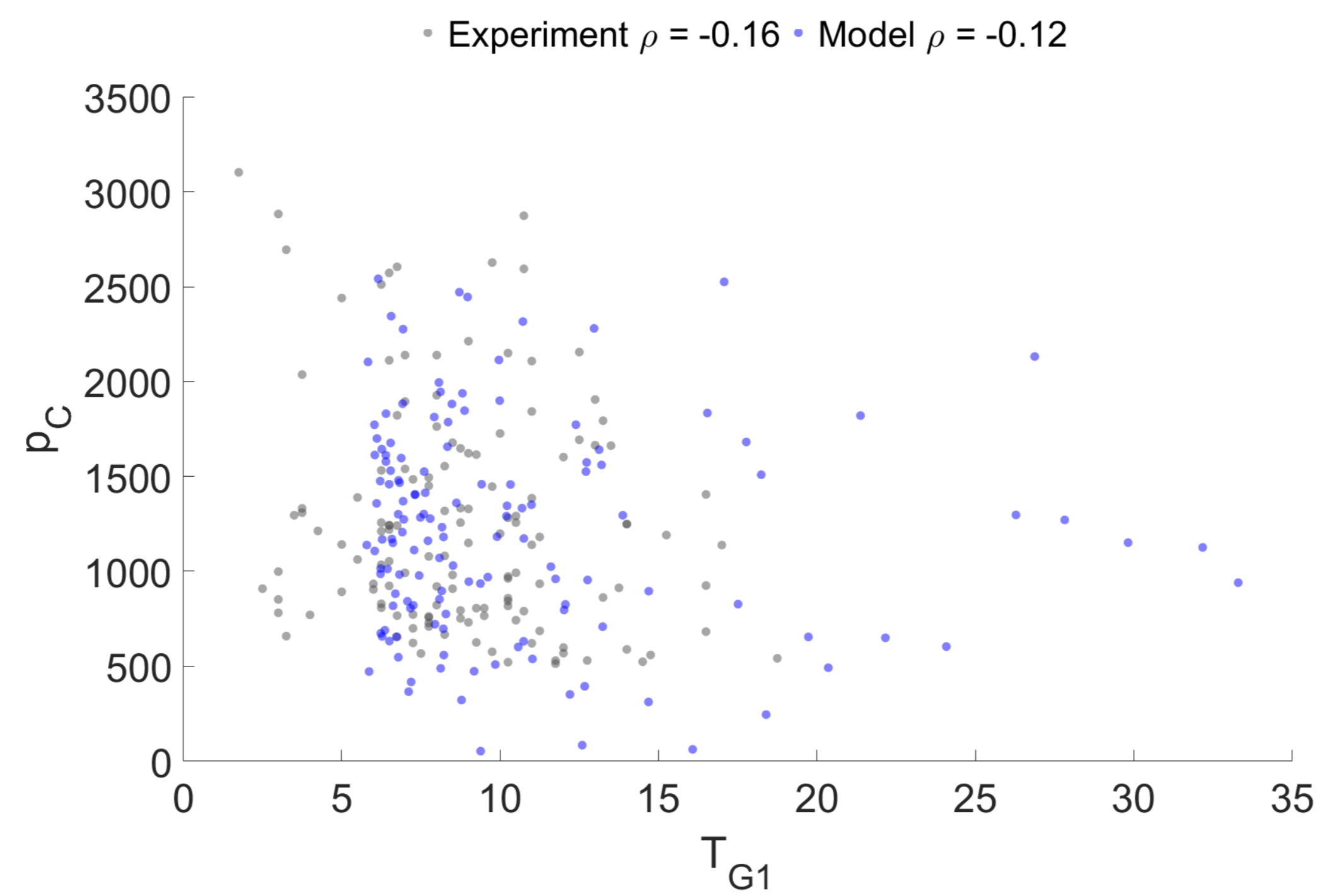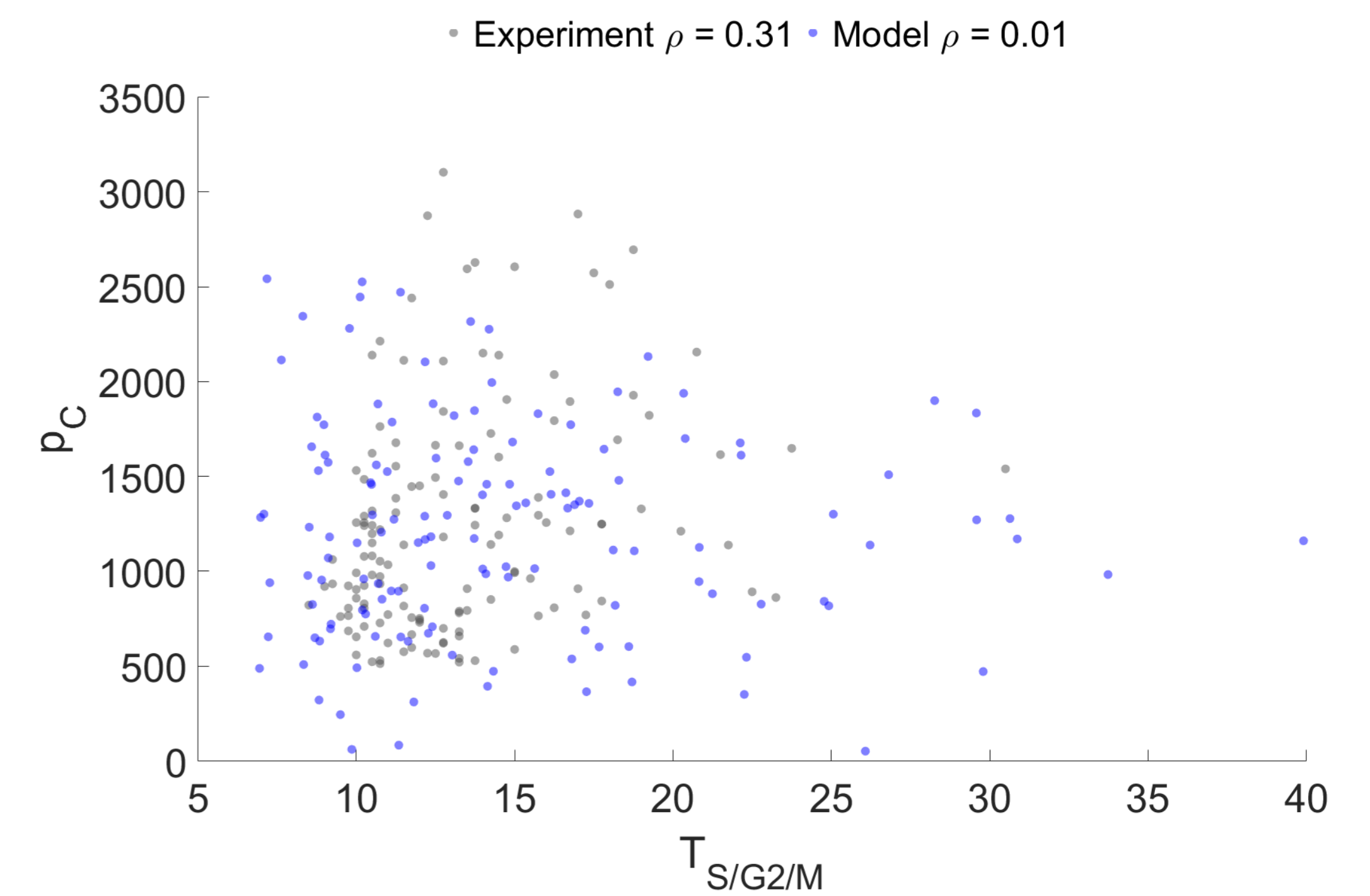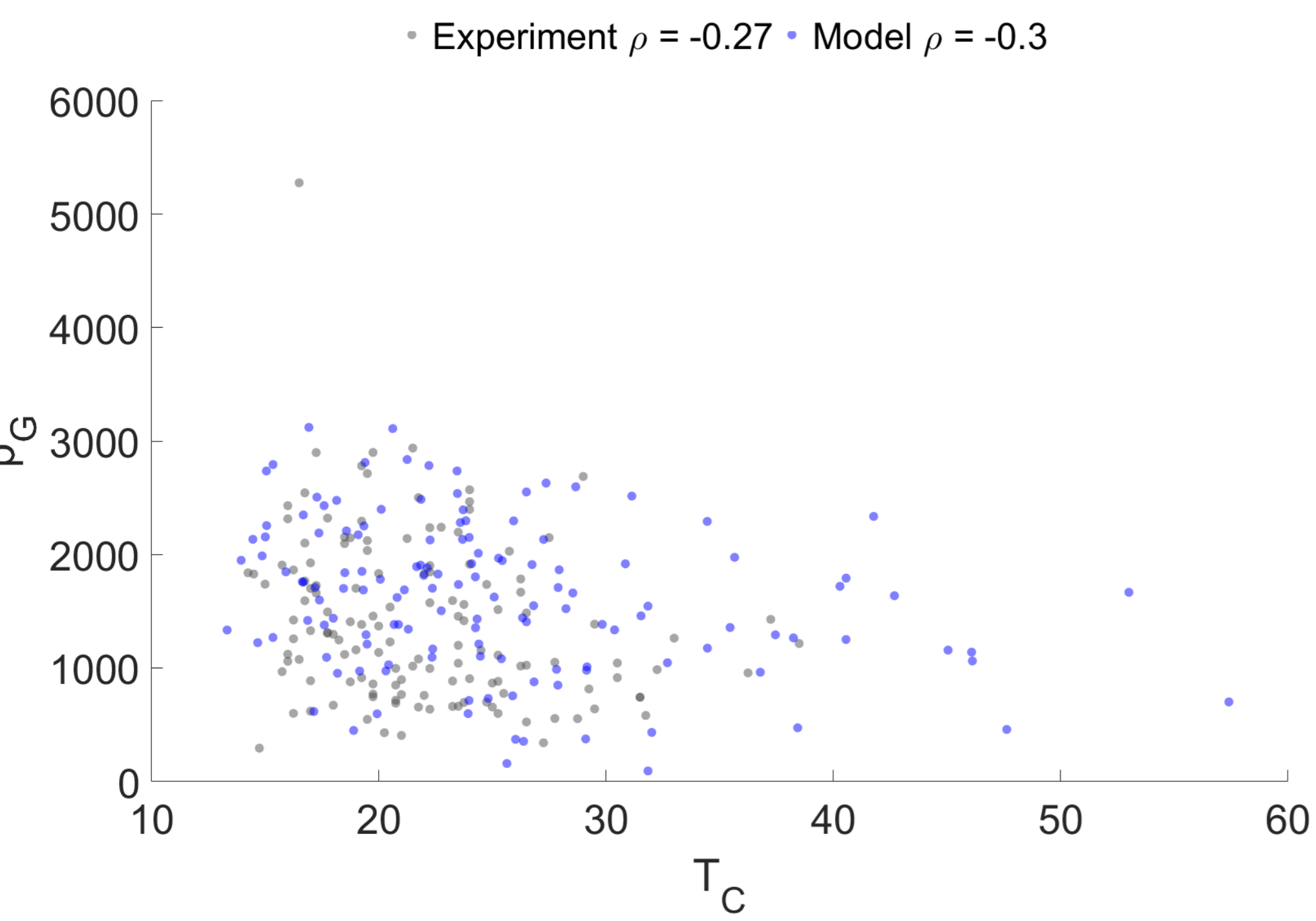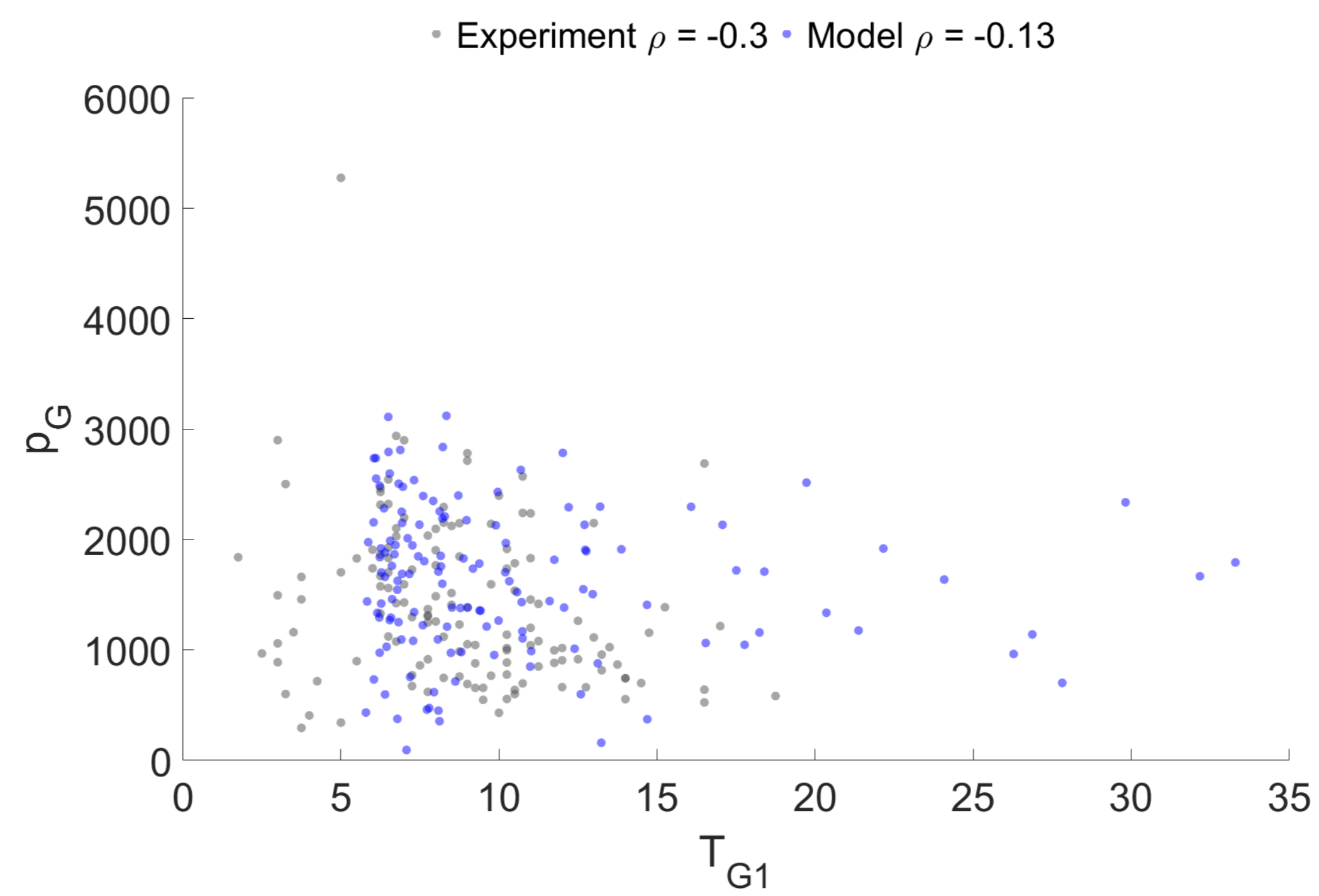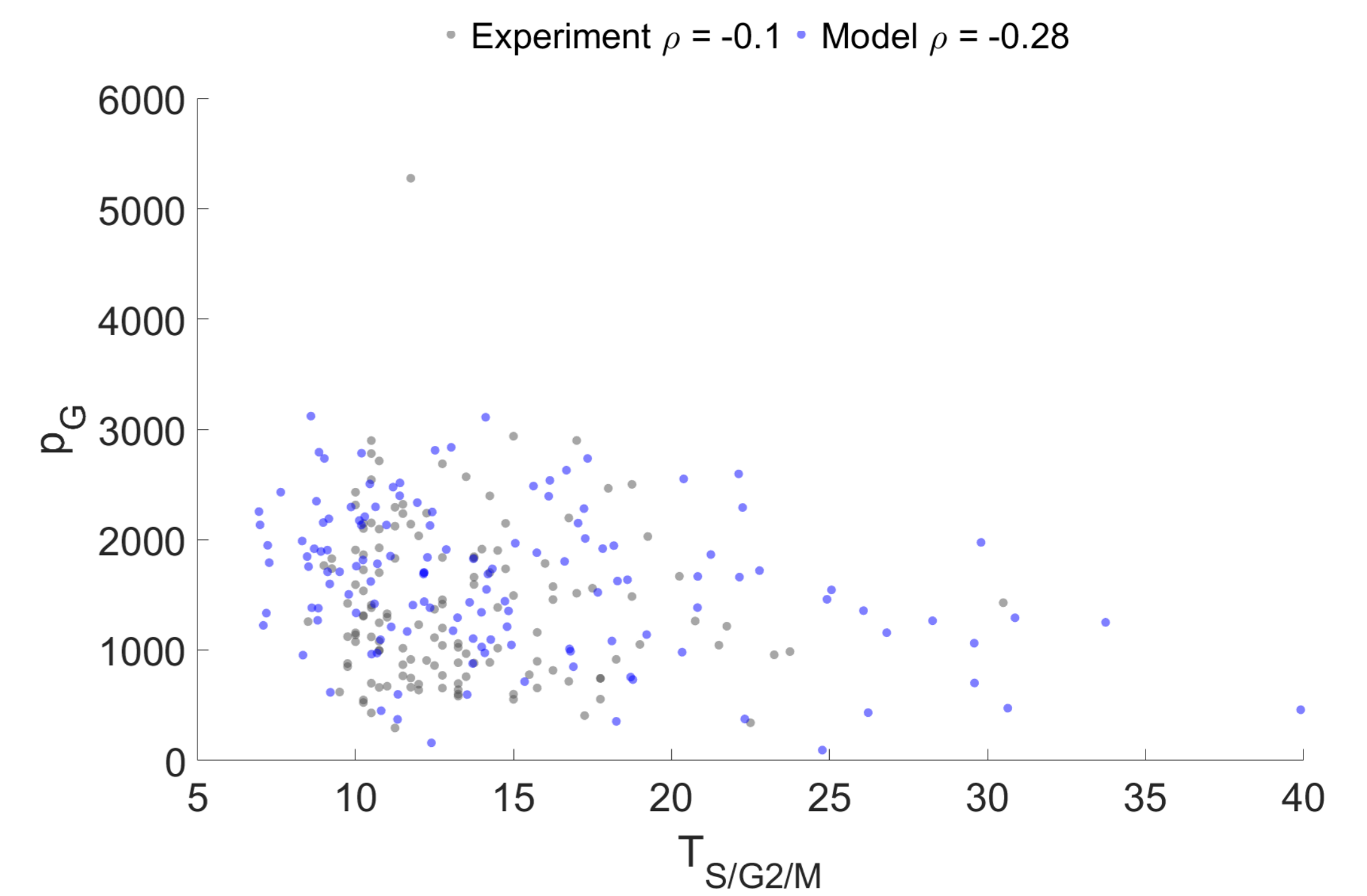

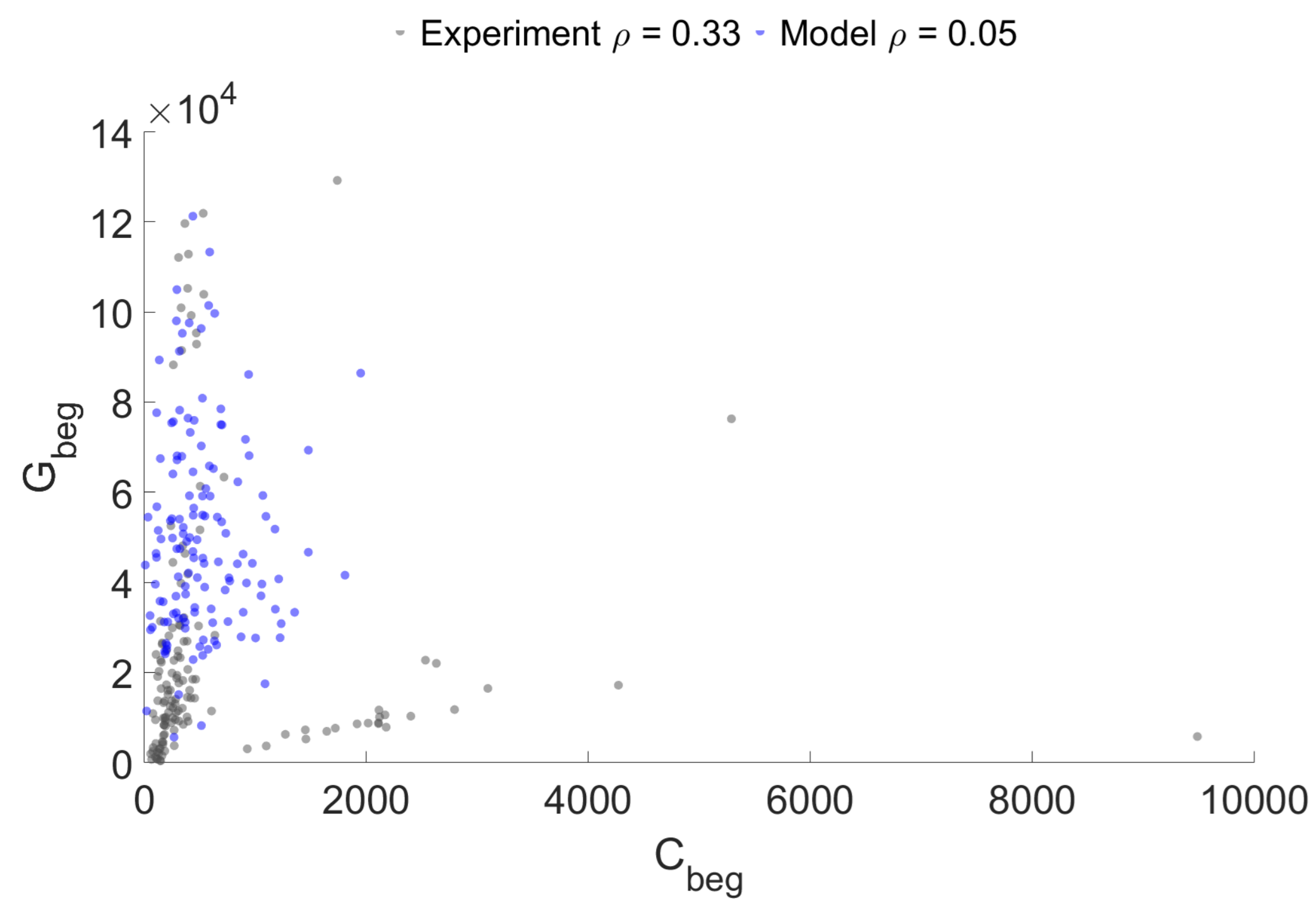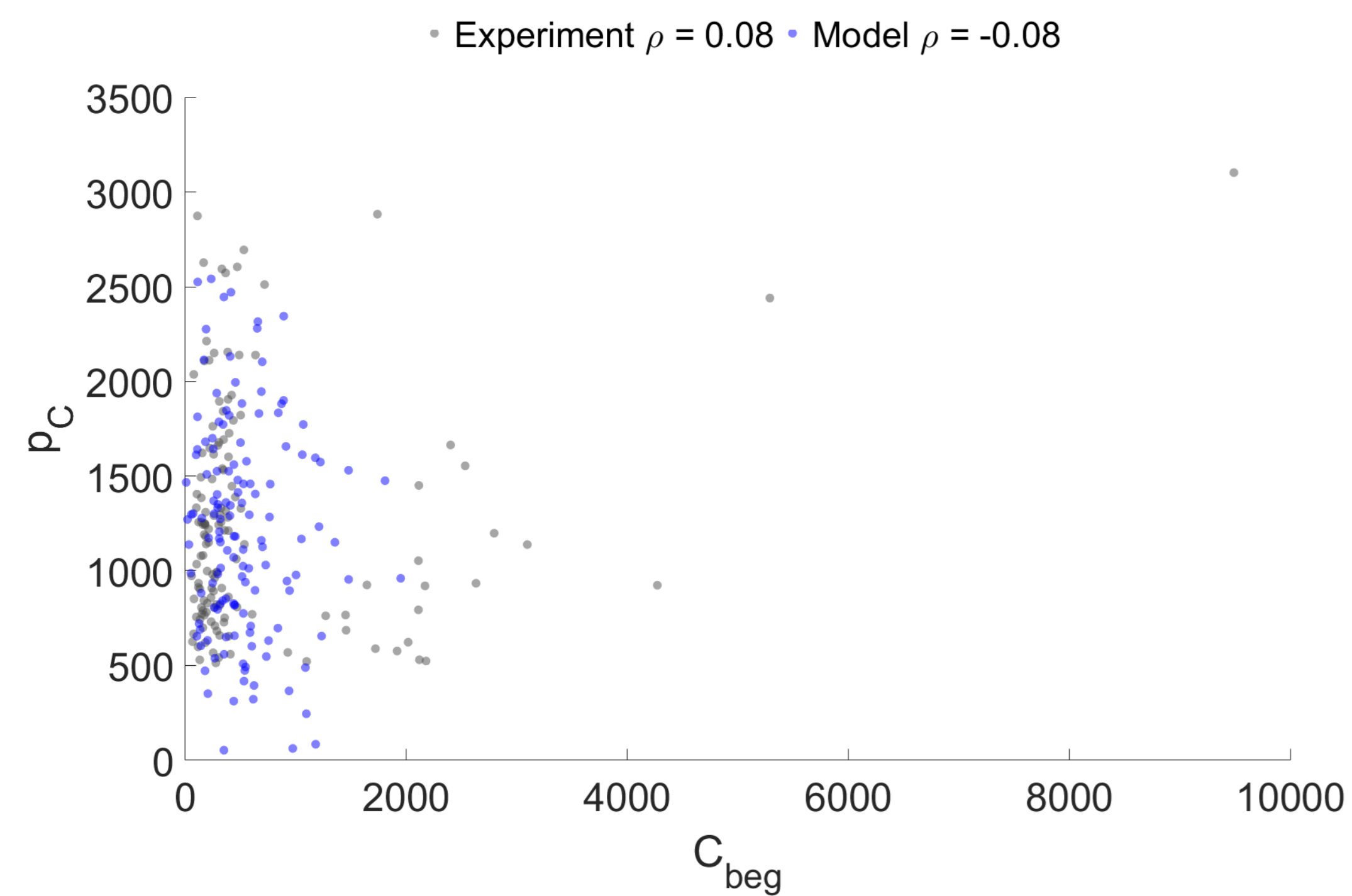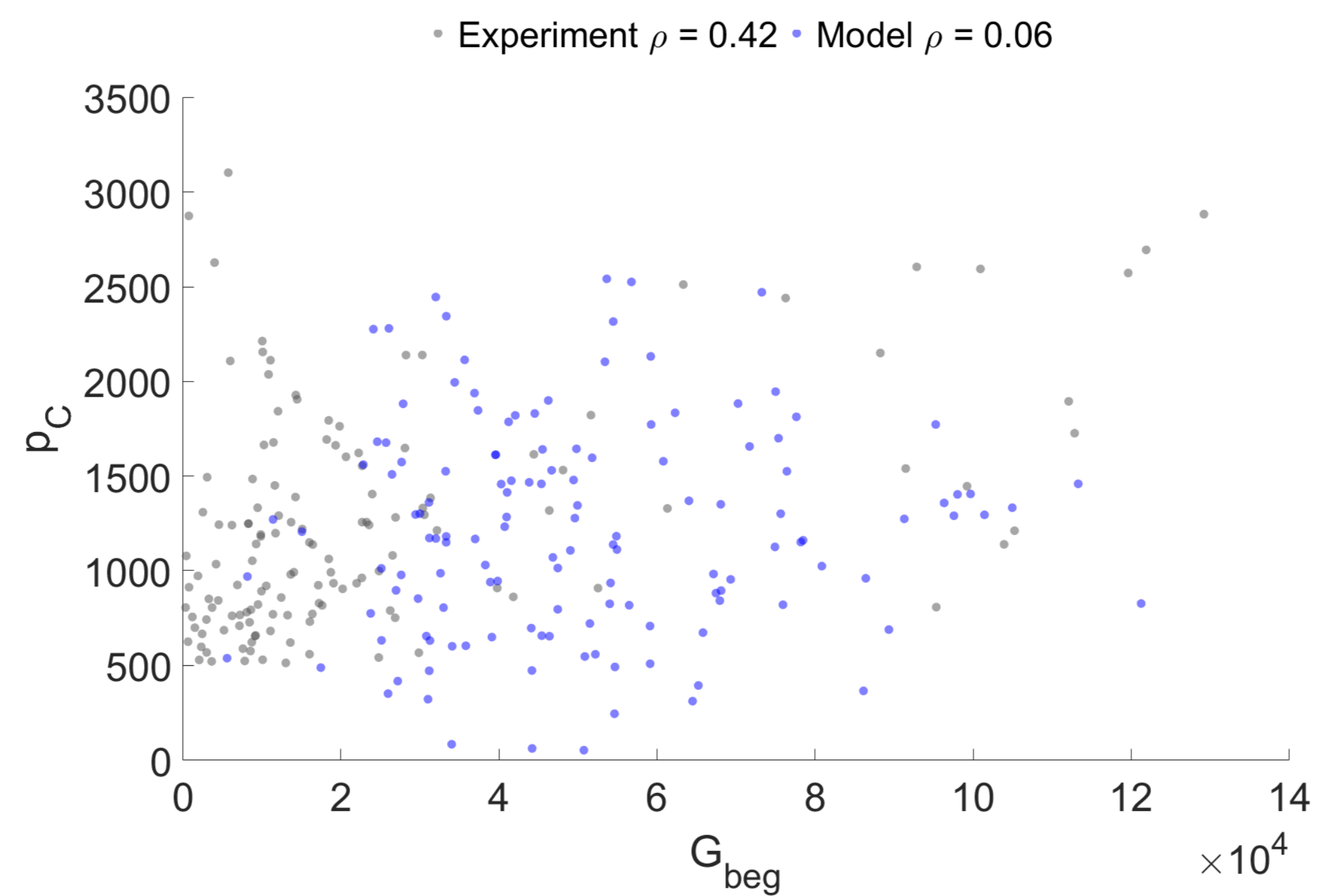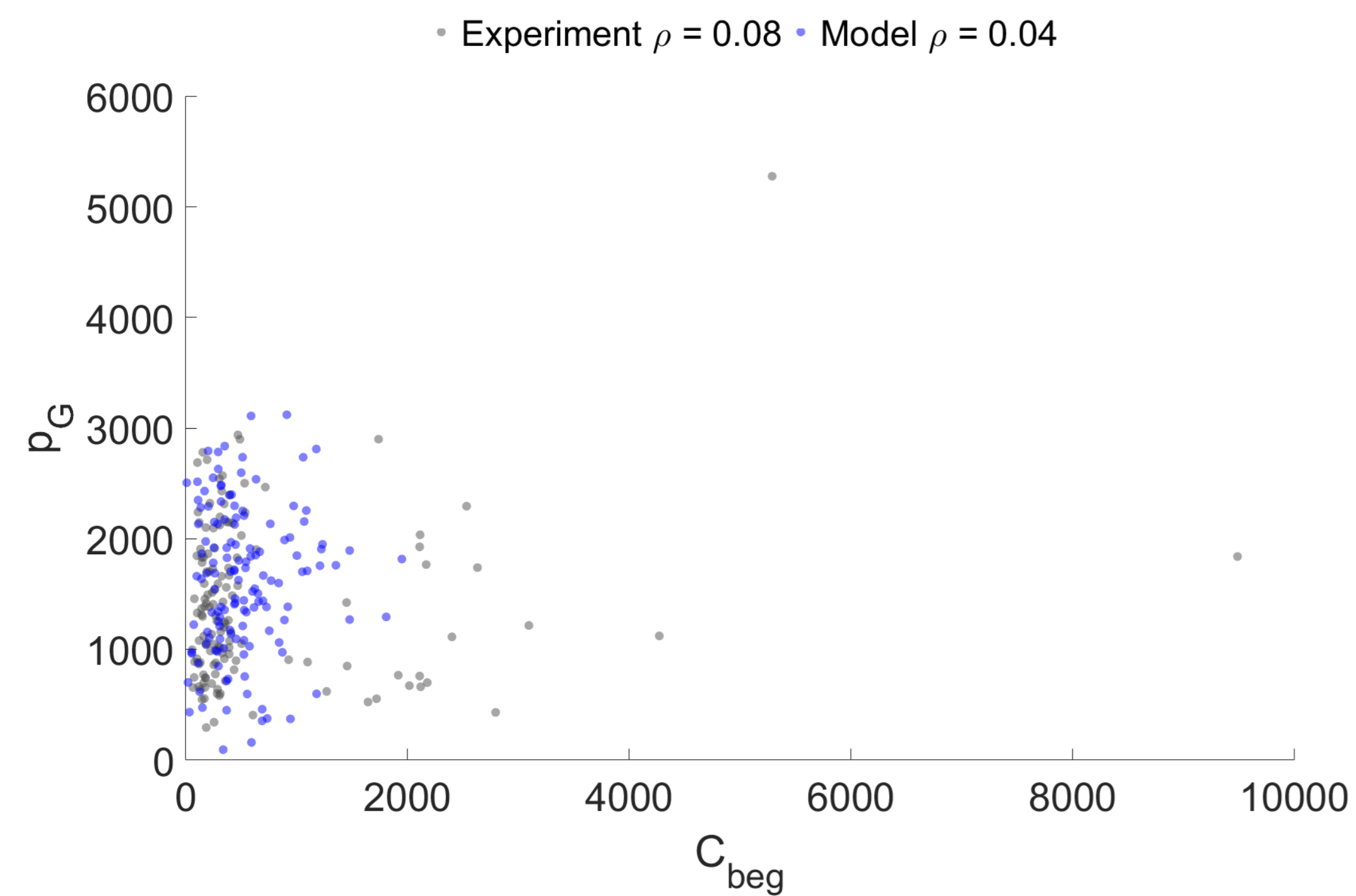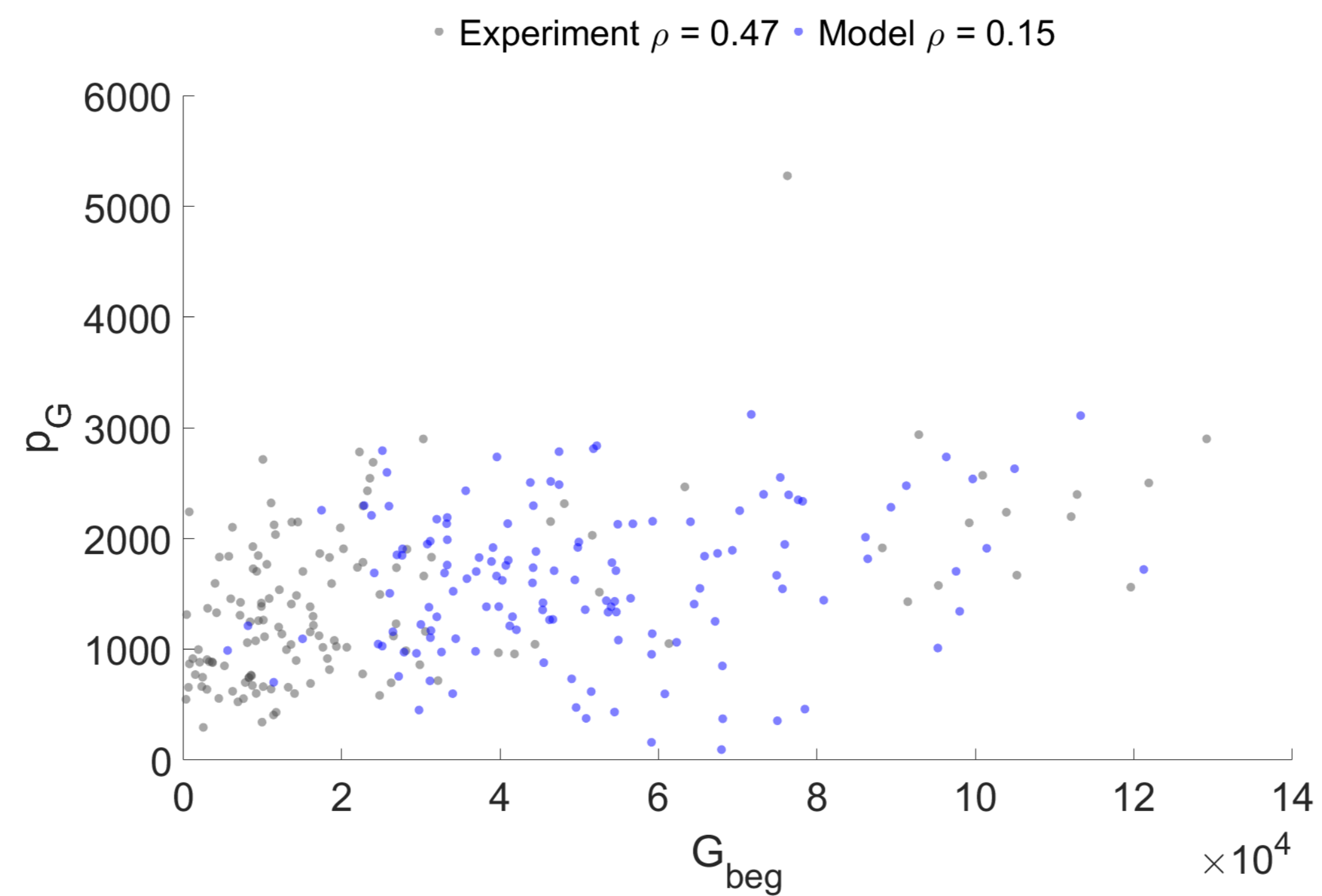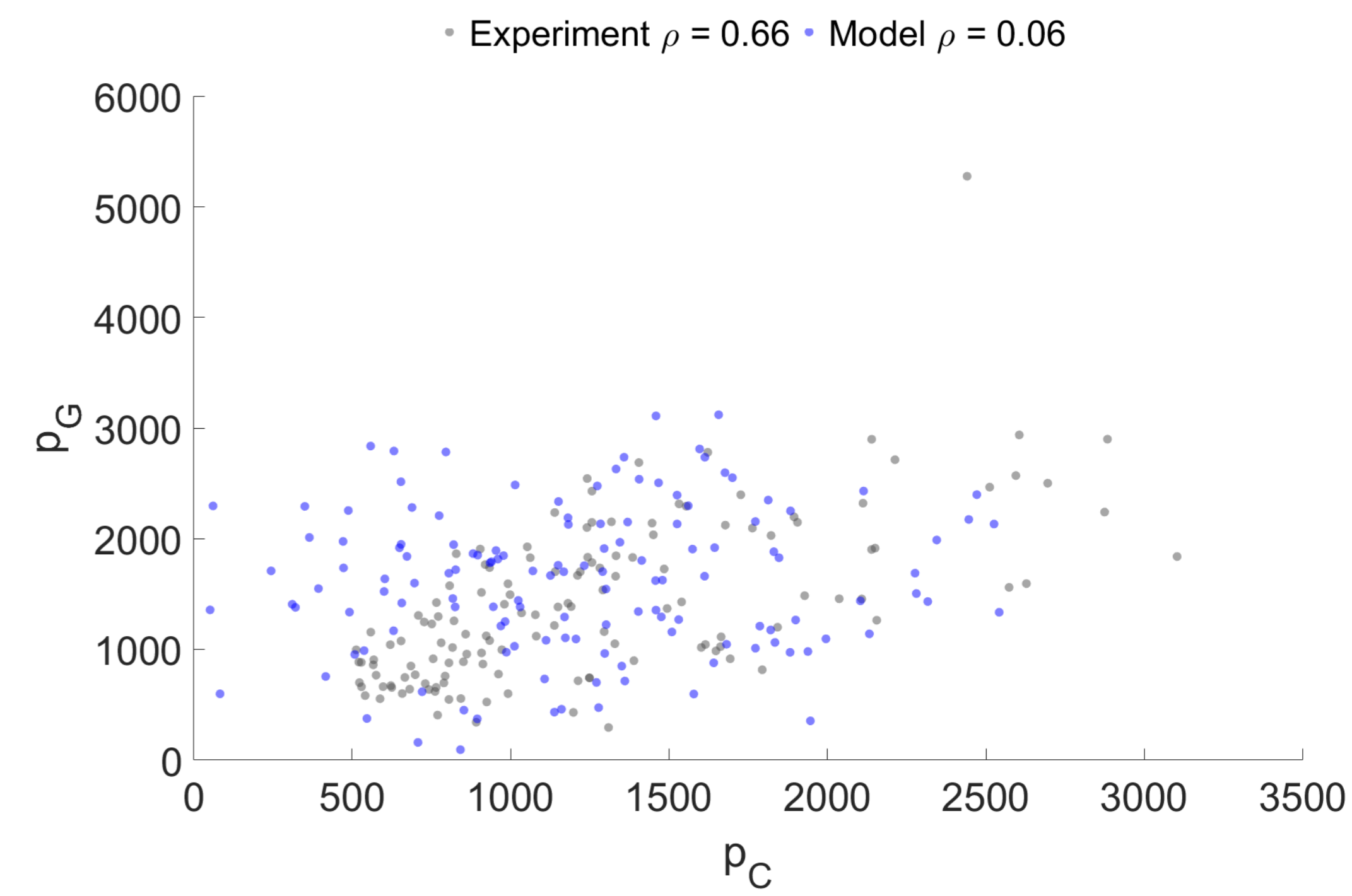

Supplement: S11 Fig — (PDF) [file pcbi.1007054.s011.pdf]

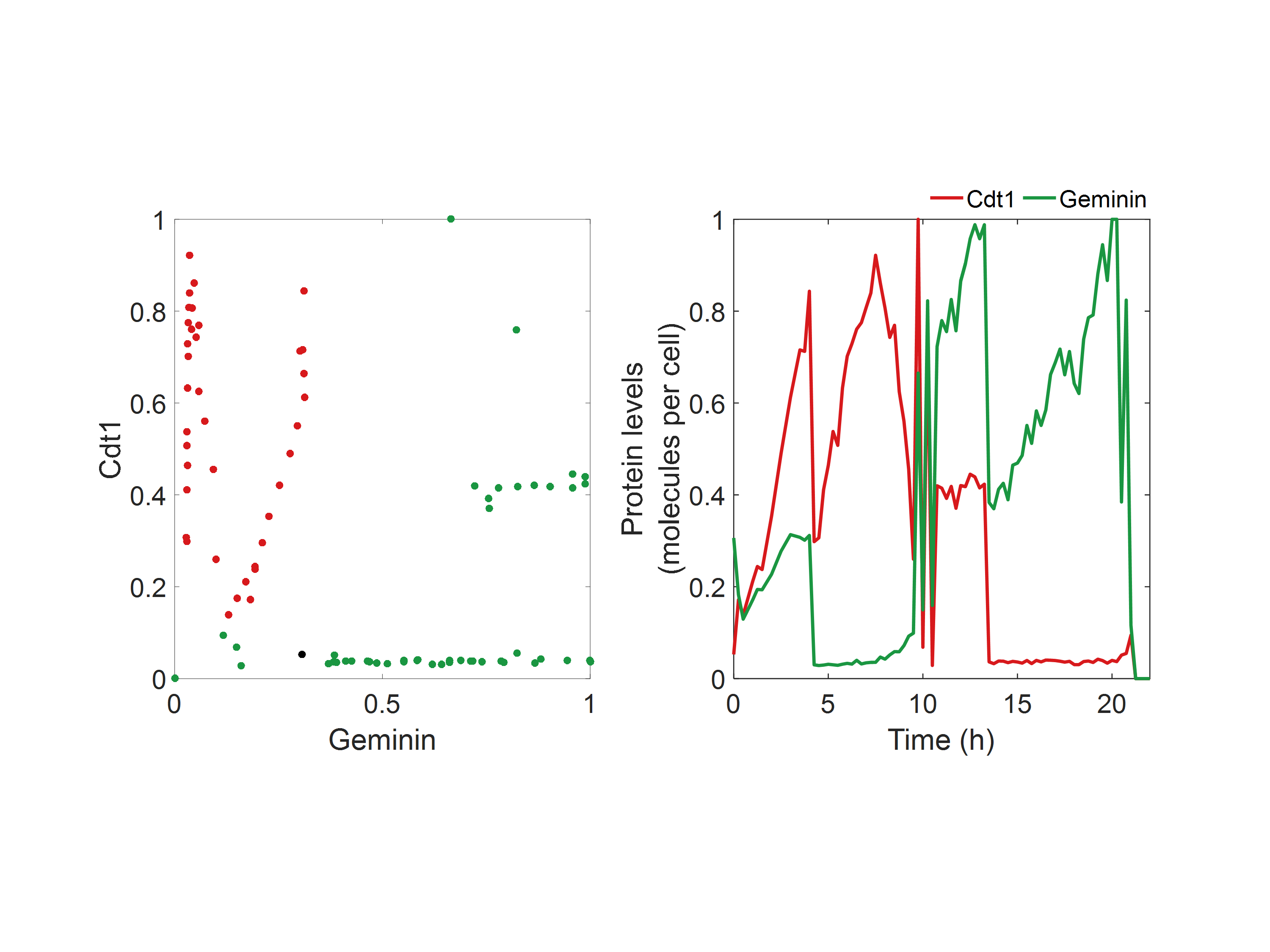

Supplement: S12 Fig — Phase portraits for case where qualitative pattern is different than in majority of cells, it is caused by high noise level. (TIF) [file pcbi.1007054.s012.TIF]

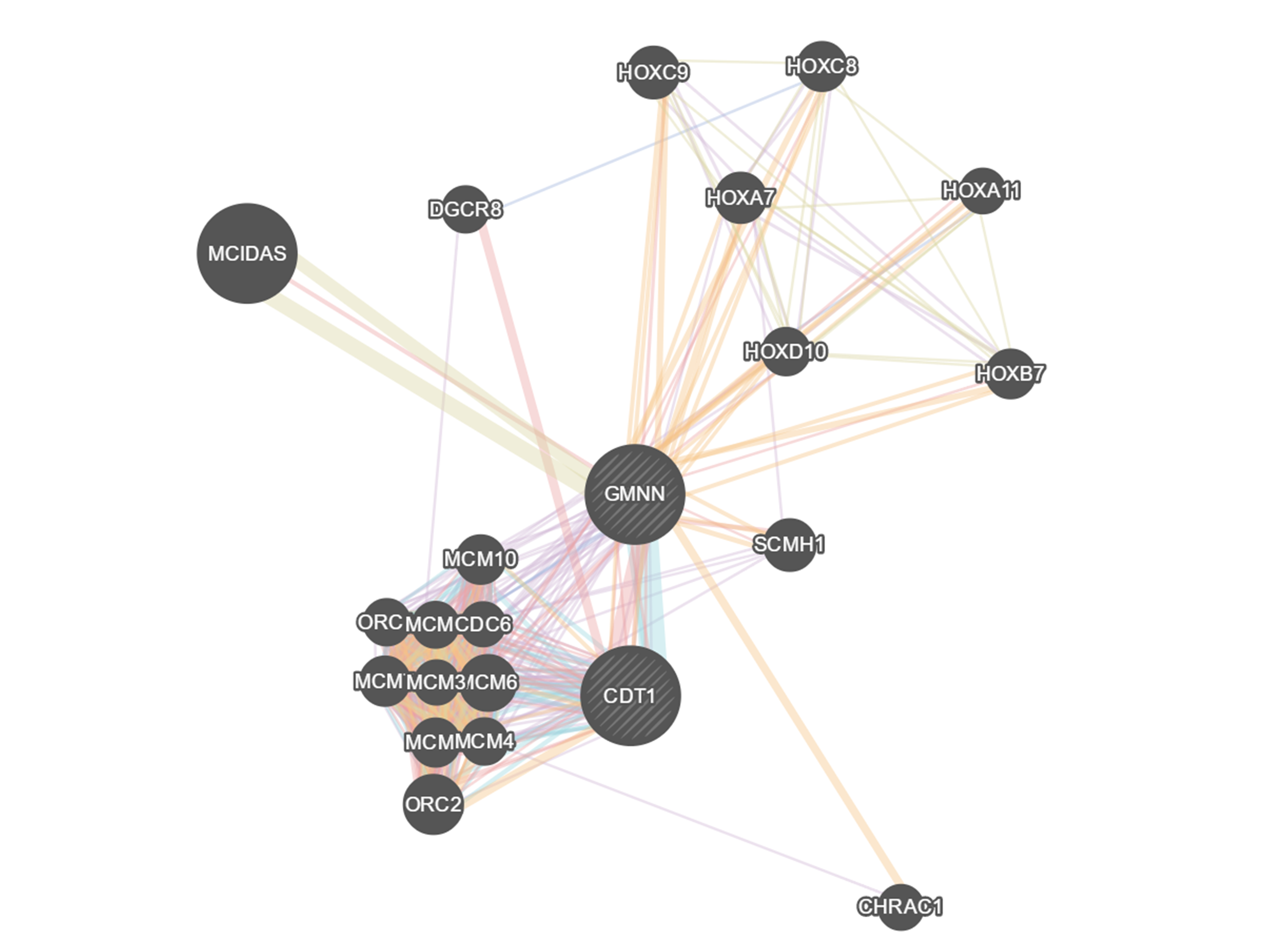

Supplement: S13 Fig — Cdt1 and its inhibitor Geminin are important regulators of replication licensing [60]. In normal cells, a critical balance between these two proteins ensures that firing of each origin along the genome will take place only once per cell cycle. In our case we measure expression of dysfunctional proteins, but regulated in the same way as original ones. Source: [61]. (TIF) [file pcbi.1007054.s013.tif]

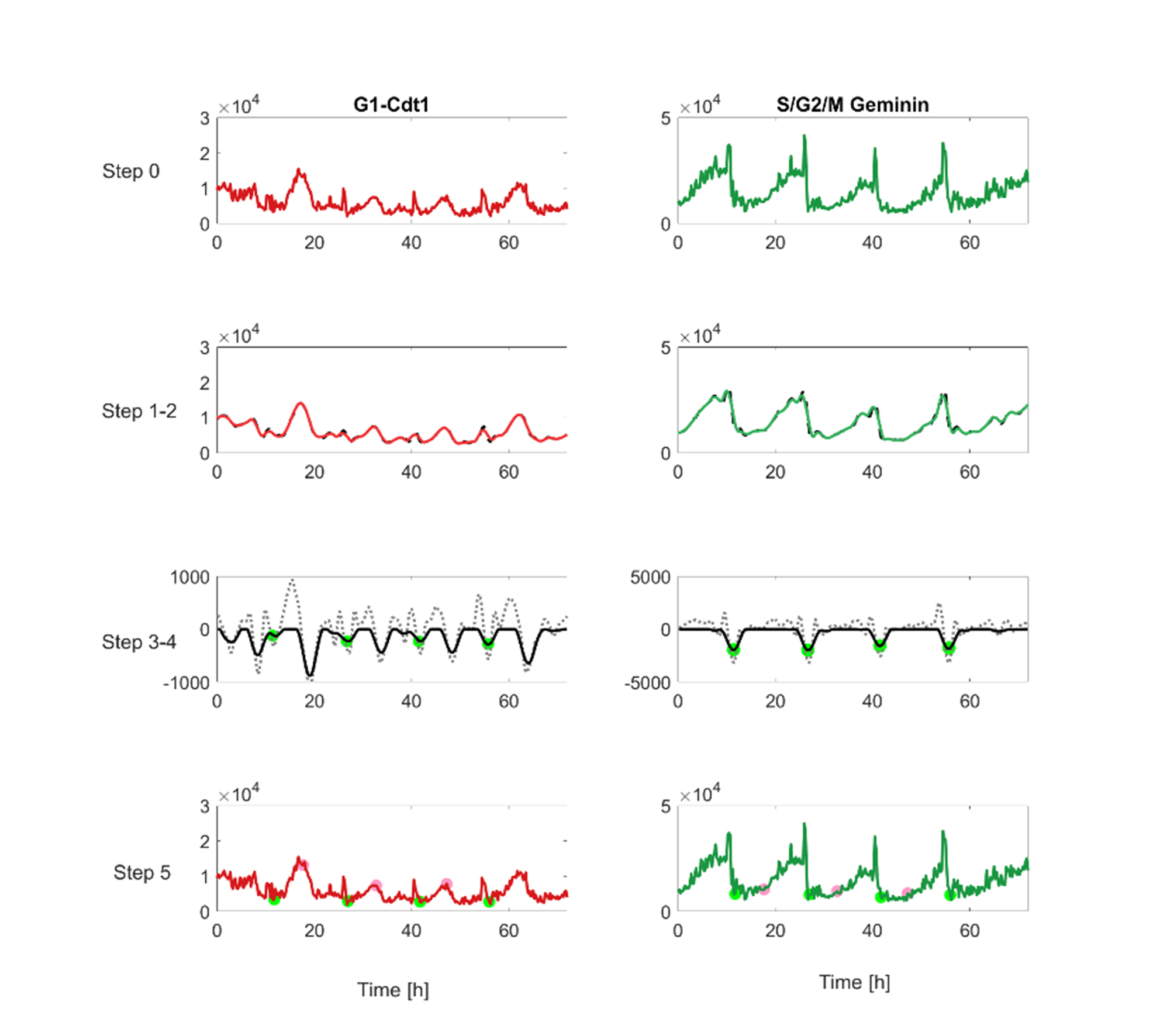

Supplement: S14 Fig — It includes several steps: (1–2) identification of the level of noise and determination of the appropriate parameter values for smoothing, (local regression using weighted linear least squares and a 2nd degree polynomial model); (3) numerical differentiation of Geminin protein levels; (4) detection of local minima of differentiated data to identify division moments, and (5) detection of Cdt1 protein maxima, the timing of which provides the estimated moment of transition from G1 to S phase of cell cycle (in this step we analyze only fragment of Cdt1 protein dynamic located between division moments). (TIF) [file pcbi.1007054.s014.tif]
