# Supplementary material for: Mathematical modelling reveals unexpected inheritance and variability patterns of cell cycle parameters in mammalian cells
Source: PLoS Comput Biol. 2019 Jun 3;15(6):e1007054. doi: 10.1371/journal.pcbi.1007054 (PMC6564046; doi:10.1371/journal.pcbi.1007054)
Supplement: S1 Text — The file contains additional results, discussion, description of methods and references. (DOCX) [file pcbi.1007054.s017.docx]

[Supplementary Results 2](#_Toc8146820)

[Description of data sets at our disposal 2](#_Toc8146821)

[Hereditary and environmental causes of cell cycle length variability based on the 15% FBS experiment 3](#_Toc8146822)

[Statistical dependence of cell cycle phase durations and cell cycle times 5](#_Toc8146823)

[Protein dynamics in individual and related cells 6](#_Toc8146824)

[Sensitivity of durations of cell cycle phases to serum stimulation 7](#_Toc8146825)

[Long-term simulations: Memory and transients 8](#_Toc8146826)

[Supplementary Discussion 9](#_Toc8146827)

[Modeling the multivariate statistics of the cell cycle and FUCCI protein dynamics 9](#_Toc8146828)

[Partitioning the variability of cell cycle time among successive cell cycle phases and the influence of lowering of mitogen level on this variability 10](#_Toc8146829)

[Influence of the circadian clock on correlations between cell cycle lengths of related cells 11](#_Toc8146830)

[Mechanisms of cell cycle phases regulation 12](#_Toc8146831)

[Supplementary Methods 14](#_Toc8146832)

[Cell lineage tracking 14](#_Toc8146833)

[Cell cycle characteristics 14](#_Toc8146834)

[Supplementary References 17](#_Toc8146835)

[Supplementary files 22](#_Toc8146836)

# Supplementary Results

## Description of data sets at our disposal

Various concentrations of serum have been used to modulate cell-cycle length and check how properties of individual cells change. Specifically, we investigated the influence of two different Fetal Bovine Serum (FBS) concentration on NIH3T3 cells using the FUCCI-2A system (Methods). We collected data from 123 cell lineages including 890 individual cells from eight recorded movies for 15% FBS, and 69 cell lineages including 224 individual cells from five recorded movies for 10% FBS. The distributions of the duration of the cell cycle, and of the G1 and the S/G2/M phases in cells maintained in 15% FBS show median values of 18.5 h, 8.25 h and 10.25 h (based on 649 measurements), respectively. Decreasing FBS to 10% increases the durations to 20.5 h, 9 h and 11.5 h (based on 106 measurements), respectively.

Pedigree analyses based on parent-progeny, sib-sib, grandparent-grandchild and cousin-cousin pairs, are based on the samples of, respectively, 183, 256, 54, and 49 cells in the 15% FBS experiment and on the samples of 19, 40, 7, and 5 cells in the 10% FBS experiment. A summary is presented in Table 1. For a comparison, at 5% FBS most of cells divided only once (43%) and identification of cell cycle duration was possible only for two cells. Pedigree analyses for this dataset was not performed.

**Table 1. Comparison of phases duration and sample sizes of the cell cycle, G1 phase, and combined S/G2/M phases for two serum concentrations.**

|  | **15 % FBS** | **10 % FBS** |
| --- | --- | --- |
| No. of cell lineages | 123 | 69 |
| No. of individual cells | 890 | 224 |
| No. of measured cell cycles | 649 | 106 |
| Median cell cycle length (hr)  Interquartile range | 18.5  (16.5 – 22.75) | 20.25  (18.44 – 22.75) |
| Median G1 phase length (hr)  Interquartile range | 8.25  (7 - 10.25) | 9  (7 -10.5) |
| Median S/G2/M phase length (hr)  Interquartile range | 10.25  (9-12.5) | 11.5  (10.5 – 12.81) |
| No. of measured parent-progeny pairs | 183 | 19 |
| No. of measured siblings pairs | 256 | 40 |
| No. of measured grandparent-grandchild pairs | 54 | 7 |
| No. of measured cousins pairs | 49 | 5 |
| No. of movies | 8 | 5 |

## Hereditary and environmental causes of cell cycle length variability based on the 15% FBS experiment

To address inheritance and relatedness, Pearson correlations of cell cycle durations between related cells were calculated. Correlation between parent and a randomly chosen progeny equals 0.4 (mean) ± 0.08 (standard deviation), based on 183 measurements; while correlation between a grandparent and a randomly chosen grand-progeny decreases to 0.03 ± 0.17, based on 54 measurements. Correlation between sibs equals 0.6 ± 0.04, based on 256 measurements. Estimation of standard deviations listed above is described in detail in the Supplementary Methods section. Correlation between cousins, based on one of the two cousin-cousin pairs from a pair of daughters (effectively using one-half of all possible cousin pairs), equals 0.3 ± 0.17, based on 49 measurements.

Consistent with the bifurcating autoregression paradigm, our results suggest that progeny cells inherit properties from their parents, as demonstrated by high correlation coefficient value, S3A Fig. Also consistently with this paradigm, we observe even stronger correlations between siblings. In the literature, such correlations are frequently explained by external factors affecting the cell cycle length, such as environmental conditions, among-cell communication, neighbourhood effect, or age of cells (see ref. [[1](#_ENREF_1)] for the NIH 3T3 cells). In our data, after two generations the correlation disappears, as exemplified by low correlation coefficients between grandparent and grand-progeny cells (S3A and S3B Fig), while the correlation between cousins remains high.

To obtain further insight into the cousin cell cycle times, we analyzed the dependence on the localization of cells in individual experiments. Our dataset is a series of 8 experiments. In each of them, conditions were constant, but the observation areas were selected randomly. Individual movies differ with respect to the initial and final number of cells, but other properties, such as population cell cycle lengths (S1 and S2 Fig) or correlations between cell cycle phases, do not vary. We focused on two movies with large sample sizes. Seventy-five percent of information about cousins has been obtained from these two experiments. The strong correlation between cousins is supported by the results from Movie 2 only (S3B Fig, Movie 1, ρ = 0.055 ± 0.23, 20 measurements; vs. Movie 2, ρ = 0.42 ± 0.25, 11 measurements).

We estimated, based on the MTrackJ [[2](#_ENREF_2)] files, the individual traces, defined as two-dimensional time trajectories of cousins (S3E Fig). Each color denotes one pair of cousins, a large dot indicates position of cell at the beginning of the cell cycle, and information about cell cycle duration is also included. The differences in cell cycle durations and distance among these cells were not correlated, which suggests that the physical proximity does not lead to similar pattern of cell cycle progression of the cousins. This is also shown in the zoomed insets of the figures, where cells located in the same area are characterized by a large variability in the cell cycle duration. To conclude, we cannot clearly explain the correlations between cousins; this effect may be attributed to several factors (e.g., time of the experiment, cell density and position). We have not included relations between cousins in our model.

We have tested the possible dependence between the cell cycle duration and cell’s birth time, the latter being an important potential confounding effect. Examination of the boxplots in S3D Fig does not support dependence of the cell cycle length on the birth time. Individual boxplots include measurements from two-hour windows of birth time. Median cell cycle length remains constant across the whole experiment. Using individual experiments, we have not found any clear influence of birth time on the cell cycle duration (S3C and S3D Fig), as well.

## Statistical dependence of cell cycle phase durations and cell cycle times

In the analyses that follow, all cells from 15% FBS experiments were pooled and treated as a single dataset. There is a strong positive correlation between durations of cell cycle phases and cell cycle (correlation coefficients *ρ_G1-cycle_* = 0.77, and *ρ_S/G2/M-cycle_* = 0.82 - S4A Fig). There is a low correlation between durations of G1 and S/G2/M phases cell-cycle length (*ρ_G1-SG2M_* = 0.33; S4C Fig).

The relationships between the total division time and the duration of the G1 and combined S/G2/M phases are presented in S4A Fig, in the form of scatterplots with linear regression lines. The minimum cell cycle time is 11.5 h, the minimum S/G2/M phase length is 5.5 h, and the minimum G1 phase length is 2.25 h. Analysis of the durations of particular phases as a proportion of the total cell cycle length has not clarified inheritance patterns (proportions of cell cycle phases are not passed on in a family). Cell cycle phase durations constitute approximately 45% (G1) and 55% (S/G2/M) of cell cycle length, nevertheless the distribution of the relative duration of either of these phases extends over 20-80% of cell cycle (S4B Fig). Variability in this proportion increases as the total cell cycle length increases (S4A Fig).

Normal cells without DNA damage, with an appropriate growth factor concentration in the environment, progress through the cell cycle without blocks, and divide within a short time. To distinguish these cells from those with extended cell cycle length we used a Gaussian mixture model and estimated the threshold for separation of the cell population into two groups to be equal to 23.3 h (S4D Fig). In this way we separated two subpopulations of slowly dividing cells (additionally separated into two subgroups using fuzzy c-means clustering [[3](#_ENREF_3)]) and fast dividing ones.

We focused on correlation between the durations of the cell cycle phases, which provides information about the regulatory mechanisms. In S4C Fig, three groups of cells are presents. In the first group, with “normal” cell cycle length (< 23.3 h, 77% of all cells), we observed no correlation between the phases of the cell cycle. In two subgroups with extended cell cycle length (> 23.3 h) we observed weak positive or negative correlation, which may be the consequence of either (1) reversible transition from G1 to G0, where the S/G2/M phases remain relatively short (10% of cells), or (2) reversible block of cells in the G2 phase (observed also in ref. [[4](#_ENREF_4)]), with the G1 phase remaining short (13% of cells).

In our data, correlation between the lengths of S/G2/M phases of the family members was higher than between the lengths of corresponding G1 phases (S4E Fig). This is not surprising, since it is in the S and M phases where DNA replication and cell division occur; accordingly, the whole S/G2/M phase varies less in duration. When we limit our observations to the G1 phase durations, except for siblings, the correlation between family members is very low (0.08-0.24). Previously described features such as: parent cell size, initial concentration of proteins, time when the cells were born, and other, do not have a significant impact on G1 progression process.

## Protein dynamics in individual and related cells

Correlation matrices that represent differences in protein dynamics in related and unrelated cells are presented in S5A Fig. X and Y axes represent the fraction of cell cycle progressed in cells compared (for example, X cell being parent and Y cell being progeny), with cell cycle lengths normalized ‘in-silico’ from 0 to 100 and, with the gaps between measurements linearly interpolated (). Correlations between protein expression at each time of cell cycle can be found using appropriate X and Y coordinates. Correlation matrices help identifying regions with similar protein levels between X- and Y-axis cells, such as for example, the diagonal area of the sib-sib correlation matrix with high correlations resulting from sibs’ relatedness.

We extended the analysis to the dynamics of the FUCCI markers to estimate the correlations in protein dynamics. We decided to focus on data from Movie 2/49 (see the Supporting Table: S_Data_15%_FBS_All_Cellsfor numbering of the films), which was recorded using a newer, more accurate microscope. S6 Fig includes a comparison of the Cdt1 and Geminin single-cell trajectories, obtained using the older (S6A Fig) and the newer (S6B Fig) microscope. As evident from this figure, the newer microscope allows to resolve very low-level readings, while the older one superimposes a significant layer of noise on the top of the signal.

Each cell in the population is described by seven parameters estimated from data with cell-dependent stochastic values. Scatterplots of the parameter values based on cell imaging as well as those based on model simulations are found in Supplementary Information (S11 Fig). Tables including corresponding numerical values of single cell parameters are available from the authors.Relations between these parameters are presented in the matrix in 2D Fig. (left).

## Sensitivity of durations of cell cycle phases to serum stimulation

Histograms comparing populations kept in different serum concentrations are presented in S7A Fig. Wilcoxon test confirmed that a lower concentration of FBS (10% FBS) lengthens the cell cycle time (from 18.75 to 20.25 h, Wilcoxon *p*-value 8.97×10^-04^). Unexpectedly, under both serum concentrations the distributions of G1 phase duration are similar (8.5 h to 9 h, Wilcoxon *p*-value 0.27) and the longer cell cycle duration in 10% FBS is mainly a consequence of the increase in S/G2/M phase length (from 10.25 h at 15% to 11.5 h at 10%, Wilcoxon test, *p*-value 4.77×10^-07^).

As a result, in cells cultured in 10% FBS (S7A Fig), G1 phase occupies a smaller portion of cell cycle duration (*p*-value = 0.019). S7D Fig presents the ratio of G1 phase length to the total cell cycle length for two populations (105 samples for 10% FBS, and the same number of randomly selected samples for 15% FBS).

The dynamics of cell cycle markers are presented in S7C Fig. Mean and median trends show that the maximum of the concentration of CDT1 protein is delayed in the 15% FBS condition. Correlations between cell cycle phase durations and the cell cycle time remain almost the same in both populations (S7B Fig).

In an attempt to measure the frequency of transition to quiescence, we extended analysis to incomplete cell cycles. Kaplan-Meier survival estimates are presented in S7E Fig. The main difference between the 10% and 15% FBS experiments is the fraction of the non-dividing cells, ie. cells that are likely to have irreversibly transitioned to the G0 phase or which extended their G1/G0 beyond the duration of the experiment. These cells constitute the majority of the 10% FBS population; see ref. [[5](#_ENREF_5)] for evidence of cell shuttling between G0 and G1. At 10% FBS concentration, usually cells divide only once within the 72 h experiment duration (average of 2 cells in the pedigree started by the initial parent cell, vs. 6 cells in the 15% FBS). In our observations we cannot distinguish between the G0 and G1 phases of the cell cycle.

## Long-term simulations: Memory and transients

In the first experiment, we track one single cell and its randomly chosen line of descent (in each division, a random progeny is selected) for several hundred divisions. Then, based on history of the line of descent, we analyze how cell cycle length was changing over generations. In S8A Fig we present histograms for two extreme cases, with low (13.6 h), and high (61.3 h), initial durations of the cell cycle corresponding to extreme values obtained *in vitro*. Following an initial transient, there is no difference in the medians or in shapes of distributions in these two cases. Initial cell cycle length does not influence long-term cell behavior, which is confirmed by no correlation (*ρ* = 0.035) between initial cell cycle length and median cell cycle length of 50 divisions presented in S8B Fig. This suggests that the “memory” of the initial condition disappears after few generations, in the sense that cell cycle length distribution returns to equilibrium, which is also confirmed by S9 Fig representing cell cycle duration across several generations. Additional figures representing changes in cell cycle durations for cells with extreme initial values of cell cycle length are presented in S8C Fig. Three colors represent different cell cycle lengths: *blue* for measurements below first quartile; *red* for measurements above third quartile, and *green* for measurements within the interquartile range. When the initial cell cycle length is *blue* or *red*, in subsequent generations the cell cycle length gradually returns to the equilibrium distribution.

In the second experiment, we check how cell count in the population changes during 200 h of observation. In S8D Fig we present histograms for two extreme cases, with the initial values of cell cycle low (13.6 h), and high (61.3 h). Despite the eventual return of the cell cycle times to the stationary pattern, after 200 h the population initiated by a cell with a short (13.6 h) cycle is 40 times larger than that initiated by a cell with a long (61.3 h) cycle (S8F Fig). Scatterplots in S8E Fig demonstrate that in general, the number of cells after 200 h is strongly negatively correlated (*ρ* = -0.65) with the cell cycle length of ancestor.

# Supplementary Discussion

## Modeling the multivariate statistics of the cell cycle and FUCCI protein dynamics

Strong correlations among family members, although reproduced by the model, are difficult to trace to a well-defined cause. Among other, we investigated the potential influence of physical distance of cousin cells on cousin-cousin correlation of cell cycle times (S3E Fig). Plots in S10 Fig demonstrate that no such dependence exists. There is no dependence of the cell cycle length of the birthdate of cells, as well (S3D Fig). A possible explanation is the transmission of information beyond DNA, viz. epigenetic regulation [[6](#_ENREF_6), [7](#_ENREF_7)], which includes DNA and histone modifications, histone variants, non-histone chromatin proteins, nuclear RNA as well as higher-order chromatin organization. In our case cell cycle duration, protein dynamics and phase duration show strong dependence on the initial concentration of proteins.

Cell cycle progression is controlled by a complex network of regulatory proteins, called cyclin-dependent kinases, and their activators and inhibitors. Progress in biological experiments and mathematical modeling has led to an increase of knowledge of these precise regulatory mechanisms [[8](#_ENREF_8), [9](#_ENREF_9)]. Cell cycle duration in a single cell is the result of deterministic network dynamics, stochastic noise and epigenetic regulation. Sources of stochasticity are the extrinsic and intrinsic noise, well characterized for many cell types (e.g. [[10](#_ENREF_10), [11](#_ENREF_11)]). Non-genetic intrinsic heterogeneity stems from the random (thermal) nature of the interaction of individual molecules, such as mRNA and proteins. Since some of these biomolecules are present in relatively small numbers in a cell, their stochastic fluctuations are, unlike in the classical test-tube chemistry, not averaged out [[12](#_ENREF_12)]. As stated above, an equally important source of heterogeneity is the unequal partitioning of cellular mRNA and proteins between two progeny cells after cell division. The importance of including these factors in cell cycle models has been widely analyzed [[13](#_ENREF_13), [14](#_ENREF_14)].

## Partitioning the variability of cell cycle time among successive cell cycle phases and the influence of lowering of mitogen level on this variability

It is frequently assumed that the main source of the variability of cell cycle duration is the G1 phase, in which the cell synthesizes mRNA and proteins before the decision about the transition to the S phase is made [[15](#_ENREF_15)]. G1 phase duration depends on external signals, such as the extracellular growth factors, as well as on the cell size. That is why it has been commonly assumed that the G1 phase duration is stochastic, while the S/G2/M phases joint duration is approximately constant. In the most extreme form, this view has been expressed by the Smith-Martin model of the cell cycle [[15](#_ENREF_15)]. In our data a similar relationship has been observed under stable conditions: normalized median absolute deviations (MAD) for G1 and S/G2/M phases were equal to 0.27 and 0.235, respectively, and coefficients of variations (CV) were equal to 0.41 and 0.35, respectively. However, the difference between the variability of G1 and that of the joint duration of S/G2/M is not dramatic. This is consistent with variability in all phases of cell cycle having been reported in many studies. Darzynkiewicz et al. [[16](#_ENREF_16)] analyzed Chinese hamster ovary (CHO) cells using flow cytometry. They reported variability in G1 phase caused mainly by unequal division of cytoplasmic constituents into progeny cells and the main conclusion was that that the cell cycle heterogeneity is generated mostly during cytokinesis and to a lesser degree during G2 phase. These data influenced models of Kimmel et al. [[17](#_ENREF_17)] and Arino and Kimmel [[18](#_ENREF_18)]. These models may be considered precursors of the model in the present paper. Later on Kroll et al. [[19](#_ENREF_19)] used flow cytometric methods based on bromodeoxyuridine (BrdU) to discover variability of S phase durations in cells. Mathematical models and experimental methods based on BrdU assays were used to test the hypothesis about coupling of the S and G2/M phases (eg. [[20](#_ENREF_20)]), as well as to estimate distributions of individual phases of the cell cycle (S phase in Larsson et al. [[21](#_ENREF_21)] and G2 phase in [[22](#_ENREF_22)]). Recently, more complex models including variability in all cell cycle phases were proposed [[23](#_ENREF_23)]. While older methods allowed observing specific cell properties mostly on cell population level, discovery of fluorescent proteins fused with proteins of interest at DNA level and stably expressed, provided an effective way to measure cell-cycle dynamics in single cells. Araujo [[24](#_ENREF_24)] published a system consisting of cells markers for each phase of cell cycle applied to MCF10A (epithelial mammary), RPE (retinal pigment epithelium), and HeLa cells. Analysis of G1, S, and G2 phase times shows that their distributions have high (normalized) MADs, and CVs, and they are strongly correlated with cell cycle length. The distribution of the M phase duration is tight, with little variability, and it seems independent of the cell cycle length.

Overton et al. [[5](#_ENREF_5)] found that in human MCF10A cells, serum starvation does not lead to lengthening of the cell cycle, but only to increase of the rate of transition to quiescence. In contrast to ref. [[5](#_ENREF_5)], in our case the growth factors (FBS) also influence the durations of G1 and S/G2/M, with the duration of S/G2/M being affected to a larger extent (Table I, rows 4-6). A similar effect has been identified as early as 50 years ago by Watanabe [[25](#_ENREF_25)]. This finding suggests that the mitogen also stimulates cell cycle checkpoints outside the G1 to S transition. Foijer and de Riele [[26](#_ENREF_26)] confirm that in the absence of mitogens another mechanism restricts proliferation arrest in the G2 phase. Increased concentrations of FBS speed up the cell cycle and stimulate more cells to leave the G0 phase. However, within the range of FBS concentrations tested, the overall dynamics of proteins, and correlations between cell pedigree members remain apparently unchanged, so that our model, with modified parameters, still applies.

Our data suggest that on one hand the S or the G2 phase are more sensitive to serum deprivation, but on the other, significantly more cells remain in G0 phase under this condition. Other researchers found that despite the fact that different times of initiation of replication in the S phase are observed, and the biological significance of replication timing has remained unclear [[27](#_ENREF_27)], the overall length of the mammalian S-phase is remarkably resilient [[28](#_ENREF_28), [29](#_ENREF_29)]. Even with a significant change in growing conditions, such as in the presence of adenine and uridine (AU), the doubling time and the S phase duration remained unchanged [[30](#_ENREF_30)]. Still, the role of cell-to-cell heterogeneity of the S phase remains an open question.

## Influence of the circadian clock on correlations between cell cycle lengths of related cells

Strong positive correlation between cell cycle duration of siblings, diluted over consecutive divisions, was observed in other cell types [[31](#_ENREF_31), [32](#_ENREF_32)], with similarities between cousins as reported recently by Sandler [[31](#_ENREF_31)] and collaborators. They speculated that the impact of the circadian clock on the cell cycle duration might underlay this data. However, they used L1210 lymphoblasts with relatively short cell cycle length of 8 to 10 hours, so during two consecutive divisions of cells the phases of circadian clock are potentially extremely different, thus challenging the potential the role of the clock in such fast dividing cells. We observed substantial between-cousin correlations only in one experiment, which is particularly interesting and consistent with contradictory information about division times in pedigrees reported elsewhere. An example is negative and positive correlation between parent and progeny in the same cell line, as shown comparing ref. [[33](#_ENREF_33)] with our results; similar differences were observed in EMT6 cell line [[34](#_ENREF_34)]).

Some of the present authors (Feuillet and Delaunay) have investigated the circadian clock-cell cycle connection by quantifying the dynamics of the two oscillators in real time, in single live mammalian cells We used the circadian clock reporter REV-ERBα::VENUS [[35](#_ENREF_35)]. We also added the FUCCI-2A system (modified from the one from [[36](#_ENREF_36)]) to follow cell cycle progression. Mathematical analysis and stochastic modelling showed that coupling governed cell cycle and circadian interaction in NIH3T3 cells [[37](#_ENREF_37)]. It also revealed that the clock reporter reproducibly peaked about 5 hours after cell division (phase locking). Changing cell cycle duration impacted the circadian cycles, but 1:1 locking was resilient to such changes. In the current paper, we do not follow the clock-cell cycle coupling and the data we use have been obtained in absence of external cues.

## Mechanisms of cell cycle phases regulation

Based on presented results we decided to test two hypotheses, the first one assumed that the cell cycle length is inherited from the parent, and then phases of that cell cycle take respectively 45% and 55% up to a noise term. This theory was rejected because we could not reproduce experimental data. In view of our data, more likely is the second hypothesis that the phases are independent processes, so that elongations of G1 or G2 phases do not affect other phases of the cell cycle. In contrast, in pluripotent cells, interesting relations between G1 phase and cell fate decisions were discovered [[38](#_ENREF_38)]; it may be interesting to test if these relations are lost after cell differentiation.

# Supplementary Methods

## Cell lineage tracking

Sequences of images were analyzed using the LineageTracker software [[39](#_ENREF_39)]. This program semi-automatically identifies division times and marks related cells. In most cases, nucleus boundaries are identified automatically except for a few frames before and after cell division, in which it is difficult due to low fluorescence level of marker proteins during mitosis. Manual corrections and verifications of automatic detection of lineages were carried out by the biologist (CF). Data include the fluorescence level of Cdt1 and Geminin proteins and lineage relations between cells. The tracking times for individual cells vary; the reason is that only in some cases cells were observed from the beginning of the experiment, since most of them were born after observation started. Following division, one randomly selected progeny cell retains the label of the parent cell; the other progeny obtains a new label.

## Cell cycle characteristics

We characterized cellular populations based on complete cell cycle lengths (if the cell divided at least twice during the experiment) and incomplete cell cycles (first division before the experiment started or the last division after the experiment ended). Differences between cell cycle lengths were quantified using Kaplan-Meier survival curves [[40](#_ENREF_40)] and histograms. Logrank test was used to compare the Kaplan-Meier curves of two samples. All histograms were normalized, so that the heights of all bars (probabilities of selecting an observation within the corresponding bins) add up to 1. Times of the G1 and S/G2/M phases were estimated using the method described earlier; their distributions were visualized using histograms, and Wilcoxon rank sum test was used to verify if cell populations grown in different serum concentration have different properties.

To compare the protein dynamics between two experiments, data on fluorescence levels for the two proteins (complete cell cycles only) were collected. The cell cycle duration was normalized to 0 – 100%, with 1% step, missing measurements were interpolated, and the fluorescence levels were normalized to the maximum fluorescence. Normalization procedure was applied to eliminate the stochastic effects of plasmid transfection. Wilcoxon rank sum test was used to check if the G1 phase time was the same fraction of the cell cycle time in cells with lower and higher dose of serum.

To address inheritance and relatedness, Pearson correlations of inheritance pattern cycle and G1 and S/G2/M phases durations between cells from one lineage were calculated. To calculate prediction errors for progeny cells and cousin cells, we randomly selected half of the measurements, for parent and progeny we randomly chose one progeny cell; the same method was used to select the grand-progeny cells. The simulations were repeated 10,000 times. All plots with error bars as well as correlations with ± signs, represent standard deviations.

The differences of protein expression in cellular population are often explained by cell size [[41](#_ENREF_41), [42](#_ENREF_42)]. We were not able to analyse the cell size, because both markers used (Cdt1 and Geminin) are localized in the nucleus, but we compared the nucleus sizes among family members, the impact of nucleus size on proliferation properties, and other features. Size of the nucleus was calculated using total and mean fluorescence levels from the LineageTracker.

To analyse positions of individual cells, the MTrackJ [[2](#_ENREF_2)] files were used. They include information about the positions of clusters of cells; using the division times we reconstructed probable spatial relations between cells. In all cases we tested normality using the Lilliefors test [[43](#_ENREF_43)].

Degradation of the marker proteins (Cdt1 and Geminin) in the M phase of the cell cycle is creating problems with pinpointing the division times and consequently with estimation of protein distribution between progeny cells. We have tested several possible protein distributions from parent to progeny cells. Our simulation analysis shows that even large disparities in initial number do not cause dysregulation of the population.

Long-term simulations were based on the dataset created by our model. We started simulation with a single ancestor cell with parameters based on experimental data representing mean values for population. After 400 h of simulated time, we chose 1000 cells out of the resulting population .

Phase portraits were used to visualize the behaviour of the system in state space (Fig. 4B). Each point of the two-dimensional phase portrait represents a pair of variables characterising the system’s state at a given time. In our case, we used Cdt1 and Geminin normalized fluorescence intensities to examine cell cycle dynamics in individual cells. Resulting image shows a clear feature of the system dynamics; for various initial conditions the cell cycle lengths may be different, but cell cycle progression remains similar.

# Supplementary References

1. DiSalvo CV, Zhang D, Jacobberger JW. Regulation of NIH-3T3 cell G1 phase transit by serum during exponential growth. Cell proliferation. 1995;28(9):511-24. PubMed PMID: 7578600.

2. Meijering E, Dzyubachyk O, Smal I. Methods for cell and particle tracking. Methods in enzymology. 2012;504:183-200. doi: 10.1016/B978-0-12-391857-4.00009-4. PubMed PMID: 22264535.

3. Bezdek JC, Ehrlich R, Full W. FCM: The fuzzy c-means clustering algorithm. Computers & Geosciences. 1984;10(2-3):191-203. doi: 10.1016/0098-3004(84)90020-7.

4. Gire V, Dulic V. Senescence from G2 arrest, revisited. Cell cycle. 2015;14(3):297-304. doi: 10.1080/15384101.2014.1000134. PubMed PMID: 25564883; PubMed Central PMCID: PMC4353294.

5. Overton KW, Spencer SL, Noderer WL, Meyer T, Wang CL. Basal p21 controls population heterogeneity in cycling and quiescent cell cycle states. Proceedings of the National Academy of Sciences of the United States of America. 2014;111(41):E4386-93. doi: 10.1073/pnas.1409797111. PubMed PMID: 25267623; PubMed Central PMCID: PMC4205626.

6. Probst AV, Dunleavy E, Almouzni G. Epigenetic inheritance during the cell cycle. Nature reviews Molecular cell biology. 2009;10(3):192-206. doi: 10.1038/nrm2640. PubMed PMID: 19234478.

7. Heard E, Martienssen RA. Transgenerational epigenetic inheritance: myths and mechanisms. Cell. 2014;157(1):95-109. doi: 10.1016/j.cell.2014.02.045. PubMed PMID: 24679529; PubMed Central PMCID: PMC4020004.

8. Tyson JJ, Novak B. Models in biology: lessons from modeling regulation of the eukaryotic cell cycle. BMC biology. 2015;13:46. doi: 10.1186/s12915-015-0158-9. PubMed PMID: 26129844; PubMed Central PMCID: PMC4486427.

9. Barr AR, Heldt FS, Zhang T, Bakal C, Novak B. A Dynamical Framework for the All-or-None G1/S Transition. Cell systems. 2016;2(1):27-37. doi: 10.1016/j.cels.2016.01.001. PubMed PMID: 27136687; PubMed Central PMCID: PMC4802413.

10. Altschuler SJ, Wu LF. Cellular heterogeneity: do differences make a difference? Cell. 2010;141(4):559-63. doi: 10.1016/j.cell.2010.04.033. PubMed PMID: 20478246; PubMed Central PMCID: PMC2918286.

11. Elowitz MB, Levine AJ, Siggia ED, Swain PS. Stochastic gene expression in a single cell. Science. 2002;297(5584):1183-6. doi: 10.1126/science.1070919. PubMed PMID: 12183631.

12. Huang S. Non-genetic heterogeneity of cells in development: more than just noise. Development. 2009;136(23):3853-62. doi: 10.1242/dev.035139. PubMed PMID: 19906852; PubMed Central PMCID: PMC2778736.

13. Kar S, Baumann WT, Paul MR, Tyson JJ. Exploring the roles of noise in the eukaryotic cell cycle. Proceedings of the National Academy of Sciences of the United States of America. 2009;106(16):6471-6. doi: 10.1073/pnas.0810034106. PubMed PMID: 19246388; PubMed Central PMCID: PMC2672517.

14. Huh D, Paulsson J. Non-genetic heterogeneity from stochastic partitioning at cell division. Nature genetics. 2011;43(2):95-100. doi: 10.1038/ng.729. PubMed PMID: 21186354; PubMed Central PMCID: PMC3208402.

15. Smith JA, Martin L. Do cells cycle? Proceedings of the National Academy of Sciences of the United States of America. 1973;70(4):1263-7. PubMed PMID: 4515625; PubMed Central PMCID: PMC433472.

16. Darzynkiewicz Z, Crissman H, Traganos F, Steinkamp J. Cell heterogeneity during the cell cycle. Journal of cellular physiology. 1982;113(3):465-74. doi: 10.1002/jcp.1041130316. PubMed PMID: 6184378.

17. Kimmel M, Darzynkiewicz Z, Arino O, Traganos F. Analysis of a cell cycle model based on unequal division of metabolic constituents to daughter cells during cytokinesis. Journal of theoretical biology. 1984;110(4):637-64. PubMed PMID: 6084152.

18. Arino OK, M. Asymptotic analysis of a cell cycle model based on unequal division. SIAM Journal on Applied Mathematics. 1987;47(1):128-45.

19. Kroll S, Char D, Kaleta-Michaels S. A stochastic model for dual label experiments: an analysis of the heterogeneity of S phase duration. Cell proliferation. 1995;28(10):545-67. PubMed PMID: 7488674.

20. Bertuzzi A, Faretta M, Gandolfi A, Sinisgalli C, Starace G, Valoti G, et al. Kinetic heterogeneity of an experimental tumour revealed by BrdUrd incorporation and mathematical modelling. Bulletin of mathematical biology. 2002;64(2):355-84. doi: 10.1006/bulm.2001.0280. PubMed PMID: 11926121.

21. Larsson S, Ryden T, Holst U, Oredsson S, Johansson M. Estimating the variation in S phase duration from flow cytometric histograms. Mathematical biosciences. 2008;213(1):40-9. doi: 10.1016/j.mbs.2008.01.009. PubMed PMID: 18433802.

22. Larsson S, Ryden T, Holst U, Oredsson S, Johansson M. Estimating the distribution of the G(2) phase duration from flow cytometric histograms. Mathematical biosciences. 2008;211(1):1-17. doi: 10.1016/j.mbs.2007.08.009. PubMed PMID: 17942127.

23. Weber TS, Jaehnert I, Schichor C, Or-Guil M, Carneiro J. Quantifying the length and variance of the eukaryotic cell cycle phases by a stochastic model and dual nucleoside pulse labelling. PLoS computational biology. 2014;10(7):e1003616. doi: 10.1371/journal.pcbi.1003616. PubMed PMID: 25058870; PubMed Central PMCID: PMC4109856.

24. Araujo AR, Gelens L, Sheriff RS, Santos SD. Positive Feedback Keeps Duration of Mitosis Temporally Insulated from Upstream Cell-Cycle Events. Mol Cell. 2016;64(2):362-75. doi: 10.1016/j.molcel.2016.09.018. PubMed PMID: 27768873; PubMed Central PMCID: PMC5077699.

25. Watanabe I, Okada S. Stationary phase of cultured mammalian cells (L5178Y). The Journal of cell biology. 1967;35(2):285-94. PubMed PMID: 6055989; PubMed Central PMCID: PMC2107128.

26. Foijer F, Te Riele H. Restriction beyond the restriction point: mitogen requirement for G2 passage. Cell division. 2006;1:8. doi: 10.1186/1747-1028-1-8. PubMed PMID: 16759363; PubMed Central PMCID: PMC1481568.

27. Rhind N, Gilbert DM. DNA replication timing. Cold Spring Harbor perspectives in biology. 2013;5(8):a010132. doi: 10.1101/cshperspect.a010132. PubMed PMID: 23838440; PubMed Central PMCID: PMC3721284.

28. Behaegel J, Comet JP, Bernot G, Cornillon E, Delaunay F. A hybrid model of cell cycle in mammals. Journal of bioinformatics and computational biology. 2016;14(1):1640001. doi: 10.1142/S0219720016400011. PubMed PMID: 26708052.

29. Gilbert DM. Replication origin plasticity, Taylor-made: inhibition vs recruitment of origins under conditions of replication stress. Chromosoma. 2007;116(4):341-7. doi: 10.1007/s00412-007-0105-9. PubMed PMID: 17404750.

30. Anglana M, Apiou F, Bensimon A, Debatisse M. Dynamics of DNA replication in mammalian somatic cells: nucleotide pool modulates origin choice and interorigin spacing. Cell. 2003;114(3):385-94. PubMed PMID: 12914702.

31. Sandler O, Mizrahi SP, Weiss N, Agam O, Simon I, Balaban NQ. Lineage correlations of single cell division time as a probe of cell-cycle dynamics. Nature. 2015;519(7544):468-71. doi: 10.1038/nature14318. PubMed PMID: 25762143.

32. Hawkins ED, Markham JF, McGuinness LP, Hodgkin PD. A single-cell pedigree analysis of alternative stochastic lymphocyte fates. Proceedings of the National Academy of Sciences of the United States of America. 2009;106(32):13457-62. doi: 10.1073/pnas.0905629106. PubMed PMID: 19633185; PubMed Central PMCID: PMC2715326.

33. Staudte RG, Huggins RM, Zhang J, Axelrod DE, Kimmel M. Estimating clonal heterogeneity and interexperiment variability with the bifurcating autoregressive model for cell lineage data. Mathematical biosciences. 1997;143(2):103-21. PubMed PMID: 9212596.

34. d'Hooghe MC, Hemon D, Valleron AJ, Malaise EP. Comparative effects of ionizing radiations on cycle time and mitotic duration. A time-lapse cinematography study. Radiation research. 1980;81(3):384-92. PubMed PMID: 7360890.

35. Nagoshi E, Saini C, Bauer C, Laroche T, Naef F, Schibler U. Circadian gene expression in individual fibroblasts: cell-autonomous and self-sustained oscillators pass time to daughter cells. Cell. 2004;119(5):693-705. doi: 10.1016/j.cell.2004.11.015. PubMed PMID: 15550250.

36. Sakaue-Sawano A, Miyawaki A. Visualizing spatiotemporal dynamics of multicellular cell-cycle progressions with fucci technology. Cold Spring Harbor protocols. 2014;2014(5). doi: 10.1101/pdb.prot080408. PubMed PMID: 24786503.

37. Feillet C, Krusche P, Tamanini F, Janssens RC, Downey MJ, Martin P, et al. Phase locking and multiple oscillating attractors for the coupled mammalian clock and cell cycle. Proceedings of the National Academy of Sciences of the United States of America. 2014;111(27):9828-33. doi: 10.1073/pnas.1320474111. PubMed PMID: 24958884; PubMed Central PMCID: PMC4103330.

38. Dalton S. G1 compartmentalization and cell fate coordination. Cell. 2013;155(1):13-4. doi: 10.1016/j.cell.2013.09.015. PubMed PMID: 24074854.

39. Downey MJ, Jeziorska DM, Ott S, Tamai TK, Koentges G, Vance KW, et al. Extracting fluorescent reporter time courses of cell lineages from high-throughput microscopy at low temporal resolution. PloS one. 2011;6(12):e27886. doi: 10.1371/journal.pone.0027886. PubMed PMID: 22194797; PubMed Central PMCID: PMC3240619.

40. Cox DR, Oakes D. Analysis of survival data. London ; New York: Chapman and Hall; 1984. viii, 201 p. p.

41. Shields R, Brooks RF, Riddle PN, Capellaro DF, Delia D. Cell size, cell cycle and transition probability in mouse fibroblasts. Cell. 1978;15(2):469-74. PubMed PMID: 569024.

42. Wells WA. Does size matter? The Journal of cell biology. 2002;158(7):1156-9. PubMed PMID: 12356860; PubMed Central PMCID: PMC2173227.

43. Ghasemi A, Zahediasl S. Normality tests for statistical analysis: a guide for non-statisticians. International journal of endocrinology and metabolism. 2012;10(2):486-9. doi: 10.5812/ijem.3505. PubMed PMID: 23843808; PubMed Central PMCID: PMC3693611.

44. De Marco V, Gillespie PJ, Li A, Karantzelis N, Christodoulou E, Klompmaker R, et al. Quaternary structure of the human Cdt1-Geminin complex regulates DNA replication licensing. Proceedings of the National Academy of Sciences of the United States of America. 2009;106(47):19807-12. doi: 10.1073/pnas.0905281106. PubMed PMID: 19906994; PubMed Central PMCID: PMC2775996.

45. Franz M, Rodriguez H, Lopes C, Zuberi K, Montojo J, Bader GD, et al. GeneMANIA update 2018. Nucleic acids research. 2018;46(W1):W60-W4. doi: 10.1093/nar/gky311. PubMed PMID: 29912392; PubMed Central PMCID: PMC6030815.

# Supplementary files

**S1 Fig** **Comparison of repetitions of the same experiment (15% FBS)**. Boxplots represent cell-cycle length. During observation, randomly selected area is recorded during 72 hours. Movies differ with respect to initial number of cells and their location.

**S2 Fig** **Comparison of repeats of the same experiment (15% FBS)**. The correlation between: 1) cell cycle and G1 phase, 2) cell cycle and S/G2/M phases and 3) G1 and S/G2/M phases, 4) sample sizes.

**S3 Fig Family relations and differences between experiments**

1. Correlations between family members based on experimental data. Estimation of standard deviations is described in detail in Methods.
2. Correlations of cell cycle length between family members for two selected movies. 75% of information about cousins came from these movies. Strong correlation between cousins is specific for case 2.
3. Verification of the hypothesis that cell-cycle duration depends on the birth date of the cell. Cells’ birth dates rounded to the nearest multiplicity of 2 hours are presented as boxplots to address the hypothesis.
4. Cross-plot of cells’ birth date and the cell-cycle length for cells from two selected movies.
5. Individual traces for cousins. Each color denotes one pair of cousins; a large dot indicates position of cells at the beginning of the cell cycle; information about cell-cycle duration is also included.

**S4 Fig Relationships between durations of the cell cycle and the G1 and S/G2/M phases**

1. Experimental data. Linear relationship between the total division time and the duration of phases. Solid black lines show the fitted linear relations of the form *y* = (*slope*)×*x*.
2. Linear relations presented in the histograms, where the distribution of proportionality is shown. In 80% of samples, G1 phase occupies 35-55% of the cell cycle.
3. Cross-plot of the times of G1 and S/G2/M cell-cycle phases. *Blue*, “normal” cells; *green*, extended cell- cycle-length cells with longer S/G2/M phases; and *red*, extended cell-cycle-length cells with longer G1 phase.
4. Gaussian mixture model distinguishing the “normal” from extended cell-cycle-length cells, combined with the EM (Expectation Maximization) algorithm, to estimate the threshold (22 h) for separation of cells into two groups.
5. Correlation between phases and cell-cycle lengths for family members..

**S5 Fig Correlation matrices represent changes in protein dynamics in related and unrelated cells.** X and Y axes represent fractions (0 to 1) of cell cycle progressed, with the gaps between measurements normalized to 0.01. Correlations between protein expressions at each time of cell cycle are found using corresponding coordinates. Correlation matrices can help finding parts of the cell cycle with similar dynamics, as it is shown in the diagonal area of the progeny-progeny matrix.

**S6 Fig** **Time trajectories of Cdt1 and Geminin levels in single cells, and mean and median trajectories**. Two cases. :

1. Movies 41-48 – 472 measurements
2. Movie 49 – 177 measurements

**S7 Fig** **Populations with different growth factors concentrations**

1. Comparison of cell cycle, G1 phase and combined S/G2/M phases durations for two serum (FBS) concentrations. Calculations were performed based on 105 and 642 measurements for 10% and 15% of FBS, respectively. Histograms were normalized, the height of each bar is equal to the probability of selecting an observation within the corresponding bin interval, and the height of all of the bars sums up to 1. All distributions have characteristic lognormal-like shape; additional information about medians are included directly on the plots. Lower dose of serum causes extension of G1 and S/G2/M phases and as a consequence of the whole cell-cycle length. Changes in the lengths of the cell cycle and of the S/G2/M phases are statistically significant (Wilcoxon rank sum test).
2. Pearson rank correlations between lengths of the phases and the cell cycle. Standard deviations were calculated using Monte Carlo cross validation and 10,000 iterations.
3. Comparison between protein dynamics. Each line denotes one cell, black solid line is mean trace, black dashed line is median trace. The division moments were selected using procedure described in Methods section.
4. G1 phase takes proportionally less time under 10% FBS (Wilcoxon rank sum test), as it is shown in the boxplot (105 samples for 10% FBS, and the same number of randomly selected samples for 15% FBS).
5. Survival function mapping division events onto time, based on cells that divided at least twice during the experiment, so the time of birth and death could be estimated. Kaplan-Meier curve maps division events onto time, including cells which divided only once, so that either their birth or death are not known. Significant difference between 10% FBS and 15% FBS is observed only when incomplete cell cycles are included.

**S8 Fig Results of long-term behavior predicted by the model**

1. Histograms of cell-cycle lengths for a single ancestor cell and its progeny. After each of 4,000 divisions along a single line of descent, one randomly chosen progeny was used for analysis. *Blue* and *red* color represent cases with low (13.6 h) and high (61.3 h) initial cell-cycle length, respectively. The medians in both cases are similar (21.9 h and 21.8 h).
2. The scatter plot of initial cell-cycle length and median cell-cycle length after 400 generations. No correlation is observed is significant statistically (*ρ* = -0.04).
3. Heat maps representing changes in cell-cycle durations in next generations. Three colors represent different cell-cycle lengths: *blue* for measurements below the first quartile; *red* for measurements above third quartile, and *green* for measurements within the interquartile range.
4. Histograms of cell-cycle lengths for a population started from a single ancestor at 200 h of observation. *Blue* and *red* colors represent cases with low (13.6 h) and high (61.3 h) initial cell cycle length, respectively.
5. Scatter plot of initial cell-cycle length and population size after 200 h. Strong negative correlation is observed (*ρ* = -0.65). Growth curves for two extreme cases. *Blue* and *red* colors represent cases with low (13.6 h) and high (61.3 h) initial cell-cycle length (respectively).
6. Descendants of ancestor cells are identified and counted. Growth curves show differences between two cell populations.

**S9 Fig Cell-cycle duration for across several generations.**

1. – (B) Ten extreme cases presented in the form of chart, where x axis represents generation number, y axis cell-cycle length.
2. – (D) Fifty extreme cases presented in the form of a heat map, where x axis represents generation number, y axis represent single-cell lineage and color denotes cell-cycle length.

**S10 Fig** **Scatter plots for cell-cycle length difference for pair of cousins and their physical distance.**

**S11 Fig** **Detailed scatterplots of experimental and simulated data for model parameters.**

**S12 Fig** **An example of “noisy” measurement.** Phase portraits for case where qualitative pattern is different than in majority of cells, it is caused by high noise level.

**S13 Fig** **Interaction between functional FUCCI proteins.** Cdt1 and its inhibitor Geminin are important regulators of replication licensing [[44](#_ENREF_44)]. In normal cells, a critical balance between these two proteins ensures that firing of each origin along the genome will take place only once per cell cycle. In our case we measure expression of dysfunctional proteins, but regulated in the same way as original ones.

Source: [[45](#_ENREF_45)].

**S14 Fig** **The second method of estimation of the cell-cycle endpoints.** It includes several steps: (1-2) identification of the level of noise and determination of the appropriate parameter values for smoothing, (local regression using weighted linear least squares and a 2nd degree polynomial model); (3) numerical differentiation of Geminin protein levels; (4) detection of local minima of differentiated data to identify division moments, and (5) detection of Cdt1 protein maxima, the timing of which provides the estimated moment of transition from G1 to S phase of cell cycle (in this step we analyze only fragment of Cdt1 protein dynamic located between division moments).

**S1 Data S_Data_15%_FBS_All_Cells.xlsx file** Measured intensities for Cdt1 and Geminin extracted from tracking (15% FBS).

**S1 Movie Changes of Cdt1 and Geminin protein across the cell cycle**. Black and blue dots represent experimental and simulation data, respectively.

**S1 Text Supplement-Mura-Feillet.docx.** The file contains additional results, discussion, description of methods and references.
